# A new plant SUMO ligase, MPEL1, synergizes with MAPK16 to regulate resistance against *Fusarium* pathogens

**DOI:** 10.1038/s44318-026-00811-2

**Published:** 2026-05-18

**Authors:** Xuanjun Feng, Weixiao Zhang, Huarui Guan, Haolan Yang, Na Wu, Yuting Chen, Xin Mao, Huihui Xiao, Mengling Li, Hao Xiong, Yinzhi Li, Zhaoyu Zhang, Muyan Liu, Li Jia, Shuang Cha, Yizhen She, Xuemei Zhang, Haicheng Liao, Guangchao Sun, Yaxi Liu, Wenming Wang, Yunbi Xu, Yuelin Zhang, Yanli Lu

**Affiliations:** 1 State Key Laboratory of Crop Gene Exploration and Utilization in Southwest China, Sichuan Agricultural University, Wenjiang, 611130 China; 2 Maize Research Institute, Sichuan Agricultural University, Wenjiang, 611130 China; 3 Peking University Institute of Advanced Agricultural Sciences, Weifang, 261325 China; 4 Key Laboratory of Bio-resource and Eco-environment of Ministry of Education, College of Life Sciences, Sichuan University, Chengdu, 610065 China

**Keywords:** Microbiology, Virology & Host Pathogen Interaction, Plant Biology, Post-translational Modifications & Proteolysis

## Abstract

SUMOylation is critical for plant growth and defense, and the specific substrate recognition mediated by SUMO ligases dictates the biological processes in which SUMOylation functions. However, only four SUMO ligases involved in SUMOylation have been reported in plants. Here, we report MPEL1 as a new SUMO ligase in maize, which paradoxically exhibits SUMO ligase activity despite its sequence homology to SUMO-targeted ubiquitin ligases (STUbL). MPEL1 stabilizes the plant-specific mitogen-activated protein kinase 16 (MAPK16) via SUMOylation at Lys526 within C-terminal domain (CTD). MAPK16 directly binds to JAZ20 via its C-terminal domain and phosphorylates JAZ20 at Thr12/Ser13 residues, thereby triggering its proteasomal degradation and activating broad-spectrum resistance against two Fusarium pathogens. These pathogens cause devastating maize ear rot and stalk rot, leading to severe yield losses and serious food safety concerns worldwide. Notably, *MAPK16* overexpression and *JAZ20* knockout enhance disease resistance without yield penalty, highlighting their potential for crop improvement. These findings expand the plant SUMO ligase family and provide targets for breeding Fusarium-resistant crops.

## Synopsis

Few SUMO E3 ligases from plants have been discovered so far. This work identifies the maize SUMO ligase MPEL1, which enhances Fusarium resistance via an MPEL1–MAPK16–JAZ20 immune pathway with no yield loss, offering ideal targets for crop improvement.

- MPEL1 is a novel plant SUMO ligase that stabilizes MAPK16 via SUMOylation at Lys526.
- MAPK16 directly phosphorylates JAZ20 at Thr12/Ser13 to promote its proteasomal degradation and activate antifungal defense.
- The MPEL1–MAPK16–JAZ20 module enhances maize resistance to both Fusarium verticillioides and Fusarium graminearum.
- Overexpressing MAPK16 or knocking out JAZ20 improves disease resistance without compromising maize yield.

## Abstract (web summary)

The MPEL1–MAPK16–JAZ20 immune pathway enhances maize disease resistance with no yield loss, offering ideal targets for crop improvement.

## Graphical abstract

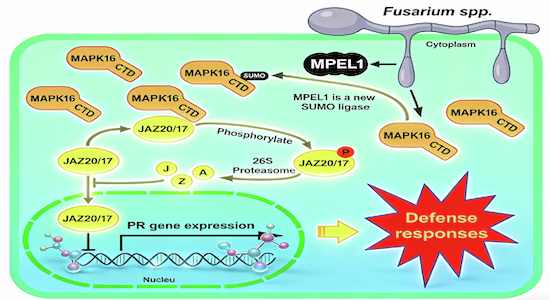

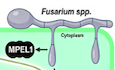

## Introduction

Protein post-translational modifications, particularly phosphorylation, ubiquitination, and SUMOylation, orchestrate developmental and defensive signaling in all eukaryotes (Arthur and Ley, 2013; Castaño-Miquel et al, 2017; Cheng et al, 2015; Verma et al, 2021; Zheng and Shabek, 2017). Phosphorylation cascades, exemplified by mitogen-activated protein kinase (MAPK) pathways, form the backbone of innate immunity (Arthur and Ley, 2013; Cheng et al, 2015). However, the plant-specific D-subgroup MAPKs, characterized by a unique TDY activation motif and an enigmatic C-terminal domain (CTD), remain poorly understood (Cheong et al, 2003; Dóczi et al, 2012), particularly in the context of disease resistance (Hong et al, 2019; Wang et al, 2018; Zhang and Zhang, 2022). Similarly, jasmonate (JA) signaling repressors (JAZ proteins) are critical defense modulators (Xu et al, 2026), yet the mechanisms regulating their stability, especially via MAPK-mediated phosphorylation, are largely uncharted. Although crosstalk between MAPK cascades and JA signaling is hypothesized, direct mechanistic links are rare (Katou et al, 2005; Takahashi et al, 2007; Wang et al, 2013; Zhang and Zhang, 2022). Although MAPKs often phosphorylate transcription factors to activate defense genes (Lin et al, 2022; Mao et al, 2011; Sun and Zhang, 2022; Zhang and Zhang, 2022), evidence directly connecting MAPKs to the phosphorylation of JAZ repressors is remarkably limited, with only one study implicating FvMAPK6 in phosphorylating FvJAZ12 (Wang et al, 2024).

SUMOylation, historically linked to nuclear processes, is increasingly recognized as a critical regulator of plant immunity (Castaño-Miquel et al, 2017; Niu et al, 2019; Orosa et al, 2018; Verma et al, 2021), often engaging in crosstalk with phosphorylation signaling either synergistically or antagonistically (Hendriks et al, 2017; Liu et al, 2023; Verma et al, 2021; Xie et al, 2025). Yet, the plant SUMO ligase repertoire remains small, with only four known members (SIZ1, MMS21, PIAL1, and PIAL2) (Jmii and Cappadocia, 2021). The discovery of SUMO-targeted ubiquitin ligases (STUbLs) revealed intricate crosstalk between SUMOylation and ubiquitination (Staudinger, 2017). STUbLs specifically recognize SUMOylated proteins and target them for proteasomal degradation (Staudinger, 2017). Consequently, their depletion leads to widespread accumulation of SUMOylated proteins (Kumar et al, 2017; Liu et al, 2024; Prudden et al, 2007; Sun et al, 2007). In yeast, STUbL activity requires a complex (Rfp1/2-Slx8), while in animals, fusion proteins like RNF4 perform this function independently (Sun et al, 2007; Xie et al, 2007). The roles and mechanisms of STUbL homologs in plants, however, are virtually unknown (Elrouby et al, 2013).

*Fusarium verticillioides* and *F. graminearum*, two major fungal pathogens of maize, cause devastating diseases leading to global yield losses of over 30% in severely affected regions (Lipps et al, 2025). Additionally, these pathogens contaminate grains with carcinogenic mycotoxins (e.g., fumonisins and deoxynivalenol), thereby posing severe threats to global food security and public health (Lipps et al, 2025). While genetic resistance represents the most sustainable strategy for managing these Fusarium-induced diseases, progress in developing durable resistance has been hindered by insufficient understanding of the underlying maize defense mechanisms. To date, only a handful of resistance genes have been functionally characterized (Dong et al, 2025; Li et al, 2024; Liao et al, 2023; Liu et al, 2025a; Liu et al, 2022; Ma et al, 2023; Ye et al, 2019).

Here, we demonstrate that *MAPK16* positively regulates both maize yield and resistance against Fusarium pathogens. Notably, overexpression of *MAPK16* does not compromise yield potential. Further investigation has identified a MAPK16-interacting E3 ligase (designated MPEL1), which mediates the SUMOylation of MAPK16 and enhances its protein stability. Interestingly, despite sharing sequence homology with STUBL, MPEL1 functions as a SUMO ligase. Mechanistically, we found that MAPK16 directly binds to JAZ20 via its C-terminal domain and phosphorylates JAZ20 at Thr12/Ser13 residues, thereby promoting JAZ20 degradation and subsequently activating defense responses against Fusarium pathogens.

## Results

### *MAPK16* positively regulates *F. verticillioides* resistance in maize

**Figure 1:**
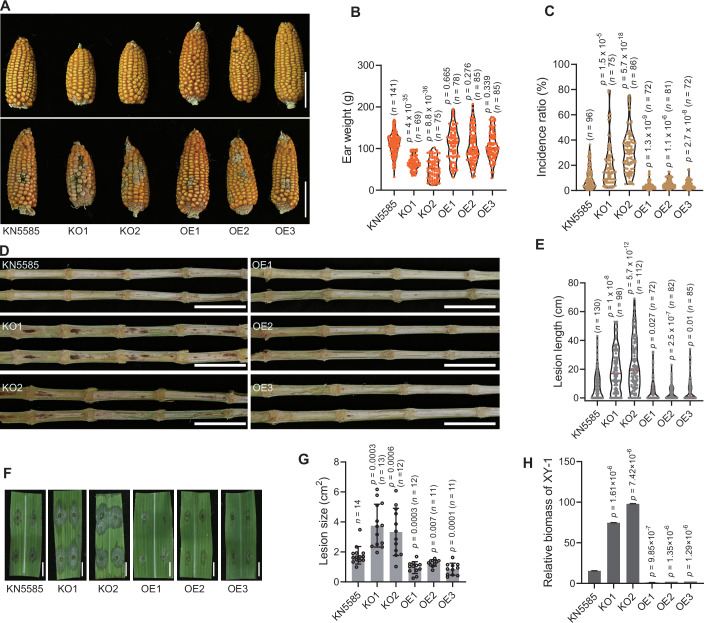
*MAPK16* positively regulates resistance against *F. verticillioides* in maize. (**A**‒**C**) Phenotype of uninfected and infected maize ears, with the weight of uninfected maize ears and incidence ratio of infected maize ears. Scale bar = 5 cm. (**D**,** E**) Phenotypes of infected stalks, with statistic value of lesion length. Scale bar = 10 cm. (**F**‒**H**) Phenotypes of infected leaves, with statistic value of lesion size per leaf. Scale bar = 1 cm. Relative biomass of *F. verticillioides* XY-1 in the infected leaves was tested by RT-qPCR. Gene *eF1a* was used to normalize the amount of plant tissue. Data from three biological replicates are presented in panels (**B**, **C**, **E**), with the median indicated by a short line. One representative result from three independent experiments with similar outcomes is shown in panels (**G**, **H**). Data were presented as mean ± SD in panel (**G**) and mean ± SEM in panel (**H**). Statistical analysis was performed via one-way ANOVA followed by Dunnett’s multiple comparisons test. Source data are available online for this figure.

**Figure EV1:**
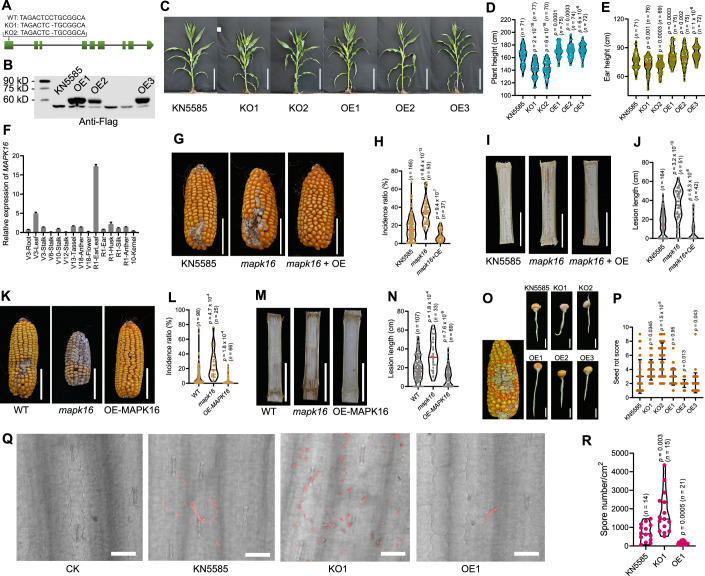
*MAPK16* is involved in the regulation of *F. verticillioides* resistance and maize development. (**A**) Schematic diagram of the gene structure of *MAPK16* and the editing types of the two knockout lines. (**B**) Protein expression levels in different transgenic overexpression lines. (**C**‒**E**) Plant phenotype and statistic values of plant height and ear height. Scale bar = 50 cm. (**F**) Transcriptional level of *MAPK16* in various tissues. *GAPDH* and *eF1α* were used as internal reference genes. Data represent mean ± SEM (*n* = 3). (**G**–**N**) Phenotypes and corresponding statistical values of *F. verticillioides* caused maize ear rot and stalk rot. (**G**‒**J**) The *mapk16* + *OE* represents overexpression of *MAPK16* in the genetic context of the *mapk16* mutant. Scale bar = 5 cm. (**K**‒**N**) *MAPK16* overexpression cassette and knockout mutation were introduced into the B73 genetic context by backcrossing. The *mapk16* mutants, *OE-MAPK16*, and wild-type plants were identified from the separation of offspring. Scale bar = 5 cm. (**O**,** P**) Seed rot was investigated using the seeds with no visible symptoms around the lesions, which are indicated with red asterisks, during seed germination. Scale bar = 1 cm. (**Q**,** R**) The colonization of spores on leaves. CK indicates that the detached leaves were exposed to *F. verticillioides* spores and immediately removed to a non-*F. verticillioides* conditions. Scale bar = 50 μm. Panels (**D**, **E**) represent three biological replicates; panels (**H**, **J**, **L**, **N**, **R**) show one representative result from three independent experiments with similar outcomes. Data in panels (**D**, **E**, **H**, **J**), **L**, **N**, **R**) are shown as violin plots, with the median indicated by a short line. Panel (**P**) shows one representative result from three independent experiments with similar outcomes. Data were presented as mean ± SD. Statistical analysis was performed via one-way ANOVA followed by Dunnett’s multiple comparisons test.

In our previous studies, *MAPK16* was a candidate gene associated with plant height and drought tolerance (Lu et al, 2010; Wang et al, 2019). In the field, we found that maize *mapk16* loss-of-function mutants were susceptible to ear rot, prompting an investigation into *MAPK16*’s role in resistance to *F. verticillioides*. Compared with wild-type plants, *mapk16* knockout (KO) mutants were more susceptible, whereas *MAPK16*-overexpressing (OE) plants were more tolerant to *F. verticillioides* (Figs. 1 and EV1). Notably, the effects of *MAPK16* were consistent in ears, stalks, and leaves (Fig. 1A,C‒H). Importantly, *MAPK16* loss-of-function was detrimental to yield and plant height, whereas overexpression of *MAPK16* slightly increased plant height but did not reduce yield (Figs. 1B and EV1A‒E). This suggests that MAPK16 is a promising target for breeding for resistance. High *MAPK16* expression in leaves, especially ear leaves, supports its role in maintaining yield (Fig. EV1F). The *F. verticillioides*-sensitive phenotype of *mapk16* mutants was rescued by complementary expression of *MAPK16* (Fig. EV1G‒J).

To test the function of MAPK16 across different genetic contexts, we introduced a MAPK16 overexpression cassette and knockout mutants into the B73 genetic background. The results revealed that its biological functions were similar to those observed in the KN5585 genetic background (Fig. EV1K‒N). *F. verticillioides* causes Fusarium ear rot (FER), characterized by the spread of hyphae through the cob and resulting in endophytes in seeds without visible symptoms. Therefore, we investigated germination symptoms using seeds with no visible symptoms around the lesions. KO and OE seeds exhibited more severe and milder symptoms, respectively, than wild-type seeds (Fig. EV1O,P). The invasion phase is critical for the host to develop resistance against hemibiotrophic pathogens, and successfully colonized pathogen spores can bind to host tissues via invasive hyphae (Jiang et al, 2020; Koeck et al, 2011). Spore colonization on leaves was investigated at 16 h post-infection (hpi). The number of spores was significantly higher in KO leaves and lower in OE leaves than that in wild-type leaves (Fig. EV1Q,R).

Furthermore, five pathogenesis-related (*PR*) genes and four lipoxygenase (*LOX*) genes were consistently detected in both OE and KO plants. Among these, *PR5, PR6, PR26*, and *chitinase 22* (*CHN22*) were induced significantly more strongly by *F. verticillioides* in OE plants compared with KO plants, whereas no differences in their expression levels were observed under pathogen-free conditions (Appendix Fig. S1). These findings indicate that MAPK16 overexpression does not constitutively activate basal immune responses under normal growth conditions—an observation that likely explains the absence of yield penalties in *MAPK16*-OE plants in the absence of pathogen infection. With respect to *LOX* family genes, *LOX1, LOX2*, and *LOX4* exhibited significantly higher induction by *F. verticillioides* in OE plants relative to KO plants, whereas *LOX3* induction was markedly reduced in OE plants upon pathogen challenge. This expression pattern aligns with the well-established roles of *LOX4* as a positive and *LOX3* as a negative regulator of Fusarium resistance in maize (Gao et al, 2007; Ottaviani et al, 2026), thereby reinforcing a functional connection between *MAPK16* and *LOX*-mediated defense pathways during *F. verticillioides* infection.

### MAPK16 interacts with MAPK16-interacting E3 ligase 1 (MPEL1) through its N-terminal domain

**Figure 2:**
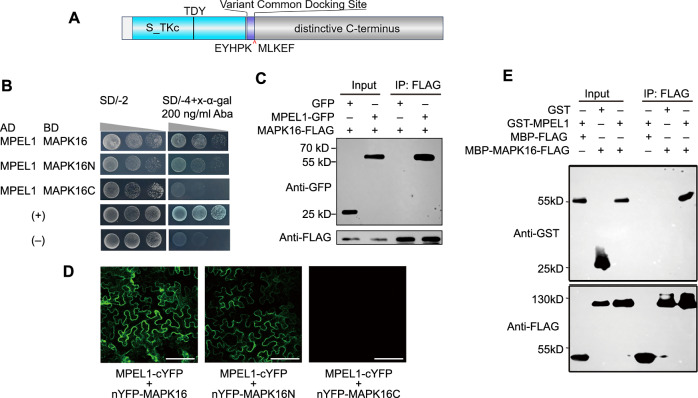
MAPK16 interacts with MPEL1 through its N-terminal domain. (**A**) Schematic diagram of MAPK16 protein. The N‑ and C‑termini represent the sequences upstream and downstream of the position marked by the red arrow, with the flanking five amino acid residues displayed. (**B**) Yeast two-hybrid assay of protein–protein interactions. The N-terminal domain and C-terminal domain of MAPK16 were cleaved at the position indicated by the red arrow in Fig. 2a. X-α-gal and Aba in SD/-4 medium refer to 5-Bromo-4-chloro-3-indole-α-D-galactopyranoside and Aureobasidin A, respectively. The combination of pGBKT7-53 and pGADT7-T served as a positive (+), whereas pGBKT7-Lam and pGADT7-T served as negative (‒) controls. (**C**) Co-immunoprecipitation assays for protein–protein interactions. Recombinant proteins were transiently expressed in tobacco leaves. (**D**) Bimolecular fluorescence complementation assay for protein–protein interactions. Scale bar = 50 μm. (**E**) Pull-down assay for protein–protein interactions. Recombinant proteins were expressed in *E. coli* and purified for corresponding analysis. Source data are available online for this figure.

To identify the interaction partners of MAPK16, we used its full-length coding sequence for yeast two-hybrid (Y2H) library screening (Fig. 2A). Thirteen prey proteins were initially identified, of which only two were validated by both Y2H and bimolecular fluorescence complementation (BiFC) assays (Table EV1). One of them was CNR8, and the other was a RING/U-box domain-containing protein. Since we did not detect phosphorylation of CNR8 by MAPK16 in this study, we did not pursue further investigations into this protein. Since the identified RING/U-box domain-containing protein was functionally uncharacterized, we named it MAPK16-interacting E3 ligase 1 (MPEL1). Further analysis indicated that MPEL1 binds to the N-terminus of MAPK16 (Fig. 2B,D). Co-immunoprecipitation (CoIP), BiFC, and pulldown assays further confirmed the interactions between MAPK16 and MPEL1 (Fig. 2C‒E). Subcellular localization assays showed that MAPK16 was primarily localized in the cytoplasm, whereas MPEL1 was distributed in both the cytoplasm and nucleus (Appendix Fig. S2).

In the aforementioned BiFC assays, tobacco leaves co-expressing MPEL1 and MAPK16 exhibited pronounced cell death at approximately 4 days post gene expression, suggesting a potential reactive oxygen species (ROS) burst. To investigate this further, we overexpressed MPEL1 and MAPK16 individually or co-expressed them in tobacco leaves. Co-expression of MPEL1 and MAPK16 consistently elicited significant cell death, whereas overexpression of MPEL1 alone induced milder cell death (Appendix Fig. S3A). In contrast, overexpression of MAPK16 or GFP did not induce cell death (Appendix Fig. S3A). Unexpectedly, overexpression of MAPK16 or MPEL1 did not stably affect chitin-induced ROS bursts in tobacco leaves, indicating that MPEL1-induced cell death may not be dependent on ROS (Appendix Fig. S3B,C). However, *MAPK16* had a positive and stable effect on chitin-induced ROS production in transgenic maize (Appendix Fig. S3D,E). Expression analysis revealed that *MAPK16* and *MPEL1* were transiently upregulated in response to *F. verticillioides* (Appendix Fig. S3F‒H).

### MPEL1 is a new SUMO ligase

**Figure 3:**
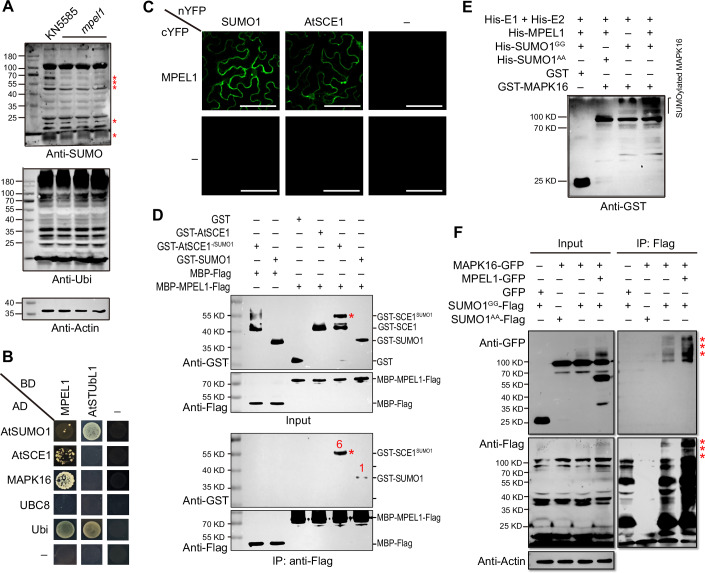
MPEL1 functions as a SUMO ligase rather than as a ubiquitin ligase. (**A**) Profiles of SUMOylation and ubiquitination in total leaf protein. Red asterisks indicate bands with significantly different intensities between *mpel1* mutants and wild type KN5585. (**B**) Yeast two-hybrid analysis of the interaction between MPEL1, AtSTUbL1, and members of the SUMO and ubiquitin systems. AtSCE1 and UBC8 serve as E2 conjugating enzymes for SUMO and ubiquitin, respectively. (**C**) Bimolecular fluorescence complementation assays for protein–protein interactions. Scale bar = 50 μm. (**D**) MPEL1 interacts with the covalent SUMO1-E2 conjugate. Protein pull-down analyzing MPEL1 interaction with SUMO1, AtSCE1, and pre-covalently linked SUMO1-AtSCE1 complex. GST-SCE1^-/SUMO1^ indicates that SCE1 is covalently coupled with SUMO1, resulting in both free SCE1 and SUMO1-modified SCE1. Red asterisk indicates SCE1 covalently coupled to SUMO1. The values above the GST-SCE1^-/SUMO1^ and GST-SUMO1 bands in the IP results represent the normalized signal intensities. Normalization was performed using the signal intensities of GST-SCE1^-/SUMO1^ and GST-SUMO1 bands in the Input, as well as the MBP-MPEL1-Flag signal in the IP. (**E**) In vitro SUMOylation assay using purified proteins. SUMO1^AA^ is a loss-of-function mutant of SUMO1 (namely SUMO1^GG^) that cannot covalently conjugate to substrates. (**F**) In vivo SUMOylation detection via transient expression in tobacco leaves. SUMOylated MAPK16 was indicated by the red asterisks on the right. Source data are available online for this figure.

**Figure EV2:**
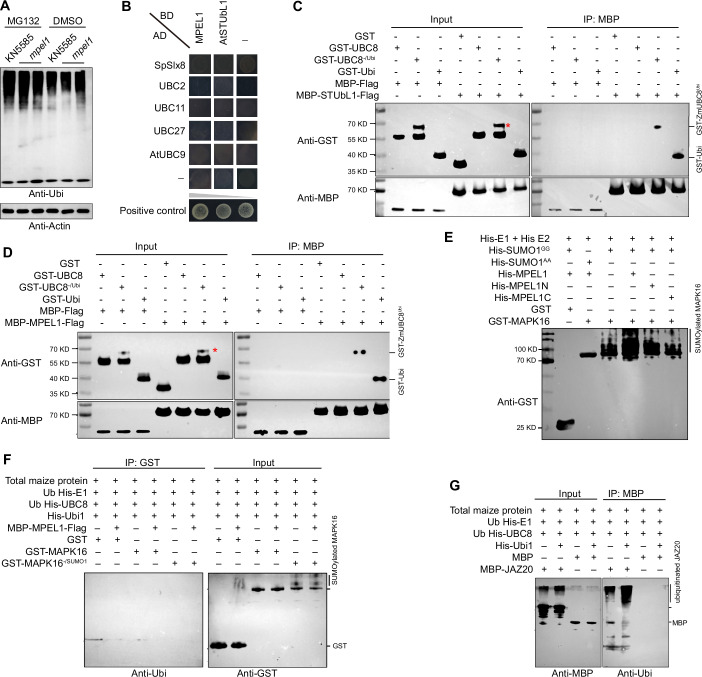
MPEL1 functions as a SUMO ligase rather than a ubiquitin ligase. (**A**) Profiles of ubiquitination in total root protein with or without the treatment of MG132. (**B**) Yeast two-hybrid analysis of the interaction between MPEL1, AtSTUbL1, yeast Slx8 and members of the ubiquitin systems. UBC2, UBC11, UBC27, and AtUBC9 serve as ubiquitin E2 conjugating enzymes. (**C**,** D**) Protein pulldown analyzing MPEL1 and AtSTUbL1 interaction with ubiquitin, UBC8, and pre-covalently linked Ubi-UBC8 complex. GST-UBC8^-/Ubi^ indicates that UBC8 is covalently coupled with ubiquitin, resulting in both free UBC8 and ubiquitin-modified UBC8. Red asterisk indicates UBC8 covalently coupled to ubiquitin. The values above the GST-UBC8^-/Ubi^ and GST-Ubi bands in the IP results represent the normalized signal intensities. Normalization was performed using the signal intensities of GST-UBC8^-/Ubi^ and GST-Ubi bands in the Input, as well as the MBP-MPEL1-Flag/ MBP-STUbL1-Flag signal in the IP. (**E**) In vitro SUMOylation assay using purified proteins. The C-terminal RING domain (residues 176 to the end) and the N-terminal SIM region (residues 1–175) of MPEL1 were analyzed. (**F**) Recombinant GST-MAPK16 was incubated with the SUMO system for one hour to get free GST-MAPK16 and SUMO1-modified GST-MAPK16, which were then affinity-purified using GST antibody-coated gel beads. The corresponding elution protein was subsequently incubated with other components for in vitro ubiquitination assays. (**G**) In vitro ubiquitination assays of JAZ20.

Sequence alignment analysis indicated that MPEL1 shares the highest similarity with AtSTUbL1 in Arabidopsis (Appendix Fig. S4A,B). Loss of STUbLs typically leads to the accumulation of SUMOylated proteins by impairing ubiquitin-dependent proteasomal degradation (Kumar et al, 2017; Liu et al, 2024; Prudden et al, 2007; Sun et al, 2007). Unexpectedly, however, loss of *MPEL1* led to a significant decrease in the intensity of multiple SUMOylated bands. At the same time, ubiquitination levels remained comparable to those of the wild type, suggesting that MPEL1 functions differently from STUbLs (Figs. 3A and EV2A). Supporting this, predictive analyses revealed that MPEL1 scored very low for the SUMO-interacting motif (SIM) (Appendix Fig. S4C).

In *Arabidopsis*, STUbL1 and STUbL3 have been reported to rescue the defective phenotype caused by the deletion of yeast Rfp1/2, a function analogous to that of human RNF4 (Elrouby et al, 2013). MPEL1 shares the highest similarity with Arabidopsis STUbL1; therefore, MPEL1 was subsequently comparatively studied alongside AtSTUbL1. In fission yeast, the SUMO-targeted ubiquitin ligase function is mediated by the Rfp1/2-Slx8 complex (Sun et al, 2007; Xie et al, 2007). Slx8 possesses intrinsic E3 ligase activity, whereas Rfp1/2 primarily recognize SUMO-modified proteins via SIM and enhance Slx8 activity (Sun et al, 2007; Xie et al, 2007). Sequence alignment showed that AtSTUbL1 and MPEL1 were more similar to Rfp2 than to Slx8 (Appendix Fig. S4B). However, the yeast two-hybrid assay results consistently demonstrated that neither AtSTUbL1 nor MPEL1 interacted with yeast Slx8, even when the bait/prey constructs were expressed at optimal levels (Fig. EV2B). AtSTUbL1 interacted strongly with SUMO1, whereas no interaction was detected between AtSTUbL1 and any of the five tested E2 ubiquitin-conjugating enzymes (Fig. 3B and Fig. EV2B,C). While both AtSTUbL1 and MPEL1 bound well to ubiquitin, neither showed enhanced binding to ubiquitin E2 conjugates (Figs. 3B and EV2B‒D).

Conversely, MPEL1 showed a weak interaction with SUMO1 but bound effectively to SUMO E2 conjugates (Fig. 3B‒D). These results imply that MPEL1 functions as a SUMO ligase, as the interaction between E3 and E2 relies on the formation of a covalent conjugate between E2 and SUMO or ubiquitin (Geiss-Friedlander and Melchior, 2007; Zheng and Shabek, 2017). We further confirmed the interaction between MPEL1 and the maize SUMO E2 enzyme (ZmSCE1), which was similar to that observed with AtSCE1, indicating that the function of MPEL1 is conserved in different species (Appendix Fig. S5A‒C). Furthermore, MPEL1 promoted SUMOylation of MAPK16 both in vitro (Fig. 3E) and in vivo (Fig. 3F), further confirming that MPEL1 acts as a SUMO ligase for MAPK16 rather than a STUbL. TOPORS was initially reported to exhibit SUMO ligase activity; however, a subsequent study showed that this activity was modest and independent of the RING domain (Park et al, 2008; Weger et al, 2005), indicating that TOPORS may play adapter-like or cofactor roles in SUMOylation. Therefore, we investigated whether the RING domain (residues 176 to the end) and the N-terminal SIM region (residues 1–175) were essential for the SUMO ligase activity of MPEL1. The results demonstrated that the SUMO ligase function of MPEL1 was dependent on its full-length protein; neither the isolated RING domain nor the N-terminal SIM region alone could promote the SUMOylation of MAPK16 (Fig. EV2E). Thus, the mechanism by which MPEL1 facilitates substrate SUMOylation is distinct from that of TOPORS, and MPEL1 likely acts as a bona fide SUMO ligase.

To further confirm that MPEL1 lacks ubiquitin ligase activity, we performed in vitro ubiquitination assays using non-SUMOylated MAPK16 and partially SUMOylated MAPK16. Since none of the tested E2 ubiquitin-conjugating enzymes (five members) interacted with MPEL1, we supplemented the reaction system with total maize proteins to provide potential components. The results showed that MPEL1 was unable to mediate the ubiquitination of MAPK16, regardless of whether MAPK16 was SUMOylated or not (Fig. EV2F). To verify the functionality of the ubiquitin-activating enzyme (E1), E2 enzymes, and Ubi1 used in this study, we examined the ubiquitination of JAZ20 using the same system, as JAZ proteins are known to be degraded via the ubiquitin-proteasome pathway. The results demonstrated that the in vitro ubiquitination system was fully functional (Fig. EV2G). To further confirm the SUMO ligase activity of MPEL1, we identified a novel interacting protein, autophagy-related protein 8b (ATG8b), and showed that MPEL1 bound to ATG8b and enhanced its SUMOylation (Appendix Fig. S6A,B).

### MPEL1 stabilizes MAPK16 through SUMOylation

**Figure 4:**
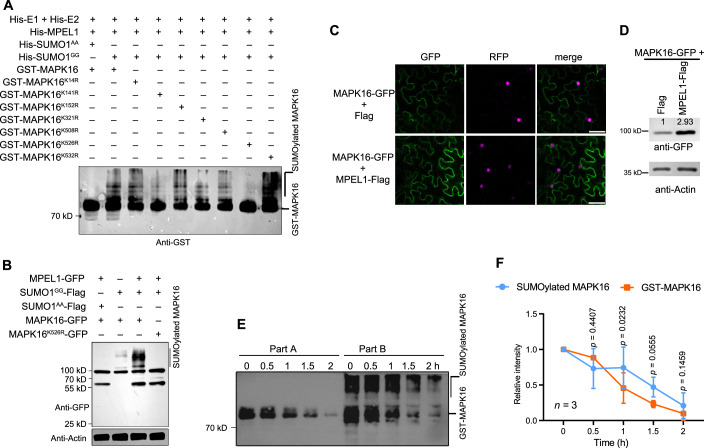
MPEL1 stabilizes MAPK16 by SUMOylation of its Lys526 amino acid residue. (**A**) GST-MAPK16 recombinants with distinct site-directed mutations were incubated with SUMO system in order to identify the SUMOylation site. (**B**) In vivo SUMOylation was detected via transient expression in tobacco leaves. Total proteins were extracted for western blot analysis after 36 h of expression. (**C**,** D**) Co‑expression of MPEL1 promotes protein accumulation of MAPK16 in the tobacco transient expression system. RFP was used as a nuclear localization marker. Imaging and western blot analysis were performed after 36 h of expression. Scale bar = 50 μm. (**E**,** F**) Recombinant GST-MAPK16 was incubated with the SUMO system for 2 h to get free GST-MAPK16 and SUMO1-modified GST-MAPK16, which were then affinity-purified using GST antibody-coated gel beads. The corresponding elution protein was subsequently incubated with the total protein of the wild-type maize. Equal volumes of protein solution were used for immunoblotting at different time points (Part B). At the same time, GST-MAPK16 was incubated with the total protein of the wild-type maize (Part A) as a control, demonstrating that no visible SUMOylated GST-MAPK16 could be formed under these in vitro conditions. Three biological replicates were conducted, and the signal intensity of the distinct protein bands of GST-MAPK16 and SUMOylated GST-MAPK16 (Part B) was calibrated using the corresponding zero-time point band as a reference in each replicate, and data were analyzed using a paired two-tailed Student’s *t*-test. Data were presented as mean ± SD. Source data are available online for this figure.

Analyses with SUMOplot (http://www.abgent.com/sumoplot), GPS-SUMO (https://sumo.biocuckoo.cn), and JASSA (http://www.jassa.fr/index.php?m=jassa) predicted that MAPK16 contain six SUMOylation motifs (YK^14^IE, LK^141^PK, CK^152^LK, CK^508^SE, PK^526^SY, and NK^532^LP). We performed in vitro SUMOylation assays using recombinant MAPK16 with different site-directed mutations to identify the primary SUMOylation site. The results showed that lysine 526 (Lys526) was the site of SUMOylation catalyzed by MPEL1 (Fig. 4A). We next investigated the in vivo SUMOylation of the MAPK16^K526R^ mutant. Our results showed that MAPK16^K526R^ was not modified by SUMO in tobacco cells, whereas wild‑type MAPK16 was efficiently SUMOylated (Fig. 4B).

To determine whether MPEL1 regulates the stability or subcellular localization of MAPK16, we performed co‑expression assays in tobacco leaves. Co‑expression of MPEL1 enhanced the accumulation of MAPK16 but did not alter its subcellular distribution (Fig. 4C,D). We further compared the stability of non‑SUMOylated and SUMOylated MAPK16. In an in vitro degradation assay using total protein extracts from wild‑type maize, SUMOylated MAPK16 was degraded significantly more slowly than its non‑SUMOylated form (Fig. 4E,F).

### MAPK16 interacts with JAZ20 through its distinctive C-terminal domain

**Figure 5:**
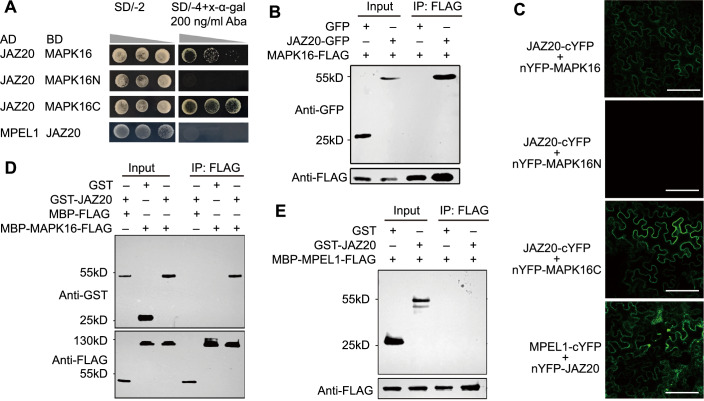
MAPK16 interacts with JAZ20 through its distinctive C-terminal domain. (**A**) Yeast two-hybrid assay of protein–protein interactions. The N-terminal domain and C-terminal domain of MAPK16 were cleaved at the position indicated by the red arrow in Fig. 2A. X-α-gal and Aba in SD/-4 medium refer to 5-Bromo-4-chloro-3-indole-α-D-galactopyranoside and Aureobasidin A, respectively. (**B**) Co-immunoprecipitation assays for protein–protein interactions. Recombinant proteins were transiently expressed in tobacco leaves. (**C**) Bimolecular fluorescence complementation assay for protein–protein interactions. Scale bar = 50 μm. (**D**,** E**) Pull-down assay for protein–protein interactions. Recombinant proteins were expressed in *E. coli* and purified for corresponding analysis. Source data are available online for this figure.

MAPK16 is a plant-specific D-subgroup MAPK protein characterized by a TDY motif, a canonical common docking site, and a distinctive C-terminal domain, which is predicted to function as a docking site for its target proteins (Fig. 2A) (Dóczi et al, 2012; Shi et al, 2022). The variant common docking site may explain why no MAPK kinases (MAPKKs) were identified from Y2H library screening, because the common docking site is reported to be responsible for interacting with MAPKKs (Dóczi et al, 2012). To investigate the potential role of the C-terminal domain of MAPK16, we performed Y2H library screening with the C-terminal domain of MAPK16 and initially identified 20 potential prey proteins (Table EV2). Considering the identification frequency of prey proteins and their established roles in defense responses, we selected five candidates for follow-up validation assays, with only JAZ20 being successfully verified (Fig. 5A). Notably, we found that the binding affinity of JAZ20 to full-length MAPK16 was significantly lower than that to the C-terminal domain of MAPK16 (Fig. 5A,C). CoIP, BiFC, and pull-down assays further confirmed the interaction between MAPK16 and JAZ20 (Fig. 5B‒D). MPEL1 and JAZ20 did not interact directly, but they form an indirect complex in plant cells (Fig. 5C,E), which may be attributed to the fact that JAZ20 and MPEL1 could interact with the C-terminal domain (Fig. 5A,C) and N-terminal domain (Fig. 2B,D) of MAPK16, respectively. We further investigated whether MPEL1 could SUMOylate JAZ20 with or without MAPK16 mediation and found that MPEL1 was unable to promote the SUMOylation of JAZ20 under either condition (Appendix Fig. S7).

### MAPK16 promotes the degradation of JAZ20 through phosphorylation

**Figure EV3:**
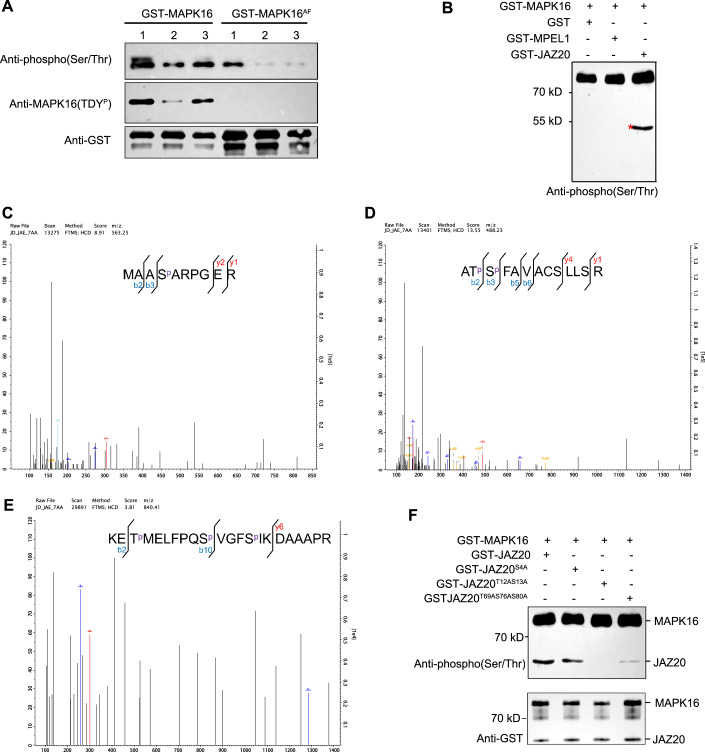
JAZ20 is a phosphorylation substrate of MAPK16. (**A**) MAPK16 autophosphorylates its TDY motif. For each protein, lane 1 shows the protein directly purified from prokaryotic expression. Lanes 2 and 3 represent proteins first dephosphorylated with λ-protein phosphatase (λ-PPase), followed by in vitro phosphorylation assays performed in the absence or presence of ATP, respectively. MAPK16^AF^ denotes the MAPK16 protein in which threonine at position 175 and tyrosine at position 177 are mutated to alanine and phenylalanine (TDY → ADF), respectively. (**B**) In vitro phosphorylation modification assays for GST-JAZ20 and GST-MPEL1. The band indicated by the red star is phosphorylated GST-JAZ20. (**C**‒**E**) A total of six sites in three peptides from JAZ20 were identified by mass spectrometry as possible phosphorylation targets for MAPK16. More details are shown in Dataset EV1. (**F**) Recombinant GST-JAZ20 with different site-directed mutations were incubated with recombinant GST-MAPK16, and phosphorylation modifications were detected by immunoblotting.

**Figure 6:**
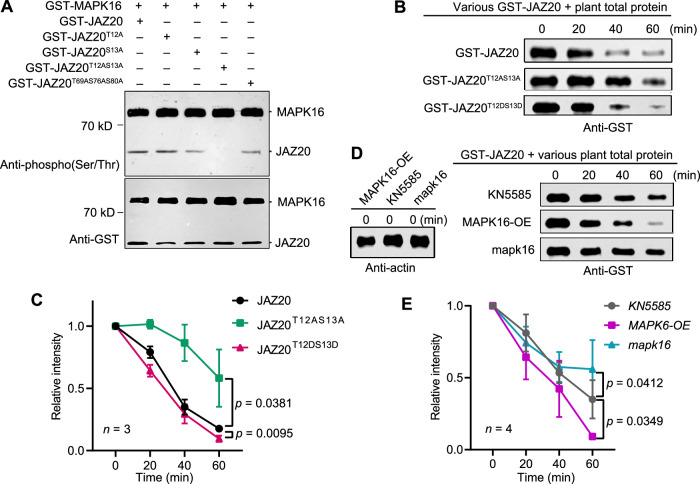
MAPK16 promotes the degradation of JAZ20 by phosphorylating its Thr12 and Ser13 amino acid residues. (**A**) GST-JAZ20 with different site-directed mutations were incubated with GST-MAPK16, and phosphorylation modifications were detected by immunoblotting. (**B**,** C**) Equal quantities of recombinant GST-JAZ20 with different site-directed mutations were incubated with the total protein of the wild-type maize. Thereafter, equal volumes of protein solution were used for immunoblotting at different time points. Three biological replicates were performed, and the signal intensity of different protein bands was calibrated using the zero-time point band as a reference in each replicate, and data were analyzed using a paired two-tailed Student’s t-test. (**D**,** E**) Equal quantities of recombinant GST-JAZ20 were incubated with the total protein of *MAPK16* overexpressing plants, knockout mutants, and the wild-type. Then, equal volumes of protein solution were used for immunoblotting at different time points. Four biological replicates were performed, and the signal intensity of different protein bands was calibrated using the zero-time point band as a reference in each replicate, and data were analyzed using an unpaired two-tailed Student’s *t*-test. Data were presented as mean ± SD in panels (**C**, **E**). Source data are available online for this figure.

We evaluated whether JAZ20 and MPEL1 were phosphorylation targets of MAPK16. MAPK16 exhibited strong autophosphorylation of the TDY motif within its activation loop (Fig. EV3A). In line with this, immunoblotting results indicated that MAPK16 directly phosphorylated JAZ20 in vitro, whereas MPEL1 remained unphosphorylated (Fig. EV3B). Next, the potential phosphorylation sites of JAZ20 were identified using mass spectrometry, and the six most likely sites were detected (Fig. EV3C‒E; Dataset EV1). Recombinant JAZ20 proteins with different site-directed mutations were used to confirm the phosphorylation sites. The results showed that threonine 12 (Thr12) and serine 13 (Ser13) were the dominant phosphorylation sites catalyzed by MAPK16, and mutations at the Thr12 or Ser13 sites alone had little effect on the phosphorylation of JAZ20 (Figs. 6A and EV3F). The effect of phosphorylation on the stability of JAZ20 was investigated. Mimicking phosphorylation by mutating Thr12 and Ser13 to aspartic acid accelerated JAZ20 degradation (Fig. 6B,C). In contrast, substituting these residues with alanine delayed JAZ20 degradation (Fig. 6B,C). When recombinant JAZ20 was incubated with total protein extracts from MAPK16-OE plants, JAZ20 degraded more rapidly, whereas incubation with extracts from *mapk16* mutants slowed JAZ20 degradation (Fig. 6D,E).

**Figure 7:**
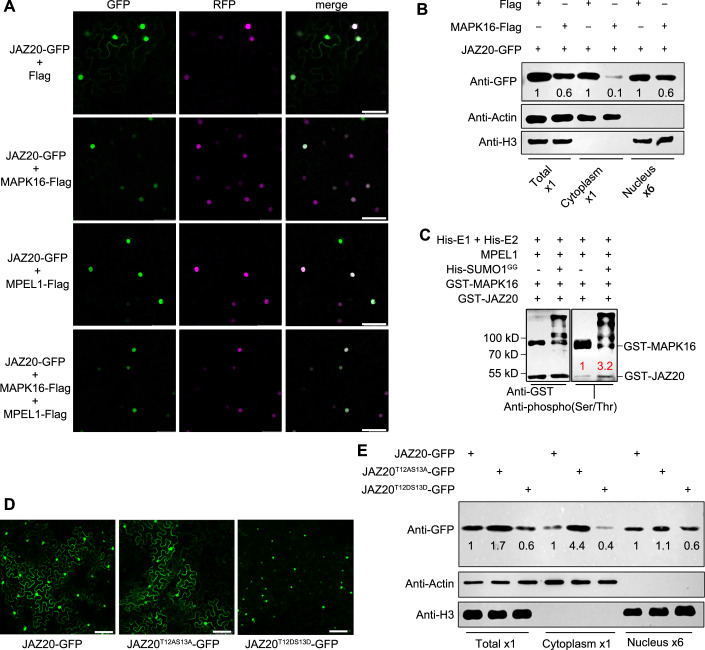
MAPK16 and MPEL1 primarily promote JAZ20 degradation rather than altering its subcellular localization. (**A**) JAZ20-GFP was transiently expressed in combination with MPEL1-Flag, MAPK16-Flag, MPEL1-Flag and MAPK16-Flag, or Flag in tobacco leaves for 36 h to investigate the effect of MAPK16 and/or MPEL1 on JAZ20. RFP represents a nuclear localization marker. Scale bar = 50 μm. (**B**) JAZ20-GFP, transiently co-expressed with MAPK16-Flag or Flag in tobacco leaves for 36 h, was used for protein fractionation assays. The total amount of JAZ20-GFP was normalized to the signal intensity of the cytoplasmic marker protein Actin and the nuclear marker protein H3. The levels of JAZ20-GFP in the cytoplasmic and nuclear fractions were normalized to their respective markers, Actin and H3. The relative amounts of JAZ20-GFP protein in different samples are presented, with the first lane of each fraction serving as a calibration reference. Note that the nuclear fraction was concentrated sixfold relative to the cytoplasmic and total fractions. (**C**) Equal quantities of GST-JAZ20 were incubated with GST-MAPK16 and His-MPEL1 under conditions with or without His-SUMO1^GG^, and the amount of phosphorylated JAZ20 under the two conditions was compared. (**D**, **E**) JAZ20-GFP, mutated at the Thr12 and Ser13 sites, was transiently expressed in tobacco leaves and used for protein fractionation assays. The relative amount of JAZ20-GFP in different fractions was calculated in a manner similar to that described in Fig. 7B. Scale bar = 50 μm. Source data are available online for this figure.

We investigated whether MAPK16 or MPEL1 affected the subcellular localization of JAZ20. Co-expression of MAPK16 and/or MPEL1 led to a significant reduction or near absence of cytoplasmic JAZ20 protein. In contrast, the fluorescence intensity of nuclear JAZ20-GFP remained largely unchanged (Fig. 7A). To explore this further, we subjected transiently expressed JAZ20-GFP in tobacco leaves to protein fractionation assays. Immunoblot analysis of the cytoplasmic, nuclear, and total fractions revealed that co-expression with MAPK16 significantly decreased the overall abundance of JAZ20-GFP, with a greater reduction observed in the cytosolic fraction (Fig. 7B). The coexistence of MAPK16 and MPEL1 enhanced the phosphorylation of JAZ20 in vitro compared with MAPK16 alone (Fig. 7C). We also examined JAZ20-GFP mutants with altered phosphorylation sites (Thr12 and Ser13). The phosphomimetic mutant JAZ20^T12DS13D^-GFP showed significantly lower abundance across all fractions than wild-type JAZ20-GFP, whereas the phosphodeficient mutant JAZ20^T12AS13A^-GFP exhibited significantly higher abundance (Fig. 7D,E). These findings indicate that MAPK16 and MPEL1 primarily promoted JAZ20 degradation rather than altered its subcellular localization. Furthermore, treatment with MG132, a 26S proteasome inhibitor, increased the abundance of both JAZ20^T12AS13A^-GFP and JAZ20^T12DS13D^-GFP, with a more pronounced effect observed in the phosphomimetic mutant (Appendix Fig. S8). This suggests that the enhanced protein degradation of JAZ20 following phosphorylation is mediated via the 26S proteasome pathway.

### The *MPEL1-MAPK16-JAZ20* signaling cascade regulates resistance against *F. verticillioides and F. graminearum* in maize

**Figure EV4:**
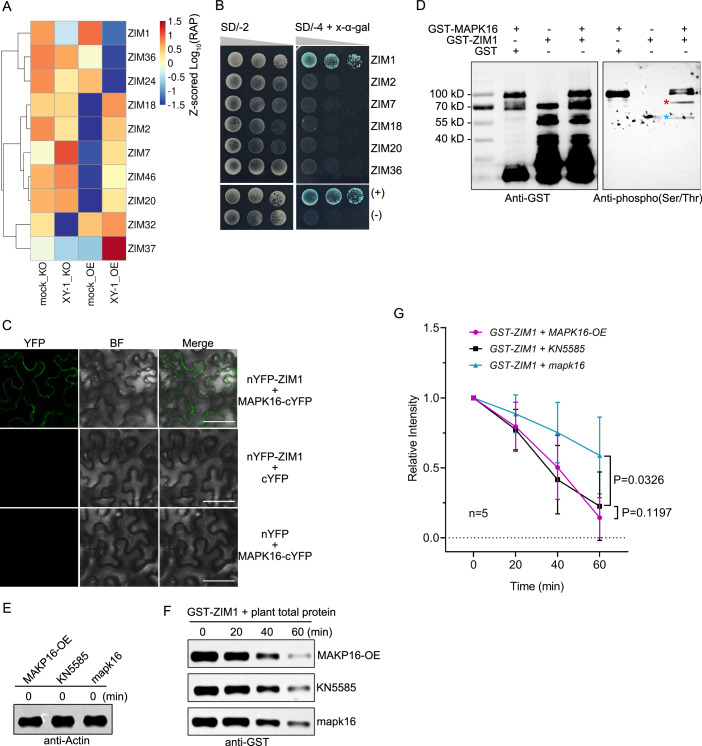
ZIM1 (also known as JAZ17) is another target of MAPK16. (**A**) The average expression levels of the ZIM family proteins identified in the comparative proteomic experiments are presented in a heat map. A log10 conversion and Z-score normalization of the average relative abundance of the proteins (RAP) were performed before heat mapping. (**B**) Yeast two-hybrid was used to analyze the interaction of MAPK16 with various ZIMs. (**C**) Bimolecular fluorescence complementation assay for protein–protein interactions. Scale bar = 50 μm. (**D**) In vitro phosphorylation modification assays for GST-ZIM1. The band marked with a red asterisk represents phosphorylated GST-ZIM1, while the band indicated by the blue asterisk may correspond to partially degraded and phosphorylated GST-ZIM1. (**E**‒**G**) Equal quantities of recombinant GST-ZIM1 were incubated with the total protein of *MAPK16* overexpressing plants, knockout mutants, and the wild-type. Then, equal volumes of protein solution were used for immunoblotting at different time points. Five biological replicates were performed, and the signal intensity of different protein bands was calibrated using the zero-time point band as a reference in each replicate, and data were analyzed using a paired two-tailed Student’s *t*-test. Data were presented as mean ± SD.

We further performed comparative proteomic analyses between *MAPK16-*OE and *MAPK16-*KO plants (Dataset EV2). Ten ZIM domain-containing transcription factors were identified as differentially expressed, whereas only ZIM1 (also known as JAZ17) was found to interact with MAPK16 in yeast two-hybrid assays (Fig. EV4A,B). This interaction was further confirmed using BiFC assays (Fig. EV4C). In addition, MAPK16 positively regulated the phosphorylation and degradation of ZIM1 (Fig. EV4D‒G), similar to its effect on JAZ20.

In the absence of *F. verticillioides* infection, overexpression of *MAPK16* did not induce the upregulation of defense-related genes (Dataset EV3). These findings further indicate that overexpression of *MAPK16* does not lead to excessive activation of the immune response under normal conditions. In contrast, under *F. verticillioides* stress, the genes upregulated by MAPK16 overexpression were mainly enriched in amine metabolism, tryptophan metabolism, and hydrogen peroxide metabolism pathways—biological processes typically involved in plant defense responses (Dataset EV3). Notably, our previous study demonstrated that histamine, an amine metabolite, enhances resistance to *F. verticillioides* in maize (Feng et al, 2025b). In contrast, the proteins downregulated upon MAPK16 overexpression are primarily involved in plant growth and development processes, such as DNA replication, chromosome organization, and nucleic acid metabolism (Dataset EV3).

**Figure 8:**
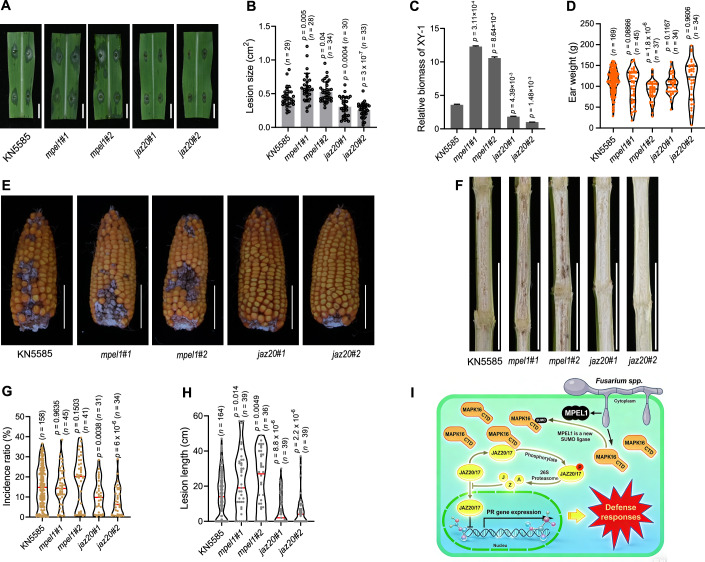
MPEL1 and JAZ20 play positive and negative role, respectively, in maize resistance against *F. verticillioides.* (**A**,** B**) Phenotypes of infected leaves, with statistic value of lesion size. (**C**) Relative biomass of *F. verticillioides* XY-1 in the infected leaves was tested by qRT-PCR. Gene *eF1a* was used to normalize the amount of plant tissue. (**D**) Dry weight of maize ear. (**E**‒**H**) Phenotype of *F. verticillioides* infected maize ears and stalks, with the incidence ratio of infected maize ears and lesion length of stalks. Scale bars represent 1, 5, and 10 cm for 6A, E, and F, respectively. Panels (**B**–**D**) and (**G**,** H**) show one representative result from three independent experiments with similar outcomes. Data are presented as mean ± SD in panel (**B**) and mean ± SEM in panel (**C**). Data in panels (**D**, **G**, **H**) are shown as violin plots, with the median indicated by a short line. Statistical analysis was performed via one-way ANOVA followed by Dunnett’s multiple comparisons test. (**I**) A proposed working model for *MPEL1*, *MAPK16*, and *JAZ20* illustrates their roles in conferring resistance to *Fusarium* pathogens. Source data are available online for this figure.

It has been widely established that JAZ members are extensively involved in the negative regulation of plant defense responses (Fernandes and Ghag, 2022). As expected, loss of *JAZ20* function enhanced maize resistance to *F. verticillioides* (Fig. 8A‒I; Appendix Fig. S9B). In contrast, loss of *MPEL1* impaired maize resistance to *F. verticillioides* (Fig. 8A‒I; Appendix Fig. S9A). Notably, the decrease in Fusarium resistance caused by loss of *MPEL1* was inconsistent and did not have a significant effect on ear rot (Fig. 8G). Loss of *JAZ20* function also did not affect maize yield (Fig. 8D), showing effects similar to those of *MAPK16* overexpression. Chitin-induced ROS levels remained unaffected in both *mpel1* and *jaz20* mutants (Appendix Fig. S9C,D). We further investigated the effect of *JAZ20* deficiency on the expression of five defense marker genes. Quantitative real-time PCR (qRT-PCR) analysis showed that *JAZ20* knockout promoted the transcription of *PR5* and *CHN22* but had no effect on that of *PR6* and *PR26* (Appendix Fig. S9E). These findings indicate that the enhanced resistance to *F. verticillioides* mediated by *MAPK16* is not solely achieved through the regulation of *JAZ20*.

**Figure EV5:**
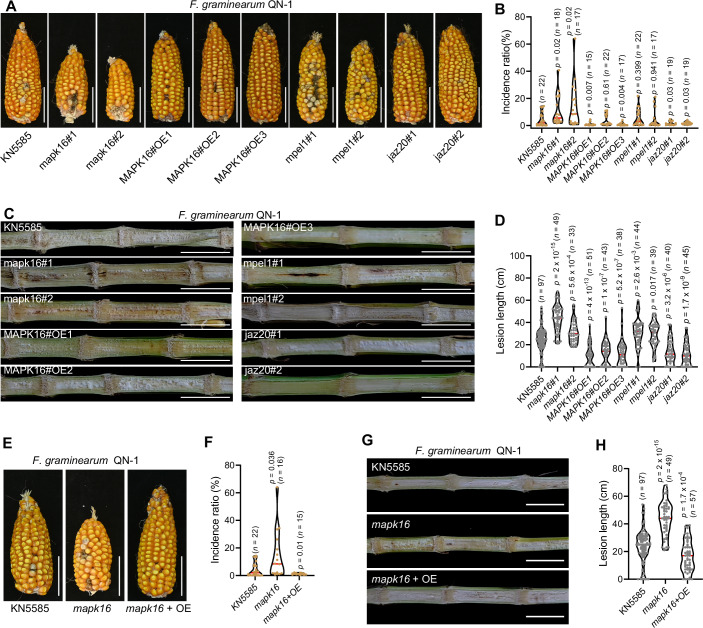
Gene module *MPEL1-MAPK16-JAZ20* also regulates resistance against *F. graminearum* in maize. (**A**‒**H**) Phenotypes and corresponding statistical values of *F. graminearum* caused maize ear rot and stalk rot. *mapk16#1* and *mapk16#2* are CRISPR knockout mutants. (**E**‒**H**) The *mapk16* + *OE* represents overexpression of *MAPK16* in the genetic context of the *mapk16* mutant. Panels (**B**, **D**) show one representative result from three independent experiments with similar outcomes. Panels (**F**, **H**) show one representative result from two independent experiments with similar outcomes. Data are shown as violin plots, with the median indicated by a short line. Data were analyzed using an unpaired two-tailed Student’s *t*-test. Scale bars = 5 cm.

Given that *F. graminearum* is another major pathogen causing ear rot in maize, we also investigated the roles of *MPEL1, MAPK16*, and *JAZ20* in resistance to *F. graminearum*. The results showed that the effects of these genes on resistance to *F. graminearum* mirrored their effects on resistance to *F. verticillioides* (Fig. EV5A‒H).

## Discussion

### *MAPK16* and *JAZ20* are key targets for Fusarium resistance breeding

Although D-subgroup members constitute half of the total MAPK repertoire in plants (Shi et al, 2022), research on this subgroup is considerably less extensive than that on the A, B, and C subgroups—with the functions of their C-terminal domains still largely elusive (Cheong et al, 2003; Dóczi et al, 2012). As a poorly characterized member of the plant-specific D-subgroup MAPKs, MAPK16 harbors a distinctive C-terminal domain that endows it with multiple critical functions, serving as a core positive regulator of maize resistance against *F. verticillioides* and *F. graminearum*. This domain not only acts as the site for MPEL1-mediated SUMOylation at Lys526 (regulating MAPK16 function by enhancing its protein stability) but also serves as a binding site for JAZ family proteins (JAZ20 and JAZ17), providing dual mechanisms for substrate recruitment and self-stability regulation.

Jasmonic acid (JA) signaling is pivotal for plant defense responses (Fernandes and Ghag, 2022; Xu et al, 2026). Under resting conditions, JAZ proteins, together with the adapter protein NOVELINTERACTOR OF JAZ and co-repressor TOPLESS, associate with MYC transcription factors and repress their activity. Upon biotic challenge, JAZ proteins are ubiquitinated by the SCF^COI1^ E3 ubiquitin ligase complex and degraded, releasing MYCs to activate defense genes (Xu et al, 2026). MAPK16 phosphorylates JAZ20 at Thr12 and Ser13, triggering its 26S proteasome-mediated degradation—a mechanism that aligns with the well-established negative regulatory role of JAZ family proteins in plant defense responses. The negative regulatory role of JAZ family members in resistance against *F. oxysporum* has been extensively documented in various plants (Fernandes and Ghag, 2022). In maize, exogenous application of methyl jasmonate enhances resistance against *F. graminearum* via the *ZmCOI1a-ZmJAZ15* module. Degradation of ZmJAZ15 activates the expression of downstream defense genes and promotes phenylpropanoid accumulation (Ma et al, 2021). Similarly, *ZmLOX4* and *ZmLOX12* enhance defense against *F. verticillioides* by promoting the biosynthesis of 12-oxo-phytodienoic acid and downstream JA production (Christensen et al, 2014; Ottaviani et al, 2026).

Meanwhile, the favorable characteristics of *MAPK16* make it an ideal target for disease resistance breeding. Overexpression of *MAPK16* significantly enhances the broad-spectrum resistance of maize to two Fusarium species without compromising yield. Similarly, *JAZ20* knockout mutants exhibit improved disease resistance without compromising yield. This result is consistent with the finding that overexpression of *MAPK16* did not induce the upregulation of defense-related genes in the absence of *F. verticillioides* infection.

### *MPEL1* is a new SUMO ligase

Functional characterization of MPEL1 challenges the traditional understanding of STUbL homologs: while sharing sequence homology with STUbL family proteins (particularly AtSTUbL1), it lacks ubiquitin ligase activity and instead exhibits canonical SUMO ligase function. This functional repurposing is not an isolated case (Frost et al, 2012; Gabaldón and Koonin, 2013), suggesting that plant STUbL homologs may have undergone extensive functional divergence from their animal counterparts. Moreover, MPEL1 is distinguished from known plant SUMO ligases (such as SIZ1, MMS21, and PIAL1/2) by its sequence and predominant cytoplasmic localization, while still sharing the RING domain typical of these ligases (Jmii and Cappadocia, 2021). Notably, MPEL1 physically interacts with MAPK16 in the cytoplasm, a finding that deviates from the canonical nuclear localization of most SUMOylation events in plants. This cytoplasmic SUMOylation of MAPK16 by MPEL1 represents a novel non-canonical SUMOylation regulatory mode in plant defense signaling, which may be tailored to the cytoplasmic localization of MAPK16 and its role in mediating rapid defense responses against Fusarium pathogens. Unlike nuclear SUMOylation that often modulates transcriptional regulation, cytoplasmic SUMOylation of MAPK16 by MPEL1 directly enhances the protein stability of this core kinase, enabling the rapid activation of downstream JA signaling by phosphorylating JAZ20 without the need for nuclear-cytoplasmic shuttling—an adaptive mechanism that may facilitate the prompt initiation of defense responses upon fungal invasion. The functions of the Arabidopsis AtSTUbL family remain incompletely understood. Elrouby et al, (2013) reported that AtSTUbL1/3 can complement the phenotypic defects of yeast Rfp1/2 deletion mutants (Elrouby et al, 2013), but this study found that AtSTUbL1 does not interact with yeast Slx8 nor exhibit ubiquitin ligase activity, which sharply contrasts with the SUMO ligase function of MPEL1. These findings indicate that the functions of Arabidopsis STUbL family members are not yet fully defined, and their precise roles in plants require further exploration.

MPEL1 stabilizes MAPK16 through SUMOylation, thereby enhancing downstream disease resistance signaling. However, loss of *MPEL1* impairs Fusarium resistance only in tissues such as stalks and leaves, with no significant effect on ear resistance. This phenomenon is primarily attributed to intrinsic divergence in maize defense mechanisms against Fusarium across different organs. Xiong et al reported that maize stems and roots are the main “battlefields” for coevolution with *F. verticillioides* (Xiong et al, 2024). Additionally, ear resistance to Fusarium is predominantly mediated by induced defense with a transient nature, whereas root/stalk resistance is underpinned by constitutive structural reinforcement, rhizosphere microbiota modulation, and phenylpropanoid metabolism (Lanubile et al, 2017; Xia et al, 2024; Xiong et al, 2024; Xu et al, 2025). This implies that MPEL1 may exhibit tissue-specific differences in expression levels or functional relevance, which likely contribute to its disparate effects on resistance across maize organs. Furthermore, as a SUMO ligase, MPEL1 is predicted to target multiple substrates (e.g., ATG8b identified in this study), endowing it with multifaceted regulatory roles in plant immunity. The cell death observed in *N. benthamiana* upon MPEL1-MAPK16 co-expression is a reflection of this multifaceted regulation. Specifically, while MPEL1-mediated SUMOylation of MAPK16 boosts Fusarium resistance by stabilizing the core kinase and sustaining JA signaling cascade activation, its SUMOylation of other uncharacterized substrates may trigger excessive cell death. This is particularly detrimental for defense against hemibiotrophic Fusarium pathogens, which can exploit nutrient-rich necrotic tissue to facilitate colonization and proliferation (Liu et al, 2025b). The lack of a significant effect of MPEL1 loss-of-function on ear resistance may stem from the opposing roles of MPEL1-mediated SUMOylation on distinct substrates—either promoting or suppressing immune responses (Castaño-Miquel et al, 2017; Niu et al, 2019; Orosa et al, 2018; Verma et al, 2021). We hypothesize that MPEL1 may resolve this regulatory balance via a SUMOylation-ATG8b axis, coupling SUMO modification to ATG8b-mediated autophagy and MAPK16-driven defense activation. This coordination fine-tunes the extent of cell death and defense signaling, thereby avoiding detrimental excessive necrosis while sustaining effective resistance against Fusarium pathogens.

Collectively, this study identifies MPEL1 as a new SUMO ligase and uncovers the core role of the “MPEL1-MAPK16-JAZ20” regulatory pathway in maize resistance against Fusarium pathogens. Specifically, MPEL1 stabilizes MAPK16 via cytoplasmic SUMOylation; the accumulated MAPK16 then phosphorylates and degrades JAZ20, a negative regulator of JA signaling. This process relieves the inhibition of JA signaling, thereby activating broad-spectrum disease resistance in maize. The core values of this research are threefold. First, it provides a reliable case for elucidating the function of the C-terminal domain in the D-subgroup MAPK family. Second, it establishes a new functional paradigm for STUbL homologs: as a new SUMO ligase, MPEL1 expands the plant SUMO ligase family, laying an important foundation for re-evaluating the functional diversity of STUbL family members. Third, it identifies MAPK16 and JAZ20 as key targets for maize Fusarium resistance breeding, given that their overexpression or knockout enhances maize resistance to *Fusarium* without sacrificing yield.

## Methods

**Table:** Reagents and tools table

Reagent/resource	Reference or source	Identifier or catalog number
**Experimental Models**		
Ubi::MAPK16-FLAG	This paper	N/A
CRISPR-Cas9: *mapk16*	This paper	N/A
CRISPR-Cas9: *jaz20*	This paper	N/A
CRISPR-Cas9: *mpel1*	This paper	N/A
B73 and KN5585	Wild-type maize inbred lines	N/A
*Fusarium. verticillioides* XY-1	Xiong et al, 2024	N/A
*Fusarium. graminearum* QN-1	Xiong et al, 2024	N/A
Trans1-T1 Phage Resistant Chemically Competent Cell	TransGen Biotech	Cat# CD501-02
TransB(DE3) Chemically Comptent Cell	TransGen Biotech	Cat# CD811-02
GV3101 Electroporation-Competent Cell	AngYuBio	Cat# G6039
Y2HGold Yeast Strain	Clontech	Cat# 630498
**Recombinant DNA**		
pLANT-cFlag	All vector skeletons were provided by the State Key Laboratory of Crop Gene Exploration and Utilization in Southwest China, Sichuan Agricultural University	N/A
pCAMBIA1300		
pGBKT7		
pGADT7		
pGEX-6P-1		
pET-28a		
pMAL-c2X		
pXYc104		
PXYn106		
His-SUMO1^AA^	Qu et al, 2020	N/A
His-SUMO1^GG^		
SUMO E1		
SUMO E2		
**Antibodies**		
Monoclonal ANTI-FLAG® M2 antibody	Sigma-Aldrich	Cat# F3165; RRID: AB_259529
Anti-GFP rabbit polyclonal antibody	Sangon Biotech	Cat# D110008
GST-tag Mouse mAb	Yeasen	Cat#30901ES50
MBP-Tag Polyclonal antibody	Proteintech Group	Cat#15089-1-AP; RRID: AB_1607774
Ubiquitin Rabbit mAb	Proteintech Group	Cat# 10201-2-AP; RRID: AB_671515
Rabbit Polyclonal SUMO1 antibody	Abcam	Cat# ab5316; RRID: AB_2198088
Anti-Phosphoserine/threonine	ECM Biosciences	Cat# PP2551; RRID: AB_1184778
Anti-MAPK16(TDY^P^)	This paper	ABclone
GAOT ANTI MOUSE IgG HRP	Bio-Rad	Cat# 1706516; RRID: AB_2921252
Goat Anti-Rabbit IgG (H + L)-HRP Conjugate	Bio-Rad	Cat# 1706515; RRID: AB_11125142
**Oligonucleotides and other sequence-based reagents**		
Primers for the cloning of the coding sequences from the gene cDNA	This paper, Dataset EV4	N/A
Primers for vector construction	This paper, Dataset EV4	N/A
Primers for PCR, qPCR and KASP	This paper, Dataset EV4	N/A
**Chemicals, Enzymes and other reagents**		
KOD FX Neo	TOYOBO	Cat# KFX-201
Restriction enzyme (BamH I, et al)	NEB	Cat# R0136V
FastPure Gel DNA Extraction Mini Kit	Vazyme	Cat# DC301-01
ClonExpress II One Step Cloning Kit	Vazyme	Cat# C112-01
FastPure Plasmid Mini Kit	Vazyme	Cat# DC201-01
Cell Total RNA Isolation Kit	Foregene	Cat# RE-03113
PrimeScript™ RT reagent Kit (Perfect Real Time)	Takara	Cat# RR037Q
Rapid Fungi Genomic DNA Isolation Kit	Sangon	Cat# B518229
ChamQ Universal SYBR qPCR Master Mix	Vazyme	Cat# Q711-02/03
Luminol	Aladdin Scientific	Cat# L105655
Peroxidase	Solarbio	Cat# P8020
*N*,*N*’,*N*,*N*”‘,*N*”“,*N*”“‘-hexaacetyl chitohexaose	Solarbio	Cat# SN8990
Chitin	Solarbio	Cat# C8181
X-α-gal Solution	Coolaber	Cat# SL0940
Aureobasidin A	Takara	Cat# 630466
MG132	Sigma-Aldrich	Cat# C2211
Immun-Blot® PVDF	Bio-Rad	Cat# 1620177
Super ECL Detection Reagent	Yeasen	Cat# 36208ES60
Protease Inhibitor Cocktail	Selleck Chemicals	Cat# B14001
Phosphatase Inhibitor Cocktail	Selleck Chemicals	Cat# B15001
Isopropyl β-D-1-thiogalactopyranoside (IPTG)	Sigma-Aldrich	Cat# I5502
Anti-FLAG ® M2 Affinity gel	Sigma-Aldrich	Cat# A2220
GSTrap 4B chromatography column	Cytiva Life Sciences	Cat# 28401747
HisTrap HP chromatography column	Cytiva Life Sciences	Cat# 17524801
MBPSep Dextrin Agarose Resin 6FF	Yeasen	Cat# 20515ES08
PreScission Protease	Beyotime	Cat# P2302
Lambda Protein Phosphatase	Beyotime	Cat# P2316S
**Software**		
ImageJ		https://imagej.nih.gov/ij/
GroupPad		https://www.graphpad.com/features
DNAMAN		https://www.lynnon.com/
SnapGene Viewer		https://www.snapgene.com/snapgene-viewer
Bio-Rad		https://www.bio-rad.com/
**Other**		
Spectramax L Microplate reader	Molecular Devices	https://www.moleculardevices.com.cn/products/microplate-readers/luminescence-readers/spectramax-l-luminescence-reader
NanoDrop™ 2000	Thermo Fisher	https://www.thermofisher.cn/order/catalog/product/ND-2000
CFX Connect™	Bio-Rad	https://www.bio-rad.com/zh-cn/product/cfx-connect-real-time-pcr-detection-system?ID=LN5TFG15
Leica STELLARIS 8 STED	Leica	https://www.biomall.in/product/stellaris-8-sted-microscope-stellaris-8-sted
Criterion™ electrophoresis apparatus	Bio-Rad	https://www.bio-rad.com/zh-cn/sku/1656001-criterion-cell?ID=1656001
Touch Imager electronic tablet imaging device	e-BLOT	https://www.e-blot.com/touch-imager/
ÄKTA avant25	Cytiva Life Sciences	https://www.cytivalifesciences.com.cn/zh/cn/products/items/akta-avant-p-06264?psmenu=2

### Plant materials and growth conditions

*MAPK16*-OE plants and knockout mutants were generated by Wimi Biotechnology Co., Ltd. (Jiangsu, China) using the inbred line KN5585 as the recipient. The wild-type maize KN5585 used in this study was obtained from the segregation of transgenic lines. *Nicotiana benthamiana* was cultivated in a greenhouse with a 16/8-h day/night photoperiod, a temperature of 23 °C, and a relative humidity of 70% for ~30 days. The plants were then used for transient gene expression analyses. Maize seedlings were cultivated in a greenhouse with a 14/10-h day/night photoperiod, a temperature of 25 °C, and a relative humidity of 60% for ~20 days. The leaves were then inoculated with *F. verticillioides* XY-1 or subjected to a chitin-induced ROS burst assay. To investigate the effects of *MAPK16* in the B73 genetic context, *MAPK16*-OE plants and *mapk16* mutants were hybridized and backcrossed with B73 to introduce an overexpression cassette and a knockout mutation, respectively. Standard polymerase chain reaction (PCR) and kompetitive allele specific PCR were used to detect the overexpression cassette and single-base deletion, respectively. The primers used are listed in Dataset EV4.

To investigate the severity of ear and stalk rots in the field, maize was planted in 2023 and 2024 at Xishuangbanna (21°53′N, 100°59′E), Wenjiang (30°43′N, 103°52′E), and Chongzhou (30°33′N, 103°39′E) in China, with additional plantings at Wenjiang and Chongzhou in 2025. Seedlings were planted at each location in a randomized block design with three replicates in rows of 20 plants, with a row length of 3.5 m and a row spacing of 0.6 m. Approximately 60 days after planting, a hole with a diameter of 10 cm was dug near the roots, and 50 g of maize kernels colonized by *F. verticillioides or F. graminearum* were buried to induce stalk rot. Fourteen days after pollination, 200 μL of spore suspension (5 × 10^6^ spores/mL) was inoculated into the ears using the side-needle syringe method to induce ear rot. To investigate seed-borne *F. verticillioides* seed rot, asymptomatic seeds were collected from the vicinity of the infected sites on artificially inoculated ears. Ten seeds from each replicate were surface-sterilized and incubated on wet filter paper at 28 °C for three days in the dark, and three replicates were conducted. The degree of disease in each seed was artificially assigned using a five-point scale with five levels: 1, 3, 5, 7, and 9, where higher values indicated more severe symptoms.

### Microbial strains and culture conditions

*F. verticillioides* XY-1 and *F. graminearum* QN-1 were isolated from infected maize ears using a single-spore isolation method (Xiong et al, 2024). The corn sand was soaked in hot water (80 °C) for 2 h, after which the filtrate was autoclaved and used to cultivate XY-1 and QN-1. To facilitate QN-1 sporulation, QN-1 was transferred to sodium carboxymethylcellulose medium for further culture. Following 5–10 days of growth at 28 °C in the dark, the spore concentration reached a level of more than 5 × 10^6^ per milliliter, which could be used for inoculation. *Escherichia coli* (DH5α and BL21 (DE3)) and *Agrobacterium tumefaciens (GV3101)* were grown in LB or YEB liquid media with appropriate antibiotics at 37 and 28 °C, respectively.

### *F. verticillioides* resistance of detached leaves

The middle section of the third healthy leaf (approximately 6 cm) was selected for inoculation. The pipette tip was used to gently press and rotate the adaxial surface of the leaf to create four lesion points, to which 2 μL of spore droplets (5 × 10^6^ spores/mL) were applied. The inoculated leaves were floated in distilled water containing 1 mg/L 6-benzylaminopurine and stored in the dark. After ~2 days, the leaves were photographed, and the lesion size on each leaf was measured. To assess the biomass of XY-1, all leaves from each sample were combined for DNA isolation using a Rapid Fungi Genomic DNA Isolation Kit (Sangon, China).

### Colonization of spores on the surface of detached leaves

Undamaged leaves were floated on the spore solution and kept in the dark for 16 h to examine spore colonization on the leaf surface. The leaves were then treated with 70 °C ethanol to remove chlorophyll, followed by staining with an acid fuchsin solution (10 mL of phenol, 10 mL of glycerin, 10 mL of lactic acid, and 3 mg of acidic fuchsin) for 48 h. After rinsing twice with clean water, the leaves were observed under a laser confocal microscope (Leica STELLARIS STED; laser line, 555 nm; emission wavelength, 570‒660 nm). Six regions were uniformly selected from the center of each leaf, and images were acquired using a ×20 objective. The total number of spores in the six fields of view was used to calculate leaf spore colonization.

### Detection of chitin-induced ROS

Reactive oxygen species (ROS) levels were measured after the elicitor treatment. Briefly, a 3 mm diameter leaf disc was placed in the wells of a 96-well white microplate. Leaf discs were rinsed overnight with distilled water at 25 °C. After removing the distilled water, 200 μL of fresh distilled water was added for a second wash. Once this was removed, 200 μL of a luminol mixture (200 μM luminol and 20 μg/mL peroxidase) was added, and the mixture was incubated at 30 °C for 30 min. Subsequently, 10 μL of *N*,*N*′,*N*,*N*′′′,*N*′′′′,*N*′′′′′-hexaacetyl chitohexaose (SN8990; Solarbio, China) at 100 μg/mL was quickly added to each well. Chitin (C8181; Solarbio, China) was added at a concentration of 1 mg/mL to induce ROS production in the tobacco leaves. The signal acquisition took place at 30 °C using a Spectramax L microplate reader over 1 h, with measurements taken every minute. Eight replicates were used for each treatment. The ROS levels at each time point are presented as relative luminescence units (RLU).

### Alignment analysis of homologous proteins

The amino acid sequence of MPEL1 was used to search for orthologous proteins in *Arabidopsis thaliana*, *Schizosaccharomyces pombe, Oryza sativa, Solanum lycopersicum, and Homo sapiens* using the RefSeq protein database and BLASTP in the NCBI database. The top-ranked proteins containing only one RING/U-box domain were selected as the ortholog of interest. A phylogenetic tree was constructed using MEGA7.

### Protein expression and purification from *E. coli*

As described in our previous study (Feng et al, 2025a), *E. coli* strain BL21(DE3) harboring the recombinant plasmids was cultured at 37 °C until OD_600_ reached approximately 0.6, then induced with 0.5 mM isopropyl β-D-1-thiogalactopyranoside and incubated at 16 °C for about 16 h to express the proteins. Recombinant proteins were released from *E. coli* cells via ultrasonication. GST-tagged recombinant proteins were extracted in 1× phosphate-buffered saline (PBS) (10 mM Na_2_HPO_4_, 2 mM NaH_2_PO_4_, 135 mM NaCl, 4.7 mM KCl, and pH7.4). His-tagged recombinant proteins were extracted in a binding buffer (20 mM NaH_2_PO_4_, 0.5 M NaCl, 20 mM imidazole, and pH 7.4). GST- and His-tagged recombinant proteins were purified using GSTrap 4 B (28401747; Cytiva, Sweden) and HisTrap HP (17524801; Cytiva, Sweden), respectively, using the AKTA avant25 protein chromatography system (Cytiva, Sweden). GST-tagged recombinant proteins were eluted using GST elution buffer (50 mM Tris-HCl, pH 7.4, 20 mM reduced L-glutathione (G8180; Solarbio, China)). His-tagged recombinant proteins were eluted using His elution buffer (20 mM NaH_2_PO_4_, 0.5 M NaCl, 500 mM imidazole, and pH 7.4). MBP-tagged recombinant proteins were extracted in a column buffer (20 mM Tris-HCl, pH 7.4, 200 mM NaCl, 1 mM EDTA, and 1 mM DTT). MBPSep dextrin agarose resin 6FF (20515ES08; Yeasen, China) was then incubated with the protein solution for approximately 2 h and rinsed four times with the column buffer. Finally, the resin-bound proteins were eluted using MBP elution buffer (20 mM Tris-HCl, pH 7.4, 200 mM NaCl, 1 mM EDTA, 1 mM DTT, and 20 mM maltose (CM7181; Coolaber, China)).

### Protein–protein interaction assays

The full-length, N-, and C-terminal regions of *MAPK16* were individually cloned into the pGBKT7 vector and fused to the *Gal4* DNA-binding domain (BD). The primers used for cloning are listed in Dataset EV4. Before detecting protein–protein interactions, the three vectors were tested for their transcriptional activation properties in yeast cells. A cDNA library was constructed in yeast cells by Shanghai OE Biotech Co., Ltd., China. Yeast two-hybrid library screening was performed according to the Yeast Protocol Handbook (Clontech, USA). The screened bait genes were cloned into the pGADT7 vector for fusion with the *Gal4* transcriptional activation domain (AD). Yeast cells harboring pGBKT7 and pGADT7 vectors were serially diluted and cultured on non-selective (SD/-Trp-Leu) and selective (SD/-Trp-Leu-His-Ade, containing 40 μg/mL x-α-gal) media. 200 ng/mL aureobasidin A (630466; TAKARA, Japan) was added to the medium when required. The combination of pGBKT7-53 and pGADT7-T served as a positive (+), whereas pGBKT7-Lam and pGADT7-T served as negative (‒) controls.

For pull-down assays, Maltose-binding proteins (MBP) or Flag-tagged proteins were incubated with GST-tagged proteins, along with MBPSep Dextrin Agarose Resin 6FF (20515ES08; Yeasen, China) or sigma-anti-FLAG M2 affinity gel (A2220; Sigma, USA), in binding buffer (50 mM Tris-HCl, pH 7.4, 120 mM NaCl, 1 mM EDTA, 10% glycerol, and 0.5% Triton X-100) at 4 °C for about 2 h. M2 affinity gel or agarose resin was then rinsed three times with binding buffer. A 200 μg/mL Flag peptide solution (P9801; Beyotime, China) was used to elute proteins bound to the M2 affinity gel. In contrast, the MBP elution buffer was used to elute proteins bound to the MBPSep dextrin agarose resin. The eluted proteins were subsequently immunoblotted with monoclonal anti-FLAG M2 antibody (F3165; Sigma, USA), anti-GST antibody (30901ES50; Yeasen, China), and MBP-Tag Polyclonal antibody (15089-1-AP; Proteintech, USA).

Bimolecular fluorescence complementation assays were performed as previously described (Feng et al, 2022). The protein-coding sequences of *MPEL1*, *JAZ20*, *ZIM1*, and *MAPK16* were individually cloned into either pXYc104 or pXYn106 vectors and fused to the C-terminus (cYFP) or N-terminus (nYFP) of YFP. Subsequently, the specified plasmid combinations were cotransformed into *Agrobacterium* cells and transiently expressed in *N. benthamiana* leaves. The fluorescence signals were examined using a confocal laser microscope (LSM800; Carl Zeiss, Germany). The primers used for cloning are listed in Dataset EV4.

For the CoIP assay, the protein-coding sequences of *MPEL1* and *JAZ20* were individually cloned into the pJIBIA163-1300 vector via fusion with green fluorescent protein (*GFP*) at their C-termini. *MAPK16* was cloned into the pLANT-cFlag vector with a Flag tag at the C-terminus. Subsequently, the specified plasmid combinations were cotransformed into *Agrobacterium* cells and transiently expressed in tobacco leaves for approximately 48 h. Total protein was isolated using an IP lysate (50 mM Tris-HCl, pH 7.4, 120 mM NaCl, 10 mM EDTA, 5 mM DTT, 0.5% Triton X-100, and a protease inhibitor cocktail [B14001; Selleck, USA]). The total protein was then incubated with anti-FLAG M2 affinity gel (A2220; Sigma, USA) at 4 °C for 2 h, followed by two rinses and competitive elution using a 200 μg/mL Flag peptide solution (P9801; Beyotime, China). The eluted protein was subsequently used for immunoblotting with a monoclonal anti-FLAG M2 antibody (F3165; Sigma, USA).

### In vitro phosphorylation

Recombinant proteins were incubated in kinase buffer (50 mM Tris-HCl, pH 7.4, 1 mM DTT, 10 mM MgCl_2_, along with a protease inhibitor cocktail and a phosphatase inhibitor cocktail [B15001; Selleck, USA]; ATP is typically included at a concentration of 5 mM, unless otherwise specified) at 30 °C for 30 min. The proteins were then subjected to immunoblotting using anti-phospho(serine/threonine) antibody (PP2551; ECM, Italy) and anti-GST antibody (30901ES50; Yeasen, China).

To investigate whether MAPK16 undergoes autophosphorylation, GST-MAPK16 and GST-MAPK16^AF^ (in which the TDY motif was mutated to ADF) recombinant proteins were dephosphorylated using Lambda Protein Phosphatase (P2316S; Beyotime, China) in a water bath at 28 °C for 30 min followed by in vitro phosphorylation incubation. Immunoblotting was performed using anti-phospho(serine/threonine) antibody (PP2551; ECM, Italy), anti-GST antibody (30901ES50; Yeasen, China), and customized anti-pMAPK16(TDY^p^) antibody (ABclone, China). Recombinant proteins that were not treated with Lambda Protein Phosphatase served as controls.

### In vitro SUMOylation

Recombinant proteins were incubated in a reaction buffer (20 mM HEPES, pH 7.4, 10 mM MgCl_2_, and 5 mM ATP) at 30 °C for 1 h. Proteins were then immunoblotted using an anti-maltose-binding protein polyclonal antibody (15089-1-AP; Proteintech, USA) or an anti-GST antibody (30901ES50; Yeasen, China). The plasmids for SUMO-activating enzyme (SUMO E1), SUMO-conjugating enzyme (SUMO E2), SUMO1^AA^, and SUMO1^GG^ were provided by Professor Jingbo Jin from the Institute of Botany, Chinese Academy of Sciences, and Professor Chengwei Yang from South China Normal University (Qu et al, 2020).

### In vivo SUMOylation

The recombinant proteins were transiently expressed in tobacco leaves for approximately 36 h. Total protein was isolated using an IP lysate (50 mM Tris-HCl, pH 7.4, 120 mM NaCl, 10 mM EDTA, 5 mM DTT, 0.5% Triton X-100, and a protease inhibitor cocktail [B14001; Selleck, USA]). The total protein was then incubated with anti-FLAG M2 affinity gel (A2220; Sigma, USA) at 4 °C for 2 h, followed by two rinses and competitive elution using a 200 μg/mL Flag peptide solution (P9801; Beyotime, China). The eluted protein was subsequently used for immunoblotting with Anti-GFP (D110008; Sangon, China), anti-Flag (F3165; Sigma, USA), and anti-Actin (D110007; Sangon, China) antibodies. Anti-SUMO1 antibody (ab5316; Abcam, UK) was used to detect SUMOylation in *mpel1* mutants.

### In vitro ubiquitination

Recombinant proteins were incubated in reaction buffer (20 mM HEPES, pH7.4, 10 mM MgCl_2_, 20 μM ZnCl_2_ and 5 mM ATP) at 30 °C for 1–2 h. The proteins were then subjected to immunoblotting using anti-ubiquitin antibody (10201-2-AP; Proteintech, USA) and anti-GST antibody (30901ES50; Yeasen, China). The GST tag was excised from GST-MPEL1 using PreScission Protease (P2302; Beyotime, China) before this experiment. The Ubiquitin-activating enzyme (Ub E1), ubiquitin-conjugating enzyme (Ub E2), and ubiquitin were cloned from maize, and our team constructed the corresponding plasmids. An anti-ubiquitin antibody (10201-2-AP; Proteintech, USA) was used to detect ubiquitination in *mpel1* mutants.

### In vitro protein degradation

To analyze the degradation of various site-mutated JAZ20 proteins, GST-JAZ20, GST-JAZ20^T12AS13^, and GST-JAZ20^T12DS13D^ were incubated with total protein from KN5585 in reaction buffer (50 mM Tris-HCl, pH 7.4, 120 mM NaCl, 10% glycerol) at 30 °C for varying durations. Similarly, GST-JAZ20 was incubated with total protein from *MAPK16* overexpressing plants, knockout mutants, and wild-type plants to assess the effect of MAPK16 on the degradation of JAZ20. To evaluate the impact of SUMOylation on the stability of MAPK16, GST-MAPK16 was partially SUMOylated by MPEL1 in vitro for two hours and recovered using GSTSep glutathione agarose resin (20507ES10; Yeasen, China). The recovered proteins were then incubated with total protein from KN5585 in reaction buffer at 30 °C for varying durations. The ImageJ software was used to quantify the signal intensity of the bands on the western blot.

### Protein fractionation assay

The recombinant proteins were transiently expressed in tobacco leaves for approximately 48 h. Before isolating the cytoplasm and nucleus, fluorescence microscopy was performed to confirm regular protein expression. Tobacco leaves were ground in liquid nitrogen, taking care not to grind them too finely to avoid damaging their nuclei. Approximately 3 g of the sample powder was combined with 4 mL of extraction buffer A (250 mM sucrose, 25 mM Tris-HCl, pH 7.4, 25% glycerol, 20 mM KCl, 2.5 mM MgCl_2_, 2 mM EDTA, and protease inhibitor cocktail [B14001; Selleck, USA]). The mixtures were then filtered and collected sequentially using 40-mesh and 100-mesh sieves. A 100 μL aliquot of the filtrate was reserved for detecting the total expression of the protein of interest. The filtrate was centrifuged at 20,000×*g* for 8 min at 4 °C, and the supernatant was used to measure the expression level of the protein of interest in the cytoplasm. Subsequently, extraction buffer B (250 mM sucrose, 10 mM Tris-HCl, pH 8.0, 5 mM β-mercaptoethanol, 10 mM MgCl_2_, 1% Triton X-100) was added to the pellet, which was washed three times. Finally, the pellet was lysed in 800 μL 1× SDS loading buffer and used to assess the expression level of the target protein in the nucleus. A 5× SDS loading buffer was added to the total and cytoplasmic fractions before SDS-PAGE.

### Treatment with MG132 in tobacco leaves

JAZ20^T12AS13A^-GFP, JAZ20^T12DS13D^-GFP, and GFP were transiently expressed in the tobacco leaves for 36 h. The treatment group was subsequently injected with 20 μM of MG132, while the control group received the same dose of DMSO (solvent of MG132). Three hours post-injection, fluorescence was observed, and samples were collected for western blotting. Each protein was expressed in six tobacco leaves, with the left and right halves of each leaf treated with MG132 and DMSO, respectively. Finally, six halves of the same protein sample corresponding to each treatment were pooled for total protein extraction.

### Proteomic mass spectrometry analysis

Comparative proteomic analysis was conducted using *MAPK16*-OE plants and knockout mutants, with sample processing methods identical to those employed in the investigation of *F. verticillioides* resistance in detached leaves. After 10 h, the leaves were subjected to comparative proteomic analysis. Proteomic mass spectrometry was conducted by Bioprofile Technology Co., Ltd. (Shanghai, China). Proteins were extracted from the leaves using SDT lysis buffer (4% SDS, 100 mM DTT, and 100 mM Tris-HCl, pH 8.0). The samples were boiled for 3 min and ultrasonicated. Undissolved cellular debris was removed by centrifugation at 16,000×*g* for 15 min, and the supernatant was collected and quantified using a BCA Protein Assay Kit (P0012; BeyoTime, China). Protein digestion was performed using the filter-aided sample preparation (FASP) method described by Wisniewski et al* (*Wiśniewski et al, 2009*)*. Briefly, detergent, DTT, and iodoacetamide (IAA) in UA buffer were added to block cysteine reduction. The protein suspension was then digested with trypsin (Promega) at a ratio of 50:1 overnight at 37 °C. The resulting peptide mixtures were collected by centrifugation at 16,000×*g* for 15 min and desalted using a C18 StageTip before further LC-MS analysis. The concentrations of the re-dissolved peptides were determined using OD_280_ with a Nanodrop One device (Thermo Fisher Scientific, USA).

LC-MS/MS analyses were conducted using an Orbitrap Astral mass spectrometer coupled to a Vanquish Neo UHPLC system (Thermo Fisher Scientific, USA). Peptides from each sample were loaded onto a 50 cm Low-Load µPAC™ Neo HPLC Column (Thermo Fisher Scientific, USA) at a flow rate of 2.2 μL/min. The reverse-phase HPLC mobile phase consisted of 0.1% formic acid in water (A) and 0.1% formic acid in 80% acetonitrile (B). Peptides were eluted over 8 min with a linear gradient of buffer B at 1.25 μL/min, set as follows: 0–0.1 min, 4% to 6% buffer B; 0.1–1.1 min, 6% to 12% buffer B; 1.1–4.3 min, 12% to 25% buffer B; 4.3–6.1 min, 25% to 45% buffer B; 6.1–6.5 min, 45% to 99% buffer B; and 6.5–8 min, maintained at 99% buffer B. The eluted peptides were analyzed using an Orbitrap Astral mass spectrometer. The data-independent acquisition (DIA) method included a survey scan from 380–980 m/z at a resolution of 240,000 with an AGC target of 500% and a 5 ms injection time. DIA MS/MS scans were acquired at 150–2000 m/z with a 2 m/z isolation window, an AGC target of 500%, and a 3 ms injection time. The normalized collision energy was set to 25, and the cycle time was 0.6 s. The spectra of the full MS and DIA scans were recorded for profile and centroid types, respectively.

DIA MS data were analyzed using DIA-NN version 1.8.1. MS data were searched against UniProtKB *Zea mays* (maize)[4577]207454 2023 12 11 fasta sequence using trypsin as the digestion enzyme. A maximum of one missed cleavage was allowed, with a mass tolerance of 10 ppm for both precursor and fragment ions. Carbamidomethylation of cysteine was defined as a fixed modification, whereas acetylation of the protein N-terminus and oxidation of methionine were defined as variable modifications. The maximum number of variable modifications is limited to one. Peptide lengths ranged from 7 to 30 amino acids, and peptide charges ranged from 1 to 4. The fragment ion m/z range was defined as 150–2000. The database search results were filtered and exported with a false discovery rate (FDR) of less than 1% at both the peptide-spectrum-matched and protein levels. Bioinformatics analyses were performed using Microsoft Excel and R statistical software. Sequence annotation was facilitated by extracting information from UniProtKB/Swiss-Prot, the Kyoto Encyclopedia of Genes and Genomes (KEGG) (Kanehisa et al, 2011), and Gene Ontology (GO) (Ashburner et al, 2000). GO and KEGG enrichment analyses were conducted using Fisher’s exact test with FDR correction for multiple tests. Enriched GO and KEGG pathways were considered nominally statistically significant if the Fisher’s exact test *p*-value was less than 0.01.

### Protein accession number

Sequence data in this study can be found in the Gramene database under the following accession numbers: *MAPK16* (GRMZM2G017351_T6), *MPEL1* (Zm00001eb143350), *JAZ20* (Zm00001d022139_T1), *ZIM1* (Zm00001eb312170), *ZIM36* (Zm00001eb226740), *ZIM18* (Zm00001eb048780), *ZIM2* (Zm00001eb215440), *ZIM7* (Zm00001eb220310), *ZIM20* (Zm00001eb273020), *Ub E1* (Zm00001eb195000), *UBC2* (Zm00001eb300890), *UBC8* (Zm00001eb157900), *UBC11* (Zm00001eb278070), *UBC27* (Zm00001eb363890), *AtUBC9* (AT4G27960), *Ubi1* (Zm00001eb313000), *AtSTUBL1* (AT5G48655), *PR4* (Zm00001eb299370), *PR5* (Zm00001eb032600), *PR6* (Zm00001eb014010), *PR26* (Zm00001eb169820), *CHN22* (Zm00001eb002620), *Slx8* (P87176), *Rfp2* (Q9UT72), *LOX1* (Zm00001eb144960), *LOX2* (Zm00001eb144930), *LOX3* (Zm00001eb054040), *LOX4* (Zm00001eb054050).

## Supplementary information

Appendix

Table EV1

Table EV2

Peer Review File

Dataset EV1

Dataset EV2

Dataset EV3

Dataset EV4

Source data Fig. 1

Source data Fig. 2

Source data Fig. 3

Source data Fig. 4

Source data Fig. 5

Source data Fig. 6

Source data Fig. 7

Source data Fig. 8

Expanded View Figures

## Data Availability

The full proteome data can be accessed through the China National Center for Bioinformation (CNCB) (BioProject ID: PRJCA032969) (https://ngdc.cncb.ac.cn/bioproject/browse/PRJCA032969); Any additional information required to reanalyze the data reported in this work are available in the main text or supplementary materials. The source data of this paper are collected in the following database record: biostudies:S-SCDT-10_1038-S44318-026-00811-2.
