# Supplementary material for: A new plant SUMO ligase, MPEL1, synergizes with MAPK16 to regulate resistance against Fusarium pathogens
Source: EMBO J. 2026 May 18;45(13):4670–93. doi: 10.1038/s44318-026-00811-2 (PMC13324008; doi:10.1038/s44318-026-00811-2)
Supplement: Supplementary file 1 — Appendix [file 44318_2026_811_MOESM1_ESM.pdf]

Appendix for:

A new plant SUMO ligase, MPEL1, synergizes with MAPK16 to regulate resistance against Fusarium pathogens

Table of Contents

Appendix Figure S1. Appendix Figure S1. Transcriptional response of nine defense related genes to *F. verticillioides* in OE and KO plants……………………2

Appendix Figure S2. Subcellular localization of MPEL1 and MAPK16…………2

Appendix Figure S3. The effects of MAPK16 and MPEL1 on chitin-induced reactive oxygen species burst……………………………………………………...3

Appendix Figure S4. MPEL1 is a homolog of SUMO-targeted ubiquitin ligases…4

Appendix Figure S5. MPEL1 interacts with covalent SUMO1-E2 conjugate…….5

Appendix Figure S6. ATG8b is a SUMOylation substrate of MAPEL1…………..5

Appendix Figure S7. *In vitro* SUMOylation modification assays for GST-JAZ20 with or without MAPK16 mediation………………………………………….…6

Appendix Figure S8. The effect of MG132 on the accumulation of site-mutated JAZ20 in tobacco leaves………………………………………………………….….6

Appendix Figure S9. Effects of *JAZ20* loss-of-function on chitin-triggered ROS burst and *PR* gene expression……………………………………………………7

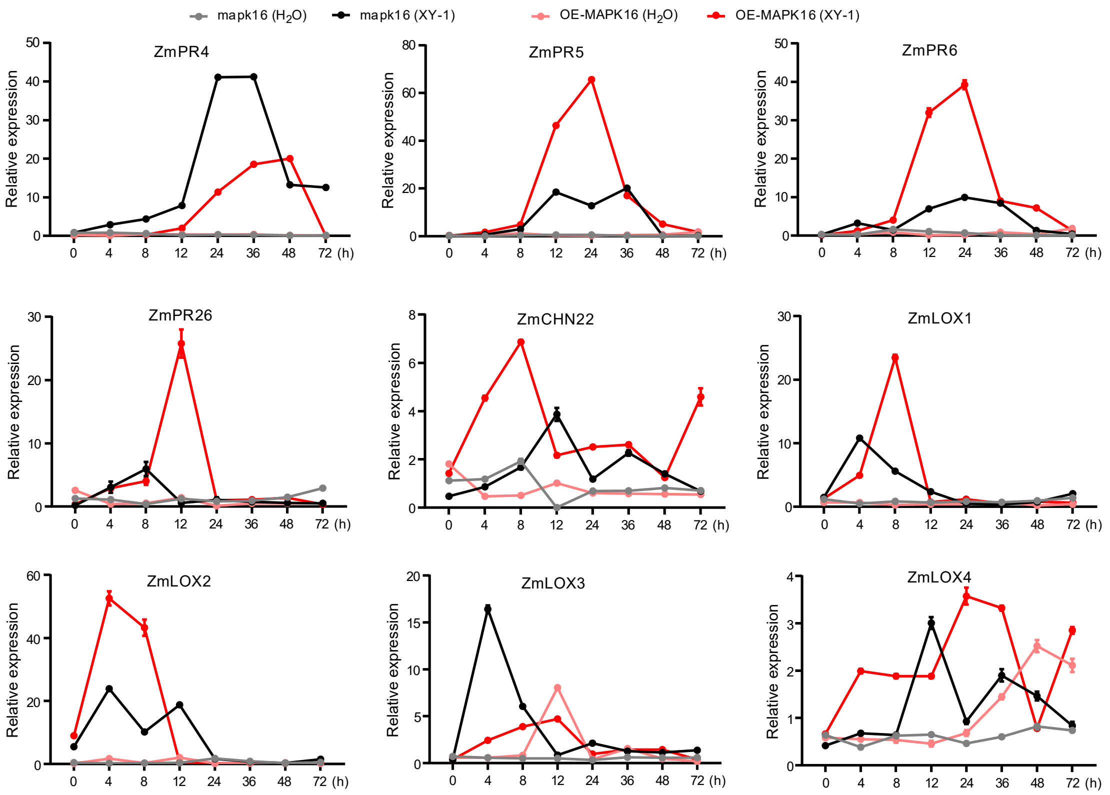

Appendix Figure S1. Transcriptional response of nine defense related genes to *F. verticillioides* in OE and KO plants. After inoculating leaves from 4-leaf-stage plants with spore suspension or sterile water, the leaves were covered with plastic wrap to maintain humidity. Samples were collected at designated time points for RNA extraction. *Ubi* and *eF1α* were used as internal reference genes. Data represent mean ± SEM (n = 3).

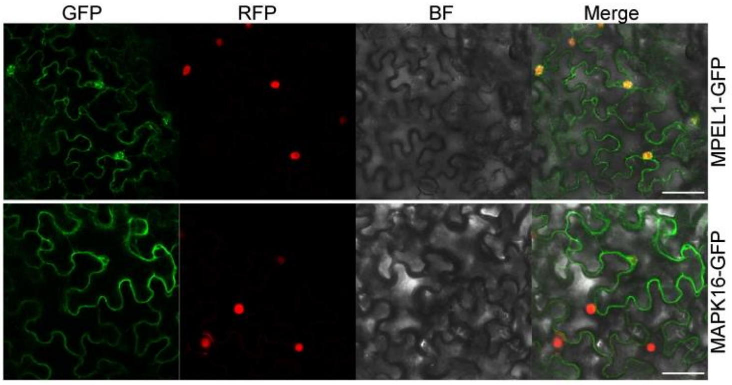

Appendix Figure S2. Subcellular localization of MPEL1 and MAPK16.

Recombinant proteins were transiently expressed in tobacco leaves. RFP represents a nuclear localization marker. Scale bar = 50 μm.

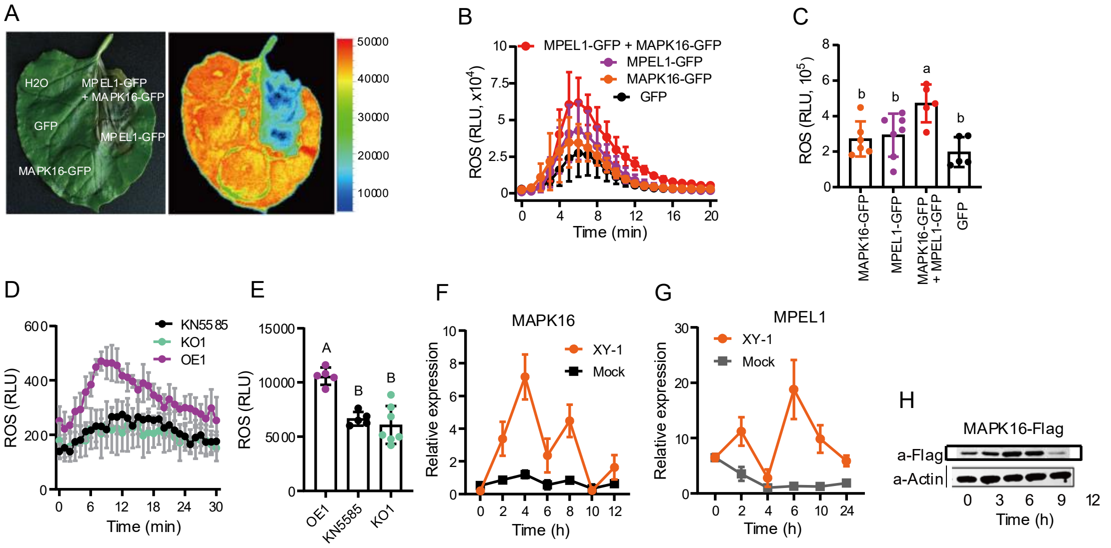

Appendix Figure S3. The effects of MAPK16 and MPEL1 on chitin-induced reactive oxygen species burst. (A) *MPEL1*, *MAPK16* were overexpressed either individually or in combination in tobacco leaves, and the presence of necrotic spots was assessed after four days. **(B–C)** The chitin-induced ROS burst was investigated approximately 36 hours after gene expression. Data represent mean ± SD. **(D–E)** Hexa-N-acetylchitohexaose induced ROS burst was detected on detached maize leaves (*MAPK16* overexpression, knockout mutant, and the wildtype) using a luminol-horseradish peroxidase method. Data represent mean ± SD. Statistical analysis was performed with one-way ANOVA followed by Tukey's multiple comparisons test. The letters displayed above the bars in the histogram indicate statistically significant differences between the two groups (lowercase: $p < 0.05$; uppercase: $p < 0.01$). **(F–G)** Transcriptional responses of *MAPK16* and *MPEL1* to *F. verticillioides* XY-1 invasion. After inoculating leaves from 4-leaf-stage plants with spore suspension or sterile water (Mock), the leaves were covered with plastic wrap to maintain humidity. Samples were collected at designated time points for RNA extraction. *Ubi* and *eF1α* were used as internal reference genes. Data represent mean ± SEM (n = 3). **(H)** Responses of *MAPK16* to *F. verticillioides* XY-1 infection at protein level using overexpressing plants. Data are representative of four independent experiments with similar outcomes.

A

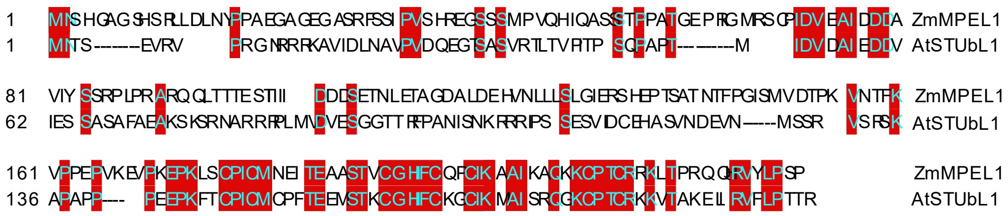

B

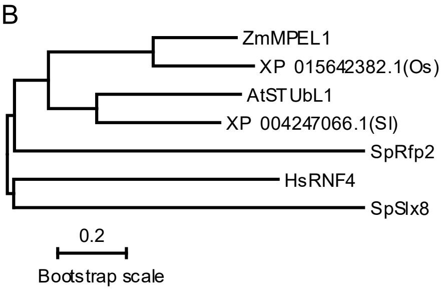

C

Results for predictive SIM [PSmax = 38.183 | Cut-off = 0.269]			
Protein ID	Position site	Sequence	Predictive score
ZmMPEL1	AA 12–15	LLDL	0.571
	AA 101–104	ITII	0.337
	AA 102–105	TIII	0.279
AtSTUbL1	AA 18–21	VIDL	38.183
Rfp2	AA 19–23	VIDLT	38.183
	AA 36–39	VILL	0.413
	AA 38–42	LLDLT	0.571
Slx8	AA 155–159	MIDLT	0.707
	AA 207–210	VICL	1.193
	AA 255–258	VICL	1.193

Appendix Figure S4. MPEL1 is a homolog of SUMO-targeted ubiquitin ligases. (A) MPEL1 has the highest similarity with AtSTUbL1 in Arabidopsis. **(B)** The phylogenetic tree illustrating the genetic relationships among seven orthologous proteins. The evolutionary history was inferred using the Neighbor-Joining method. The optimal tree with the sum of branch length = 4.70614202 is shown. The tree is drawn to scale, with branch lengths in the same units as those of the evolutionary distances used to infer the phylogenetic tree. The evolutionary distances were computed using the Poisson correction method and are in the units of the number of amino acid substitutions per site. Evolutionary analyses were conducted in MEGA7. **(C)** Predicted SUMO-interacting domain scores for ZmMPEL1, AtSTUbL1, Rfp2, and Slx8. (http://www.jassa.fr/index.php?m=jassa).

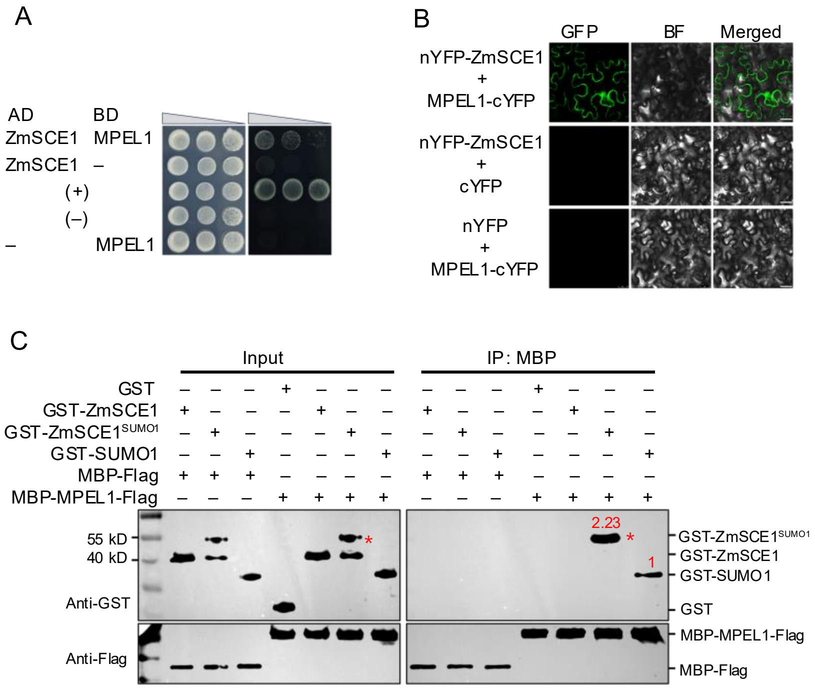

Appendix Figure S5. MPEL1 interacts with covalent SUMO1-E2 conjugate. (A) Yeast two-hybrid analysis of the interaction between MPEL1 and ZmSCE1 (a SUMO E2). **(B)** Bimolecular fluorescence complementation assays for protein-protein interactions. Scale bar = 50 μm. **(C)** Protein pull-down analyzing MPEL1 interaction with SUMO1, ZmSCE1, and pre-covalently linked SUMO1-ZmSCE1 conjugate. GST-ZmSCE1$^{-/SUMO1}$ indicates that ZmSCE1 is covalently coupled with SUMO1, resulting in both free ZmSCE1 and SUMO1-modified ZmSCE1. Red asterisk indicates ZmSCE1 covalently coupled to SUMO1. The values above the GST-ZmSCE1$^{-/SUMO1}$ and GST-SUMO1 bands in the IP results represent the normalized signal intensities. Normalization was performed using the signal intensities of GST-ZmSCE1$^{-/SUMO1}$ and GST-SUMO1 bands in the Input, as well as the MBP-MPEL1-Flag signal in the IP.

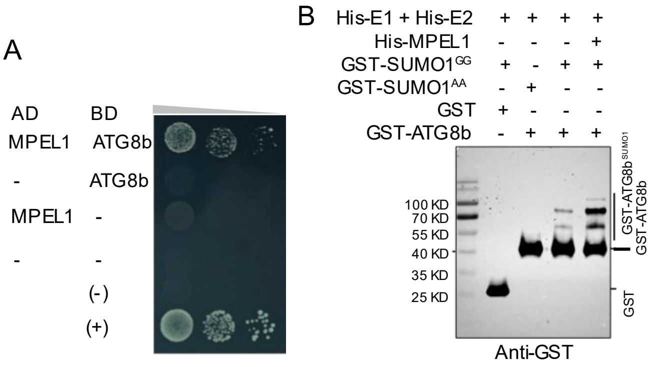

Appendix Figure S6. ATG8b is a SUMOylation substrate of MAPEL1. (A) Yeast two-hybrid analysis of the interaction between MPEL1 and ATG8b. The combination

of pGBKT7-53 and pGADT7-T served as a positive (+), whereas pGBKT7-Lam and pGADT7-T served as negative (–) controls. **(B)** *In vitro* SUMOylation assay using purified proteins. SUMO1^{AA} is a loss-of-function mutant of SUMO1 (namely SUMO1^{GG}) that cannot covalently conjugate to substrates.

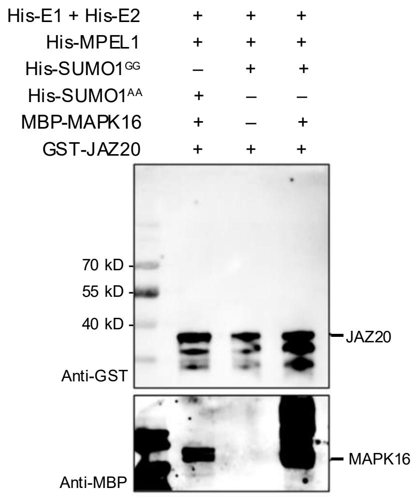

Appendix Figure S7. ***In vitro*** **SUMOylation modification assays for GST-JAZ20 with or without MAPK16 mediation.**

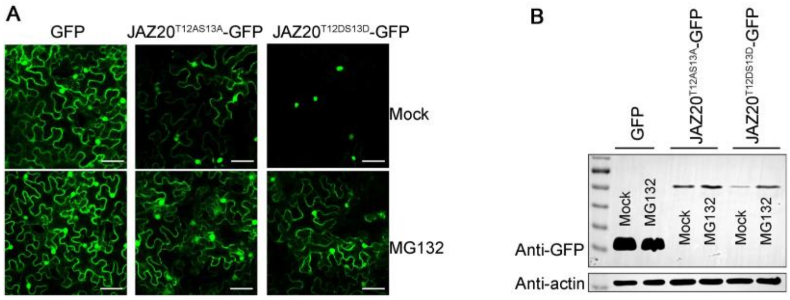

Appendix Figure S8. The effect of MG132 on the accumulation of site-mutated JAZ20 in tobacco leaves. (A) Laser confocal microscope was used to evaluate the responses of site-mutated JAZ20-GFP to MG132 treatment. Scale bar = 50 μm. **(B)** Western blot was used to detect the changes in protein abundance of site-mutated JAZ20-GFP after MG132 treatment.

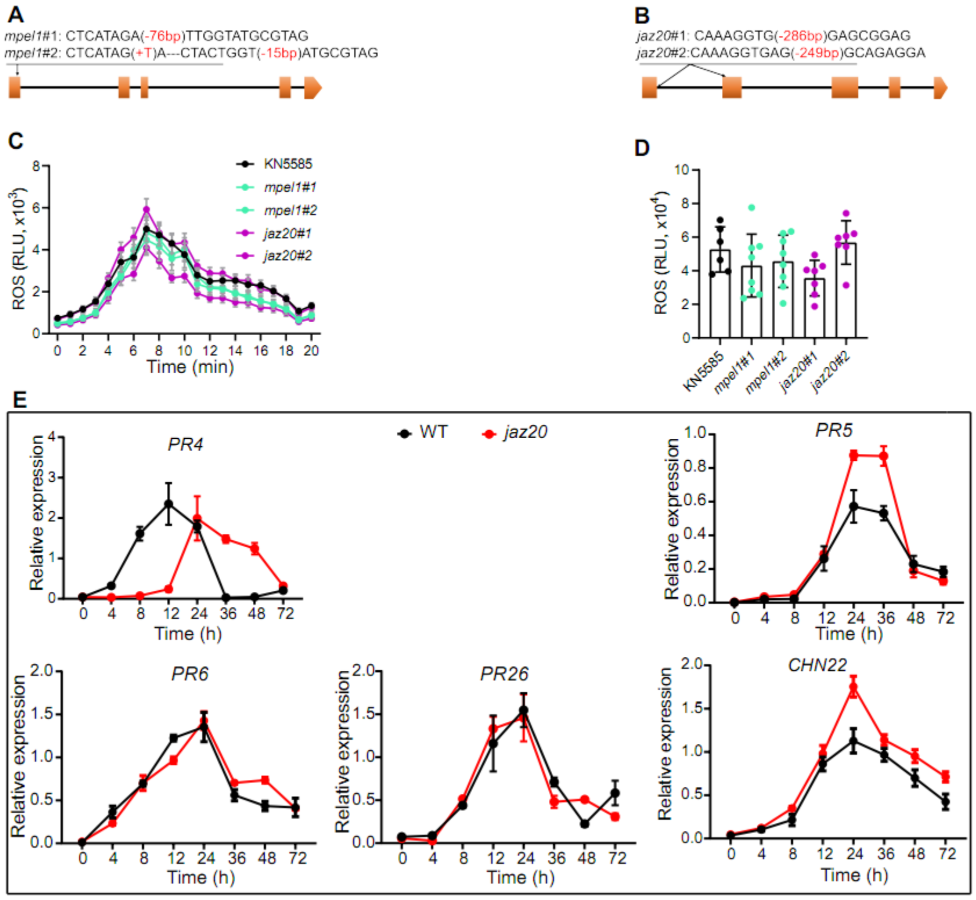

Appendix Figure S9. Effects of *JAZ20* loss-of-function on chitin-triggered ROS burst and *PR* gene expression. (A–B) Schematic diagrams illustrating the gene structure of *MPEL1* and JAZ20, and with the editing types for the two knockout lines of each gene. **(C–D)** Hexa-N-acetylchitohexaose induced ROS burst was detected on detached maize leaves using a luminol-horseradish peroxidase method. Data represent mean ± SD, with one-way ANOVA followed by Dunnett's multiple comparisons test to ascertain the statistical significance of the observed differences between knockout mutants and the wild-type. **(E)** Transcriptional response of five defense marker genes to *F. verticillioides* in the wildtype and *jaz20* knockout mutants. After inoculating leaves from 4-leaf-stage plants with spore suspension or sterile water, the leaves were covered with plastic wrap to maintain humidity. Samples were collected at designated time points for RNA extraction. *Ubi* and *eF1α* were used as internal reference genes. Data represent mean ± SEM (n = 3).
