# Supplementary material for: A new plant SUMO ligase, MPEL1, synergizes with MAPK16 to regulate resistance against Fusarium pathogens
Source: EMBO J. 2026 May 18;45(13):4670–93. doi: 10.1038/s44318-026-00811-2 (PMC13324008; doi:10.1038/s44318-026-00811-2)
Supplement: Supplementary file 17 — Expanded View Figures [file 44318_2026_811_MOESM17_ESM.pdf]

Expanded View Figures

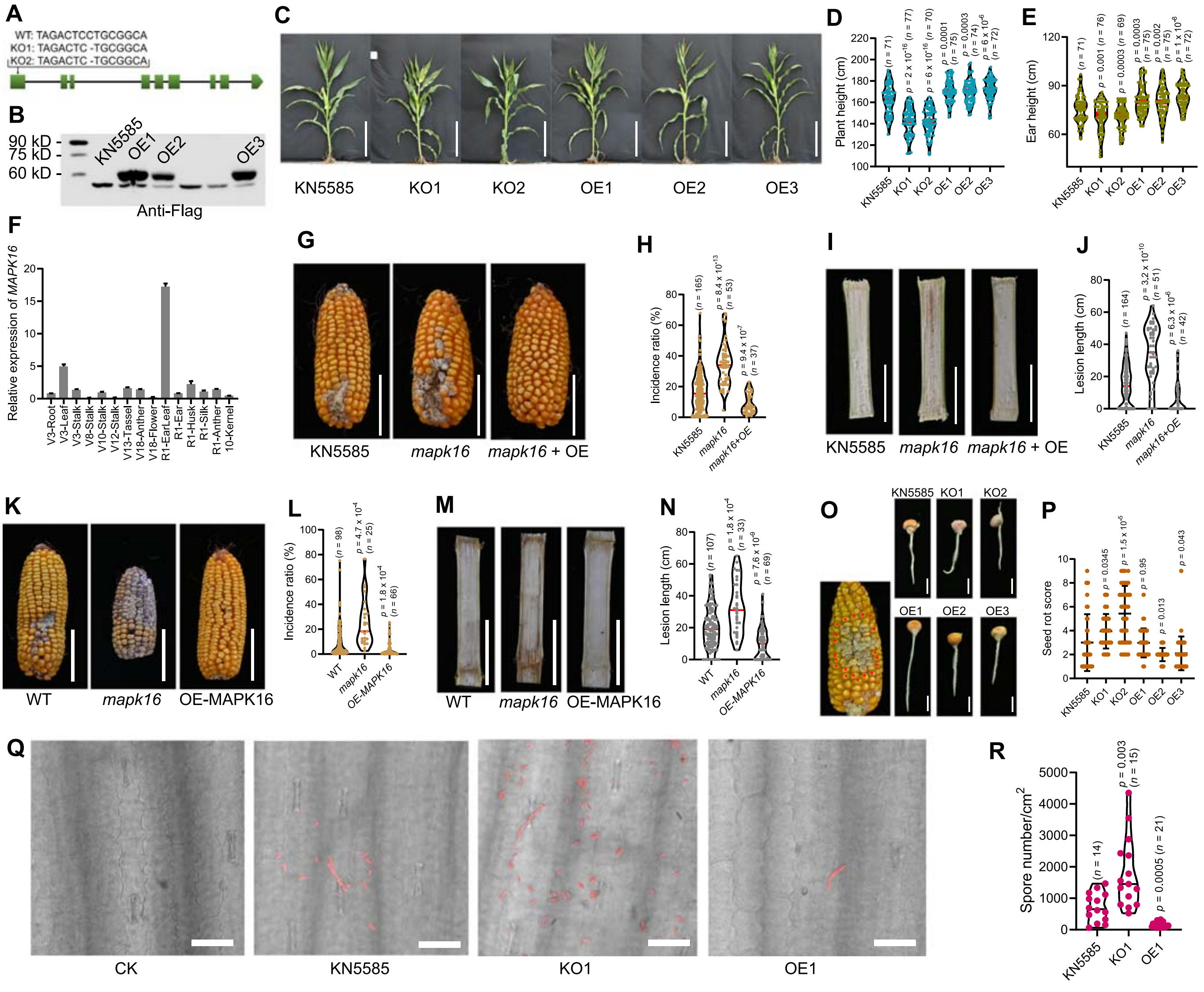

Figure EV1. *MAPK16* is involved in the regulation of *F. verticillioides* resistance and maize development.

(**A**) Schematic diagram of the gene structure of *MAPK16* and the editing types of the two knockout lines. (**B**) Protein expression levels in different transgenic overexpression lines. (**C–E**) Plant phenotype and statistic values of plant height and ear height. Scale bar = 50 cm. (**F**) Transcriptional level of *MAPK16* in various tissues. *GAPDH* and *eF1α* were used as internal reference genes. Data represent mean ± SEM ($n = 3$). (**G–N**) Phenotypes and corresponding statistical values of *F. verticillioides* caused maize ear rot and stalk rot. (**G–J**) The *mapk16 + OE* represents overexpression of *MAPK16* in the genetic context of the *mapk16* mutant. Scale bar = 5 cm. (**K–N**) *MAPK16* overexpression cassette and knockout mutation were introduced into the B73 genetic context by backcrossing. The *mapk16* mutants, *OE-MAPK16*, and wild-type plants were identified from the separation of offspring. Scale bar = 5 cm. (**O**, **P**) Seed rot was investigated using the seeds with no visible symptoms around the lesions, which are indicated with red asterisks, during seed germination. Scale bar = 1 cm. (**Q**, **R**) The colonization of spores on leaves. CK indicates that the detached leaves were exposed to *F. verticillioides* spores and immediately removed to a non-*F. verticillioides* conditions. Scale bar = 50 μm. Panels (**D**, **E**) represent three biological replicates; panels (**H**, **J**, **L**, **N**, **R**) show one representative result from three independent experiments with similar outcomes. Data in panels (**D**, **E**, **H**, **J**), **L**, **N**, **R**) are shown as violin plots, with the median indicated by a short line. Panel (**P**) shows one representative result from three independent experiments with similar outcomes. Data were presented as mean ± SD. Statistical analysis was performed via one-way ANOVA followed by Dunnett's multiple comparisons test.

 © The Author(s)

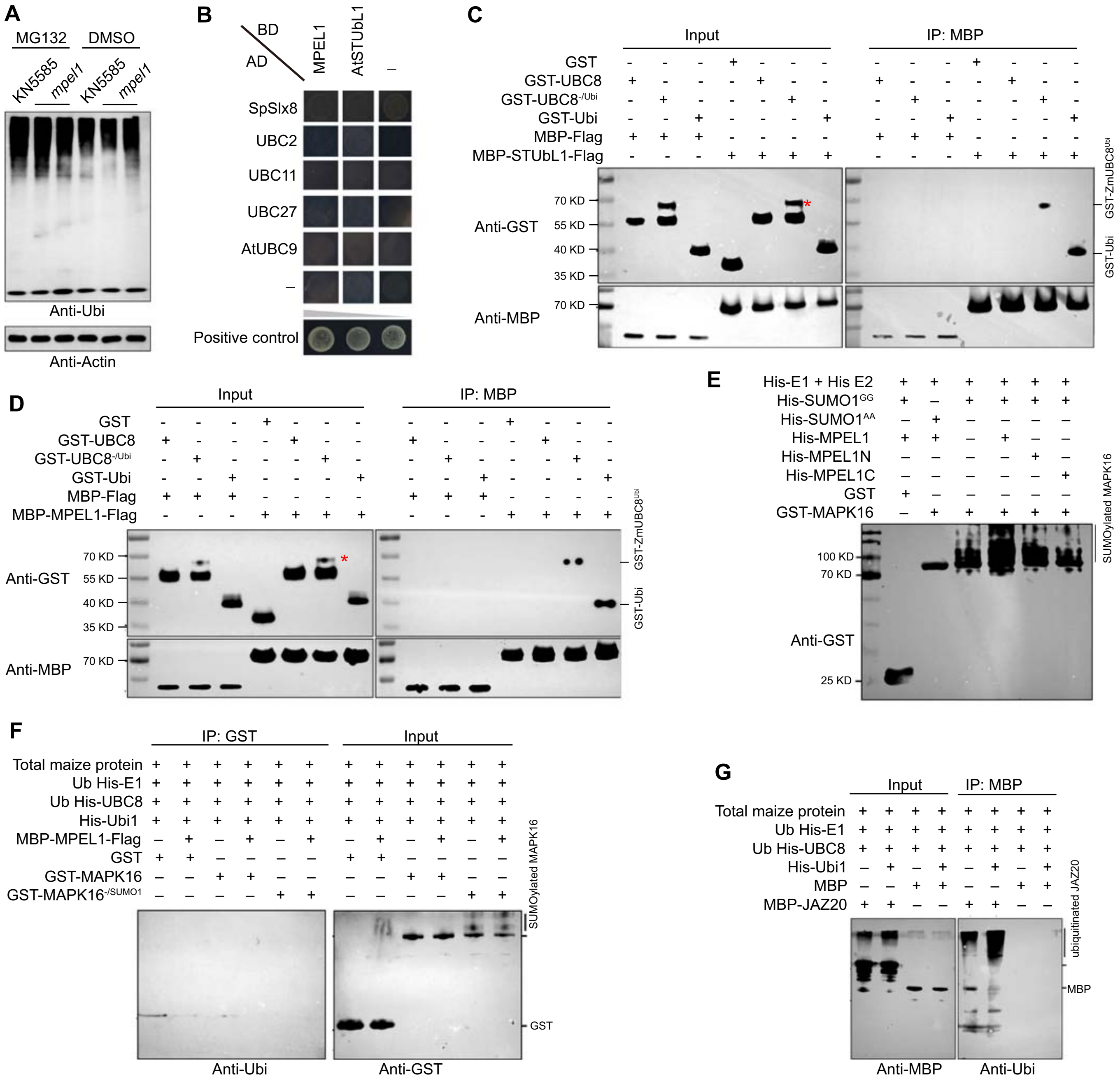

Figure EV2. MPEL1 functions as a SUMO ligase rather than a ubiquitin ligase.

(**A**) Profiles of ubiquitination in total root protein with or without the treatment of MG132. (**B**) Yeast two-hybrid analysis of the interaction between MPEL1, AtSTUbL1, yeast Slx8 and members of the ubiquitin systems. UBC2, UBC11, UBC27, and AtUBC9 serve as ubiquitin E2 conjugating enzymes. (**C**, **D**) Protein pulldown analyzing MPEL1 and AtSTUbL1 interaction with ubiquitin, UBC8, and pre-covalently linked Ubi-UBC8 complex. GST-UBC8$^{-/Ubi}$ indicates that UBC8 is covalently coupled with ubiquitin, resulting in both free UBC8 and ubiquitin-modified UBC8. Red asterisk indicates UBC8 covalently coupled to ubiquitin. The values above the GST-UBC8$^{-/Ubi}$ and GST-Ubi bands in the IP results represent the normalized signal intensities. Normalization was performed using the signal intensities of GST-UBC8$^{-/Ubi}$ and GST-Ubi bands in the Input, as well as the MBP-MPEL1-Flag/ MBP-STUbL1-Flag signal in the IP. (**E**) In vitro SUMOylation assay using purified proteins. The C-terminal RING domain (residues 176 to the end) and the N-terminal SIM region (residues 1–175) of MPEL1 were analyzed. (**F**) Recombinant GST-MAPK16 was incubated with the SUMO system for one hour to get free GST-MAPK16 and SUMO1-modified GST-MAPK16, which were then affinity-purified using GST antibody-coated gel beads. The corresponding elution protein was subsequently incubated with other components for in vitro ubiquitination assays. (**G**) In vitro ubiquitination assays of JAZ20.

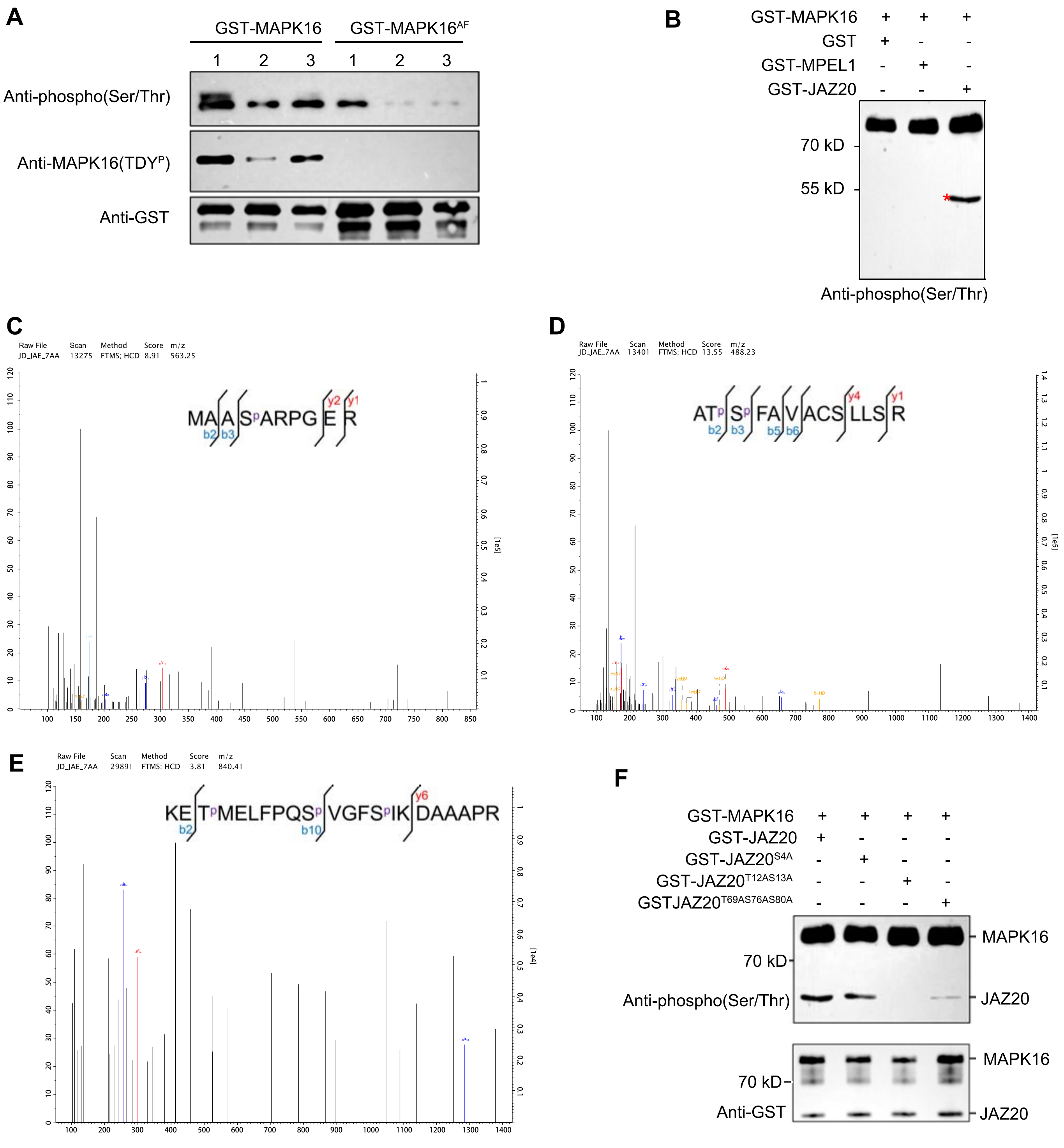

Figure EV3. JAZ20 is a phosphorylation substrate of MAPK16.

(**A**) MAPK16 autophosphorylates its TDY motif. For each protein, lane 1 shows the protein directly purified from prokaryotic expression. Lanes 2 and 3 represent proteins first dephosphorylated with λ-protein phosphatase (λ-PPase), followed by in vitro phosphorylation assays performed in the absence or presence of ATP, respectively. MAPK16^{AF} denotes the MAPK16 protein in which threonine at position 175 and tyrosine at position 177 are mutated to alanine and phenylalanine (TDY → ADF), respectively. (**B**) In vitro phosphorylation modification assays for GST-JAZ20 and GST-MPEL1. The band indicated by the red star is phosphorylated GST-JAZ20. (**C–E**) A total of six sites in three peptides from JAZ20 were identified by mass spectrometry as possible phosphorylation targets for MAPK16. More details are shown in Dataset EV1. (**F**) Recombinant GST-JAZ20 with different site-directed mutations were incubated with recombinant GST-MAPK16, and phosphorylation modifications were detected by immunoblotting.

 © The Author(s)

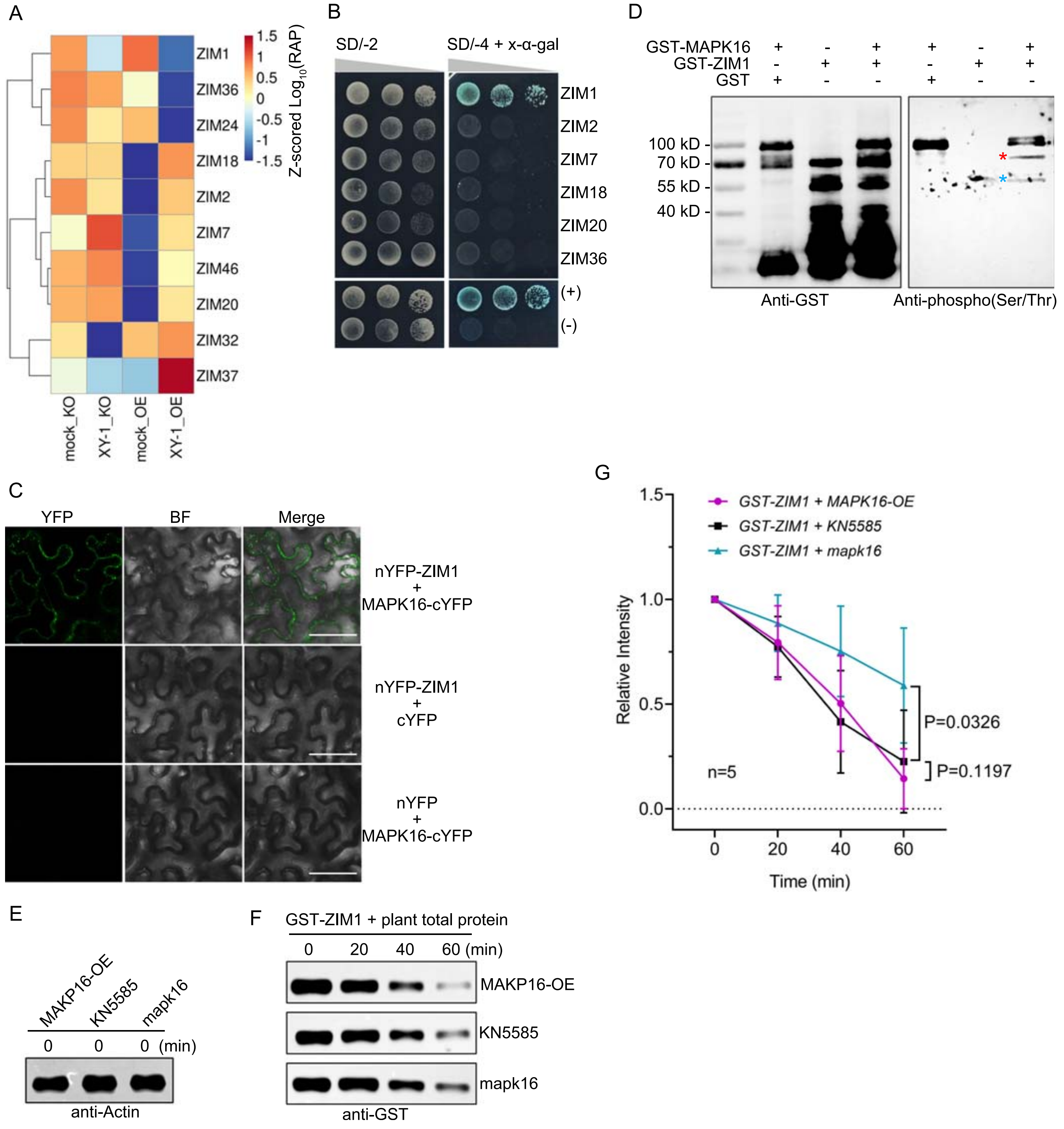

Figure EV4. ZIM1 (also known as JAZ17) is another target of MAPK16.

(**A**) The average expression levels of the ZIM family proteins identified in the comparative proteomic experiments are presented in a heat map. A log10 conversion and Z-score normalization of the average relative abundance of the proteins (RAP) were performed before heat mapping. (**B**) Yeast two-hybrid was used to analyze the interaction of MAPK16 with various ZIMs. (**C**) Bimolecular fluorescence complementation assay for protein–protein interactions. Scale bar = 50 μm. (**D**) In vitro phosphorylation modification assays for GST-ZIM1. The band marked with a red asterisk represents phosphorylated GST-ZIM1, while the band indicated by the blue asterisk may correspond to partially degraded and phosphorylated GST-ZIM1. (**E–G**) Equal quantities of recombinant GST-ZIM1 were incubated with the total protein of *MAPK16* overexpressing plants, knockout mutants, and the wild-type. Then, equal volumes of protein solution were used for immunoblotting at different time points. Five biological replicates were performed, and the signal intensity of different protein bands was calibrated using the zero-time point band as a reference in each replicate, and data were analyzed using a paired two-tailed Student's *t*-test. Data were presented as mean ± SD.

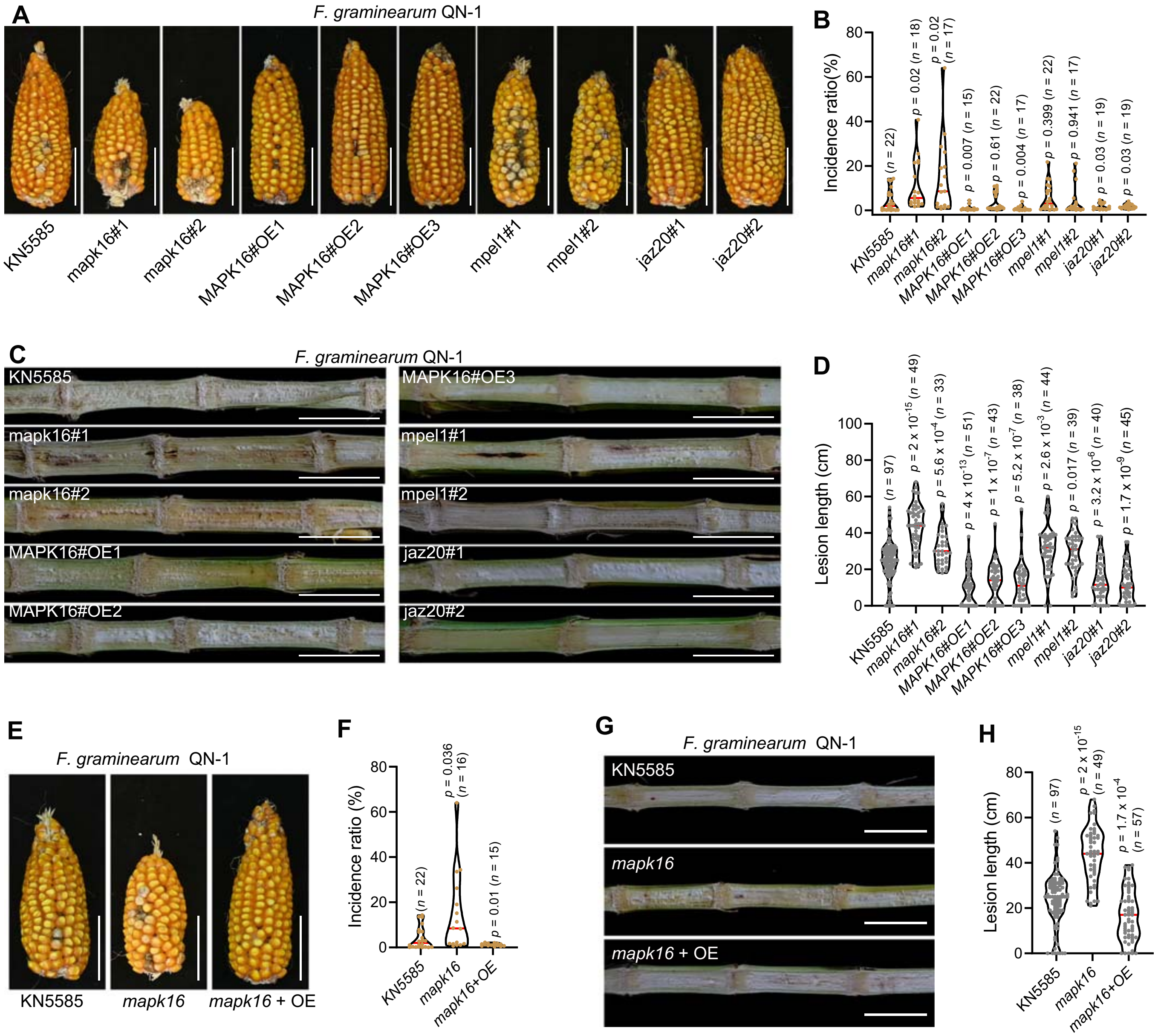

Figure EV5. Gene module *MPEL1-MAPK16-JAZ20* also regulates resistance against *F. graminearum* in maize.

(A–H) Phenotypes and corresponding statistical values of *F. graminearum* caused maize ear rot and stalk rot. *mapk16#1* and *mapk16#2* are CRISPR knockout mutants. **(E–H)** The *mapk16* + *OE* represents overexpression of *MAPK16* in the genetic context of the *mapk16* mutant. Panels (**B**, **D**) show one representative result from three independent experiments with similar outcomes. Panels (**F**, **H**) show one representative result from two independent experiments with similar outcomes. Data are shown as violin plots, with the median indicated by a short line. Data were analyzed using an unpaired two-tailed Student's *t*-test. Scale bars = 5 cm.
