# Supplementary material for: A new plant SUMO ligase, MPEL1, synergizes with MAPK16 to regulate resistance against Fusarium pathogens
Source: EMBO J. 2026 May 18;45(13):4670–93. doi: 10.1038/s44318-026-00811-2 (PMC13324008; doi:10.1038/s44318-026-00811-2)
Supplement: Supplementary file 4 — Peer Review File [file 44318_2026_811_MOESM4_ESM.pdf]

THE EMBO JOURNAL

A new plant SUMO ligase, MPEL1, synergizes with MAPK16 to regulate resistance against Fusarium pathogens

Xuanjun Feng, Weixiao Zhang, Huarui Guan, Haolan Yang, Na Wu, Yuting Chen, Xin Mao, Huihui Xiao, Mengling Li, Hao Xiong, Yinzhi Li, Zhaoyu Zhang, Muyuan Liu, Li Jia, Shuang Cha, Yizhen She, Xuemei Zhang, Haicheng Liao, Guangchao Sun, Yaxi Liu, Wenming Wang, Yunbi Xu, Yuelin Zhang, and Yanli Lu

Corresponding author(s): Yanli Lu (luyanli@sicau.edu.cn)

Review Timeline:

Submission Date:	5th Jan 26
Editorial Decision:	13th Feb 26
Appeal Received:	17th Feb 26
Editorial Decision:	20th Feb 26
Revision Received:	14th Apr 26
Editorial Decision:	28th Apr 26
Revision Received:	30th Apr 26
Accepted:	4th May 26

Editor: Hartmut Vodermaier

Transaction Report:

(Note: With the exception of the correction of typographical or spelling errors that could be a source of ambiguity, letters and reports are not edited. Depending on transfer agreements, referee reports obtained elsewhere may or may not be included in this compilation. Referee reports are anonymous unless the Referee chooses to sign their reports.)

Prof. Yanli Lu
Sichuan Agricultural University
State Key Laboratory of Crop Gene Exploration and Utilization in Southwest China
Chengdu, Sichuan 611130
China

13th Feb 2026

Re: EMBOJ-2026-123473
A new SUMO ligase synergizes with MAPK16 to regulate resistance against Fusarium pathogens

Dear Dr. Lu,

Thank you for submitting your manuscript on MPEL1 as a new SUMO E3 ligase involved in plant immunity to The EMBO Journal. It has now been reviewed by three referees with expertise in SUMO roles in plants, SUMOylation enzymes, and maize pathogen resistance, respectively, whose comments are copied below. Based on their reports, I am afraid we concluded that we currently cannot offer to publish this study.
As you will see, while referees 2 and 3 are overall more supportive pending addressing of some specific issues, referee 1 raises a large number of serious concerns that in our opinion compromise the conclusiveness of the results as presented. I will not repeat all the individual points of criticism in detail here, but major issues area the need for decisive validation of the in vitro findings in planta, ruling out alternative explanations, as well as rationalization of certain experiments or follow-up on particular targets. In light of the major experimental efforts necessary to satisfactorily address the key concerns, and the still unpredictable outcome of such follow-up work, I hope you understand that we are currently not in a position to invite (and thus to some degree commit to) a revised version of this manuscript.

Should further work along the lines suggested by the referees allow you to significantly strengthen and further develop the study, I would not exclude the possibility of once more looking into it and considering a potential resubmission. In this case, I would however appreciate if you then first contacted me with a detailed point-by-point response and summary of new results, so that we could determine whether a new version might be sufficiently promising to go back to our referees.

I am sorry that I cannot be more positive at the current stage, but hope that you will nevertheless find the referees' comments and suggestions helpful when considering how to proceed further with this study. Thank you once more for having had the opportunity to consider this work for publication.

Yours sincerely,

Hartmut Vodermaier

Hartmut Vodermaier, PhD
Senior Editor, The EMBO Journal
h.vodermaier@embojournal.org

****

Referee #1:

In the current manuscript, the authors identify a novel SUMO ligase, MPEL1, showing homology to an E4 SUMO ligase. They demonstrate its role in SUMOylating MAPK16, which in turn aids in the degradation of JAZ20. Although a thorough investigation has been conducted to establish the importance of MAPK16 and its role in imparting resistance to Fusarium infection, the manuscript has several serious concerns.
First, the classification of MPEL1 as an E4 ligase exhibiting E3 activity requires further clarification. At present, this conclusion is based solely on in vitro studies and therefore appears to be speculative. In addition, the entire study lacks any in planta protein-protein interaction analyses. Some experiments have no proper clarifications provided, while others are repeated across main and extended data figures. The detailed, point-wise comments are outlined below.
1. The abstract begins with a general statement on eukaryotic growth and defense. It would be more appropriate to focus specifically on plants and to introduce post-translational modifications. In addition, in line 20, SUMO ligase should be introduced earlier.
2. The rationale for selecting MAPK16 for this study is unclear. A more detailed explanation should be provided in the

introduction.
3. Loss of function of mapk16 results in shorter ears, as shown in Fig. 1a and Extended Fig. 1e. Cob size should be quantitatively measured. Moreover, in Fig. 1e-k, the complemented mapk16 plants show cob sizes similar to the mutant, suggesting improper functional complementation. This requires clarification.
4. In Extended Fig. 1q, colonization of germinating spores could be better resolved using cotton blue staining.
5. As mentioned in lines 113-114, uninfected samples are missing in Extended Fig. 2.
6. SUMOylation-deficient MAPK16 needs to be verified using in vivo approaches. Demonstrating MAPK16 SUMOylation in a prokaryotic system is inappropriate, as prokaryotes lack SUMO machinery. It is essential to demonstrate MAPK16 SUMOylation in N. benthamiana as well as in maize. It is also important to show the phenotype of MAPK16 SUMOylation deficient allele.
7. It is unclear how MAPK16 shows F. verticillioides-mediated induction, whereas overexpression of MAPK16 alone does not activate the defense gene cascade and requires clarification.
8. Although MAPK16 and MPEL1 interact, they exhibit distinct temporal expression patterns in Fig. 4f,g which is unexpected and requires explanation.
9. Lines 156-157: The authors should rationalize why ROS accumulation is induced only in MAPK16 overexpression lines, while mutant plants show levels comparable to WT. It is possible that MAPK16 overexpression induces endogenous MPEL1, thereby promoting ROS accumulation.
10. The co-IP band indicating interaction between MPEL1 and MAPK16 in Fig. 2c appears smeared and should be repeated.
11. Plants possess numerous E2 enzymes; for example, Arabidopsis alone has 32 reported E2s (Brillada et al., 2022). Therefore, the statement in lines 201-203 that AtSTUbL1 lacks ubiquitin ligase activity is inaccurate. A more comprehensive analysis is required before drawing this conclusion.
12. The interaction of MPEL1 with maize E2 (SCE1) must be shown to provide evidence of it being an E3 Ligase (PMID: 33006771).
13. In Extended Fig. 6B, a positive control should be included to confirm that the yeast two-hybrid assay is functional.
14. Two replicates of the same experiment are shown in Extended Fig. 6. Furthermore, in Extended Fig. 6d, a gradual reduction in STUbL protein levels is expected, particularly in the presence of ubiquitin as shown in panel c; however, this is not observed and requires clarification.
15. PR5 is predominantly reported to be salicylic acid-induced. The rationale for measuring its induction in the context of JAZ20 should be explained. Additionally, expression analysis of jasmonic acid biosynthetic LOX genes would strengthen the study.
16. Lines 201-203: The authors mention that AtSTUbL1 can complement the yeast rfp1/2 phenotype; this statement requires clarification.
17. The multiple sequence alignment of AtSTUbL1, MPEL1, Rfp1/2, and Slx8 in Extended Fig. 5a is poor. The phylogenetic analysis should include homologs from additional related plant species. Bootstrap values are missing from the phylogenetic tree.
18. The observation that MPEL1 binds to the ubiquitin-like protein ATG8 may indicate a role in ubiquitin-related activity.
19. In Extended Fig. 6F, the authors did not test whether MPEL1 exhibits ubiquitin ligase activity toward JAZ20.
20. In Extended Fig. 12, certain panels appear to be similar from Extended Fig. 1.
21. The phenotype of plants expressing the MAPK16 K526R mutant should be described.
22. Lines 250-251: The authors claim that SUMOylation of MAPK16 results in significantly slower degradation. However, based on Fig. b-c, the degradation rates appear comparable. Additionally, SUMOylated MAPK16 bands migrate near ~250 kDa, whereas previous blots show multiple SUMOylated species. This discrepancy should be addressed.
23. The effect of MPEL1-mediated SUMOylation on MAPK16 stability should be validated using proteasome inhibitors.
24. It is unclear why the authors tested whether MPEL1 SUMOylates JAZ20 in a MAPK16-dependent manner. If the hypothesis is that MAPK16 enhances MPEL1 activity, this should be tested using dose-dependent MAPK16 assays and by examining SUMOylation of other substrates such as ATG8b (Extended Fig. 6h).
25. In Extended Data Fig. 7, appropriate controls should demonstrate that MPEL1-Flag accumulates only in the presence of MAPK16-GFP and not with GFP alone. This should also be reflected in the co-localization images.
26. In Fig. 5C, BiFC suggests that the C-terminus of MPEL1 interacts with the N-terminus of JAZ20; however, no interaction is observed in vitro. This interaction should be validated using in vivo assays in N. benthamiana.
27. All lanes in immunoblots must be clearly labeled. For example, in Extended Fig. 8a, the identities of lanes 1 and 4 are missing.
28. It is strange why we observe some level of JAZ20 degradation even in a phospho-null JAZ20 mutant and requires clarification.
29. Several panels appear from the same experiment, such as Extended Fig. 8f, which is also shown in Fig. 6a.
30. In Fig. 6b and d, there is a discrepancy in JAZ20 degradation. GST-JAZ20 degrades when incubated with WT total protein (panel b), whereas degradation is negligible in panel d. This inconsistency requires clarification.
31. In Fig. 7a, showing JAZ20 stability in the presence of MAPK16 and MPEL1, the time point used should be specified. In vitro assays at 60 minutes show a reduction in JAZ20 in the presence of MAPK16.
32. In Fig. 7c, inclusion of a control showing JAZ20 stability in the absence of MPEL1 is essential to establish the role of MPEL1 as an E4 ligase.
33. In Fig. 7a, the cytoplasmic fraction of JAZ20 is poorly represented in the GFP channel and appears predominantly nuclear. Quantification of the nuclear-to-cytoplasmic ratio is recommended.
34. The functional relevance of MAPK16-mediated phosphorylation and degradation of ZIM1 is unclear and is not discussed. This should be addressed.
35. Lines 488-491: AtSTUbL lacks ubiquitin ligase activity is an overstatement. As mentioned earlier proper validation needs to be made with all Ub E2 ligases before coming to a conclusion.

36. SUMO E2 enzymes can also enhance SUMOylation in a dose-dependent manner even in the absence of E3 ligases. Therefore, it is important to show whether increasing E2 levels together with MPEL1 enhances SUMOylation of substrates such as MAPK16 or ATG8.
The current manuscript lacks in-planta experimental validation of MPEL1 role as a SUMO Ligase and focusses mostly on in-vitro studies. I would encourage to provide more in-planta protein-protein interaction studies and more controls to validate the findings of this study.

Referee #2:

In this manuscript, Feng and colleagues investigate the molecular basis of maize resistance to Fusarium pathogens. Their study begins with the observation that MAPK16 functions as a positive regulator of immunity against F. verticillioides. The authors then show that MPEL1, acting as a SUMO E3 ligase, stabilizes MAPK16 through SUMOylation. This stabilized MAPK16 then phosphorylates JAZ20, leading to its degradation, activation of defense genes, and ultimately enhanced resistance. The study is well-executed and provides strong evidence for these interactions that are initially identified through yeast-two-hybrid assays and subsequently validated by bifluorescence complementation, co-immunoprecipitation, and pull-down experiments. Furthermore, the authors pinpointed specific SUMOylation and phosphorylation sites using mutant analysis and/or proteomics. The localization of both proteins and interactions is determined and functional validation in maize confirms the role of these interactions in pathogen resistance.

This work thus provides important mechanistic insights into plant immunity obtained and confirmed through a variety of techniques. This work notably identifies a post-translational regulatory pathway that enhances defense responses without compromising growth. This is particularly relevant to agricultural applications aimed at improving crop resilience. Beyond plants, this study contribute to a broader understanding of kinase regulation and protein modification mechanisms like SUMOylation and Ubiquitination, thereby appealing to researchers in diverse fields.

Overall, I strongly support the publication of this article in EMBO Journal with the following minor concerns:
• Given recent literature highlighting the complexity in identifying true SUMO E3 ligases (e.g. TOPORS was initially shown to display SUMO E3 activity but a latter study showed that this activity was modest and independent of the RING domain with the current view being that TOPORS rather acts as a StUbL), it would be valuable to further characterize MPEL1's SUMOylation activity. Specifically, investigating whether the RING domain (residues 176-end) and/or the N-terminal SIM region (residues 1-175) are essential for this activity would provide additional information regarding the molecular determinants of the SUMO E3 activity. This could be performed using in vitro SUMOylation assay as performed in Fig. 3e.
• Quantifying the extent of MAPK16 stabilization by MPEL1 in Extended Data Fig. 7b would enhance the clarity of these results.
• Mention that the SUMO and Ubiquitin constructs used in yeast-two-hybrid experiments can be conjugated (i.e. they both end with GG). These experiments (e.g. Fig. 3b) can thus report covalent and nonconvalent interactions.
• The interaction between MPEL1 and MAPK16 in the cytoplasm/membrane is intriguing given that SUMOylation typically occurs in the nucleus. Further discussion of this observation could be insightful.
• Providing the exact residue boundaries for the N- and C-terminal truncations used in the study and including these numbers in Fig. 2a, along with visual representations of the truncations on protein schemes would improve clarity for readers.
• In Extended Data Fig. 5, the name of the protein (MPEL) appears to have been mistakenly included at the beginning of the sequence. Please remove "MPEL" at the beginning of the ZmMPEL1 sequence.
• In Extended data Figure 1b, is the overexpressed MAPK16 tagged? Its migration suggests a higher molecular weight than the native protein (assuming the native protein is the band below 60kDa).
• Indicate "Time (min)" in panels C and E of Fig. 6.

Referee #3:

This is a high-quality and mechanistically sophisticated study that integrates SUMOylation, MAPK signaling, and jasmonate repression into a coherent plant defense pathway with clear agronomic relevance. The identification of MPEL1 as a non-canonical SUMO E3 ligase, its role in stabilizing MAPK16, and the downstream MAPK16-JAZ20 phosphorylation-degradation module represent a genuinely novel conceptual advance. Overall, the work is strong and timely, and I recommend acceptance pending revision.
Major concerns
1. The authors conclude that MPEL1 functions as a SUMO E3 ligase. While the data supporting substrate SUMOylation are compelling, direct biochemical evidence of intrinsic E3 ligase activity remains limited. Additional clarification or experimental support distinguishing MPEL1 from adaptor-like or co-factor roles in SUMOylation would strengthen this conclusion.
2. The manuscript proposes that SUMOylation of MAPK16 at K526 is functionally important for disease resistance in planta. However, no genetic complementation or resistance assays using a MAPK16K526R variant are presented. Such experiments would be important to directly test whether this modification is required for the resistance phenotype.
3. The authors show that MAPK16 can also phosphorylate ZIM1/JAZ17, and proteomic analyses indicate that multiple JAZ

proteins are affected. It remains unclear why JAZ20 is positioned as the central regulatory node in this pathway rather than one of several functionally redundant or partially overlapping JAZ proteins. A clearer rationale, supported by genetic or functional specificity, would improve the conceptual clarity of this section.
4. The cell-death phenotype observed in tobacco is intriguing but currently underdeveloped. It is not clear whether this phenotype reflects a biologically relevant defense response or an artifact of heterologous overexpression. Additional discussion or supporting evidence would help to contextualize these observations.
Minor concerns
5. Language and clarity require minor editing throughout. Examples include:
◦ "bind to" → "binds to" (multiple instances)
◦ "may not dependent on ROS" → "may not be dependent on ROS"
◦ "pathwasys" → "pathways" (around line 399)Please also ensure consistent terminology throughout the manuscript, particularly for "SUMO E3 ligase" versus "SUMO ligase," and "STUbL" versus "STUBL."

*** As a service to authors, The EMBO Journal offers the possibility to directly transfer declined manuscripts to another EMBO Press title (EMBO Reports, EMBO Molecular Medicine, Molecular Systems Biology) or to the open access journal Life Science Alliance launched in partnership between EMBO Press, Rockefeller University Press and Cold Spring Harbor Laboratory Press. The full manuscript (including reviewer comments, where applicable and if chosen) will be automatically forwarded to the receiving journal, to allow for fast handling and a prompt decision on your manuscript. For more details of this service, and to transfer your manuscript to another EMBO title please follow this link:
Link Not Available

Dear Editor-in-Chief and Executive Editor,

As the Chinese Spring Festival is upon us, we also wish you and your family a joyful and prosperous day! We are writing to request a re-evaluation of our manuscript entitled A new SUMO ligase synergizes with MAPK16 to regulate resistance against Fusarium pathogens (Manuscript No.: EMBOJ-2026-123473), and we sincerely hope the editorial board will consider giving our work a second review.

We would like to express our sincere gratitude to Reviewer #1 for the rigorous and insightful comments on our manuscript. That said, **several of its key comments are inconsistent with the actual findings of our study, which may potentially mislead the Editor's evaluation.** After a careful and thorough analysis of all the comments, we provide point-by-point responses below, and we sincerely hope the editorial board will conduct a re-evaluation of our manuscript.

The reviewer pointed out that the conclusion that MPEL1 acts as an E4 ligase with E3 activity is merely speculative based on in vitro studies, and that the study lacks in planta protein-protein interaction analyses, with some experiments lacking clear explanations and others being repeated across main and extended figures. However, these viewpoints are inconsistent with the actual research results of our manuscript, which may be due to insufficient clarity in our writing. We clarify these core issues first as follows:

Our manuscript contains multiple compelling in vivo experimental results demonstrating that MPEL1 promotes the SUMOylation process, rather than the conclusion being solely based on in vitro studies as suggested. For instance, Figure 3a first revealed through antibody analysis that the loss of MPEL1 function reduces the SUMOylation of maize proteins without affecting ubiquitination modification—a typical in vivo experiment. This result already indicates the fundamental functional difference between MPEL1 and E4 ligases, as the loss of E4 ligase function leads to the accumulation of protein SUMOylation. Additionally, Figure 3f is another in vivo experiment that directly proves MPEL1 can promote the SUMOylation of MAPK16.

Regarding the comment that "the entire study lacks any in planta protein-protein interaction analyses", this is an overstatement that may mislead the Editor. To analyze protein-protein interactions, we employed both BiFC and CoIP assays, which are well-recognized in planta experimental approaches. In Figures 2 and 5, we respectively used four experimental methods (BiFC, Y2H, Pull-down, and CoIP) to

verify protein interactions, providing comprehensive and reliable evidence for the interactions between the target proteins.

As for the claim that "some experiments are repeated across main and extended data figures", this situation does not exist. Some experiments may appear similar at first glance, but a detailed examination reveals their essential differences. For example, Figure 3d and Extended Figure 6e are completely distinct experiments. Similarly, Extended Figure 6c and Extended Figure 6d are not two replicates of the same experiment as stated in Question 14. Another example is Figure 6a and Extended Figure 8f: Figure 6a mainly presents the validation results of the two phosphorylation sites T12 and S13 in JAZ20, while Extended Figure 8f provides supplementary validation of candidate phosphorylation sites such as S4, T69, S76, and S80. The two figures show data for different sites (with partial overlap) and jointly support the identification results of JAZ20 phosphorylation sites.

P**oint-by-point responses**

1. The abstract begins with a general statement on eukaryotic growth and defense. It would be more appropriate to focus specifically on plants and to introduce post-translational modifications. In addition, in line 20, SUMO ligase should be introduced earlier.

Reply1: We will revise the abstract as suggested. We will focus specifically on plant research and introduce post-translational modifications at the beginning, and also advance the introduction of SUMO ligase to an earlier position.

2. The rationale for selecting MAPK16 for this study is unclear. A more detailed explanation should be provided in the introduction.

Reply2: We will supplement a detailed explanation of the rationale for selecting MAPK16 for this study in the introduction section to make the research design more logical and clear.

3. Loss of function of mapk16 results in shorter ears, as shown in Fig. 1a and Extended Fig. 1e. Cob size should be quantitatively measured. Moreover, in Fig. 1e-k, the complemented mapk16 plants show cob sizes similar to the mutant, suggesting improper functional complementation. This requires clarification.

Reply3: We have rechecked the manuscript repeatedly and found that the result mentioned by the reviewer—"the complemented mapk16 plants show cob sizes similar to the mutant"—does not exist in our manuscript. Figure 1 only includes panels e-h, which do not show cob phenotypes. If the reviewer refers to Extended Figure 1, the cob photographs in panels g and k clearly show that the cob size of the mapk16 mutant was restored by OE-MAPK16. In addition, our study only focuses on disease resistance and yield traits, and has not investigated all agronomic traits such as cob length, cob diameter, and flowering stage.

4. In Extended Fig. 1q, colonization of germinating spores could be better resolved using cotton blue staining.

Reply4: We will try the cotton blue staining method suggested by the reviewer. Meanwhile, the experimental method we originally used can also clearly show the colonization of spores on the plant surface, and we can provide magnified images if needed.

5. As mentioned in lines 113-114, uninfected samples are missing in Extended Fig. 2.

Reply5: The 0 h time point in the experiment serves as the uninfected control. The description in Line 114—"while no differences were observed under pathogen-free conditions"—is based on the gene expression of different materials at the 0 h time point, and we will clarify this point in the figure legend for better understanding.

6. SUMOylation-deficient MAPK16 needs to be verified using in vivo approaches. Demonstrating MAPK16 SUMOylation in a prokaryotic system is inappropriate, as prokaryotes lack SUMO machinery. It is essential to demonstrate MAPK16 SUMOylation in N. benthamiana as well as in maize. It is also important to show the phenotype of MAPK16 SUMOylation deficient allele.

Reply6: We appreciate the valuable suggestion from the reviewer and will supplement in vivo analyses to detect the deSUMOylation of MAPK16 after mutating the lysine at position 526 to arginine (K526R). It should be noted that when we performed in vitro SUMOylation analysis using proteins expressed and purified from the prokaryotic expression system, we added relevant components including plant SUMO E1, E2, and SUMO1, which ensured the validity of the in vitro experiment. Regrettably, we are unable to provide the phenotype of transgenic plants expressing

the MAPK16 K526R mutant at present, as the generation of such site-directed mutant transgenic materials takes approximately one year, and the acquisition of related phenotypic data will require an additional 1-2 years due to the growth cycle of maize and experimental verification. We hope the reviewer can understand this practical experimental limitation.

7. It is unclear how MAPK16 shows F. verticillioides-mediated induction, whereas overexpression of MAPK16 alone does not activate the defense gene cascade and requires clarification.
Reply7: Through qRT-PCR and Western Blot analyses, we confirmed that MAPK16 is upregulated at both the transcriptional and protein levels upon induction by *Fusarium verticillioides*. Notably, under uninfected conditions (0 h), the basal expression levels of some PR genes in MAPK16-overexpressing materials are slightly higher than those in mapk16 knockout materials, indicating that the overexpression of MAPK16 exerts a positive regulatory effect on the maintenance of defense genes. However, the full activation of MAPK16 kinase activity may depend on the coordination of upstream and downstream signals triggered by Fusarium infection, and the accumulation of MAPK16 protein alone is insufficient to activate downstream defense genes at a high level. This characteristic precisely explains the core finding of our study that MAPK16-overexpressing lines enhance disease resistance without causing yield loss.

8. Although MAPK16 and MPEL1 interact, they exhibit distinct temporal expression patterns in Fig. 4f,g which is unexpected and requires explanation.
Reply8: It should be pointed out that the transcriptional expression patterns of MPEL1 and MAPK16 in our research results do not show any contradictory or unexplainable trends. Furthermore, it is a common biological phenomenon that two proteins with upstream and downstream regulatory relationships do not necessarily exhibit a perfectly synchronous expression pattern at the transcriptional level, as the expression and regulation of each protein are affected by multiple signaling pathways and regulatory factors in vivo.

9. Lines 156-157: The authors should rationalize why ROS accumulation is induced only in MAPK16 overexpression lines, while mutant plants show levels comparable

to WT. It is possible that MAPK16 overexpression induces endogenous MPEL1, thereby promoting ROS accumulation.

Reply9: MAPK16 is not a core basal initiator of ROS production in maize, but a regulatory factor for ROS amplification. Therefore, the overexpression of MAPK16 can promote ROS accumulation to enhance the plant's defense response against pathogens. In contrast, the ROS levels in mapk16 mutants are comparable to those in wild-type (WT) plants, which is because there are numerous basal ROS initiation and regulatory pathways in plants. When MAPK16 is deficient, these pathways can compensate for its function to maintain the basal level of ROS in vivo. Another possibility is that the loss of MAPK16 has a weak effect on ROS production, which cannot be detected by our current experimental detection methods due to sensitivity limitations. We will add this mechanistic explanation in the manuscript.

10. The co-IP band indicating interaction between MPEL1 and MAPK16 in Fig. 2c appears smeared and should be repeated.

Reply10: The co-IP result in Figure 2c clearly and reliably demonstrates the interaction between MPEL1 and MAPK16, with no ambiguity in the experimental conclusion. If the editorial board and the reviewer consider it necessary, we will repeat the experiment and provide a new, clearer image in the revised manuscript.

11. Plants possess numerous E2 enzymes; for example, Arabidopsis alone has 32 reported E2s (Brillada et al., 2022). Therefore, the statement in lines 201-203 that AtSTUbL1 lacks ubiquitin ligase activity is inaccurate. A more comprehensive analysis is required before drawing this conclusion.

Reply11: We appreciate the reviewer's correction on this point. Regarding the selection of ubiquitin E2 enzymes in our experiment, we provide the following explanations: first, the selection is based on the functional characteristics and research background of RNF4 and AtSTUbL, with a clear theoretical basis. Second, we screened E2 enzymes highly homologous to human UBCH5 (the E2 enzyme used in RNF4 research) from both maize and Arabidopsis for the experiment. Given the high conservation of the structure and function of E2 enzymes in eukaryotes, selecting representative core members for experimental verification is scientific and sufficient. We acknowledge that the statement in Line 203—"These results indicate that although AtSTUbL1 can complement the defective phenotype of yeast Rfp1/2 loss-of-function

mutants, it does not necessarily exhibit ubiquitin ligase activity"—is inappropriate, and we will revise this statement to make it more accurate and rigorous in the revised manuscript.

12. The interaction of MPEL1 with maize E2 (SCE1) must be shown to provide evidence of it being an E3 Ligase (PMID: 33006771).

Reply12: We have already verified the interaction between MPEL1 and Arabidopsis SCE1 using two experimental methods (Y2H and BiFC). As suggested by the reviewer, we will supplement the experiment to verify the interaction between MPEL1 and maize SCE1, and add the relevant results to the manuscript to provide more direct evidence for MPEL1 as a SUMO E3 ligase.

13. In Extended Fig. 6B, a positive control should be included to confirm that the yeast two-hybrid assay is functional.

Reply13: We will add the appropriate positive control in the yeast two-hybrid assay of Extended Figure 6B in the revised manuscript to confirm the functionality of the assay system and ensure the reliability of the experimental results.

14. Two replicates of the same experiment are shown in Extended Fig. 6. Furthermore, in Extended Fig. 6d, a gradual reduction in STUbL protein levels is expected, particularly in the presence of ubiquitin as shown in panel c; however, this is not observed and requires clarification.

Reply14: First, Extended Figure 6 does not show two replicates of the same experiment; instead, it presents verification experiments conducted for two different proteins (MPEL1 and AtSTUbL1) respectively. Second, the Pull-down assay in this figure was performed in vitro using proteins expressed and purified from the prokaryotic system. Since the reaction system does not contain the 26S proteasome, there is no ubiquitin-dependent protein degradation pathway in the in vitro system. Therefore, the abundance of STUbL1 protein conjugated with ubiquitin will not decrease, and the signal intensity shown in the figure is a reasonable reflection of the actual experimental results. We will clarify the experimental design and reaction system of this figure in the legend.

15. PR5 is predominantly reported to be salicylic acid-induced. The rationale for measuring its induction in the context of JAZ20 should be explained. Additionally, expression analysis of jasmonic acid biosynthetic LOX genes would strengthen the study.

Reply15: It should be noted that although PR5 is widely reported as a marker gene of the salicylic acid (SA) signaling pathway, it is not exclusive to the SA pathway. PR5 is located at the crossroads of the SA and jasmonic acid (JA) signaling pathways and can respond to the regulation of multiple immune signaling pathways. Existing studies have confirmed that the activation of the JA signaling pathway can induce PR5 expression directly or indirectly (see the references below). Therefore, the changes in PR5 expression in our study should be regarded as a marker of the activation of plant immune signaling pathways, rather than exclusive evidence for the SA pathway alone. In addition, we will supplement the expression analysis of JA biosynthetic LOX genes in the revised manuscript as suggested, to provide a more comprehensive analysis of the plant defense signaling pathway regulated by MAPK16 and MPEL1.

- Rukmini, Mishra, Satyabrata, et al. Differential expression of defense-related genes in chilli pepper infected with anthracnose pathogen *Colletotrichum truncatum* [J]. Physiological & Molecular Plant Pathology, 2017. DOI:10.1016/j.pmpp.2016.11.001.
- Xu Y, Chang P F L, Liu D, et al. Plant Defense Genes Are Synergistically Induced by Ethylene and Methyl Jasmonate [J]. The Plant Cell, 1994, 6(8):1077-1085. DOI:10.2307/3869886.

16. Lines 201-203: The authors mention that AtSTUbL1 can complement the yeast rfp1/2 phenotype; this statement requires clarification.

Reply16: This conclusion is a citation of the research result from the reference *Identification of Arabidopsis SUMO-interacting proteins that regulate chromatin activity and developmental transitions*. We will add the corresponding reference citation at the relevant position in the manuscript to ensure the traceability of the conclusion.

17. The multiple sequence alignment of AtSTUbL1, MPEL1, Rfp1/2, and Slx8 in Extended Fig. 5a is poor. The phylogenetic analysis should include homologs from

additional related plant species. Bootstrap values are missing from the phylogenetic tree.

Reply17: We will optimize the multiple sequence alignment of AtSTUbL1, MPEL1, Rfp1/2, and Slx8 in Extended Figure 5a to improve its quality. In addition, we will supplement the phylogenetic analysis by including homologs from more related plant species and add bootstrap values to the phylogenetic tree to make the evolutionary analysis more comprehensive and reliable.

18. The observation that MPEL1 binds to the ubiquitin-like protein ATG8 may indicate a role in ubiquitin-related activity.

Reply18: The specific binding of MPEL1 to the ubiquitin-like protein ATG8 indeed implies that MPEL1 may be involved in extended functions related to the ubiquitin system. However, it is important to clarify that this binding activity does not indicate that MPEL1 itself has ubiquitin ligase activity. We propose a more reasonable mechanistic model: MPEL1 may couple the SUMOylation modification with the ATG8-mediated autophagy pathway through a 'SUMOylation-ATG8b' axis, thereby participating in the SUMO-targeted autophagy process and playing a role in the ubiquitin-autophagy coordinated protein degradation network.

19. In Extended Fig. 6F, the authors did not test whether MPEL1 exhibits ubiquitin ligase activity toward JAZ20.

Reply19: In fact, we have performed the experiment mentioned by the reviewer to detect whether MPEL1 has ubiquitin ligase activity toward JAZ20, and the result shows that MPEL1 cannot promote the ubiquitination of JAZ20. This result was not included in the original manuscript because it is not consistent with the narrative logic of the study, which focuses on the SUMOylation mediated by MPEL1 rather than its ubiquitination function. If the editorial board and the reviewer consider it necessary, we will supplement this experimental result in the extended data of the revised manuscript.

20. In Extended Fig. 12, certain panels appear to be similar from Extended Fig. 1.

Reply20: We have carefully verified the two figures, and there are no identical panels between Extended Figure 12 and Extended Figure 1. Extended Figure 1 shows the phenotypic results after infection with *Fusarium verticillioides,* while Extended

Figure 12 presents the phenotypic results after infection with *Fusarium graminearum*, and the different pathogens are clearly labeled in both figures. This may be a misunderstanding by the reviewer due to the similar phenotypic detection methods, and we will add more distinct labels in the figure legends to avoid confusion.

21. The phenotype of plants expressing the MAPK16 K526R mutant should be described.

Reply21: This question is the same as Question 6. As explained in the reply to Question 6, we are currently unable to provide the phenotype of transgenic plants expressing the MAPK16 K526R mutant due to the long cycle required for the generation of maize transgenic materials and phenotypic verification. We sincerely hope the reviewer can understand this practical experimental constraint.

22. Lines 250-251: The authors claim that SUMOylation of MAPK16 results in significantly slower degradation. However, based on Fig. b-c, the degradation rates appear comparable. Additionally, SUMOylated MAPK16 bands migrate near ~250 kDa, whereas previous blots show multiple SUMOylated species. This discrepancy should be addressed.

Reply22: Regarding the conclusion that SUMOylation of MAPK16 significantly slows its degradation rate, we would like to emphasize to the reviewer that this finding is not based on a subjective visual judgment of band intensity, but on statistical analyses of multiple repeated experiments. We conducted quantitative analyses of the results of multiple independent experiments, and the data confirmed that the effect of SUMOylation on the stability of MAPK16 protein is statistically significant.

As for the slight differences in the SUMOylated bands observed in different experiments, we believe this may be caused by experimental fluctuations from technical operations such as Western Blot exposure time. As is well known to researchers familiar with Western Blot experiments, this technique is a semi-quantitative method, and a variation of approximately 20% between different replicates is considered normal—this is exactly why we performed multiple repeated experiments and conducted statistical analyses. It should be specially noted that the SUMOylated bands in the existing data cannot accurately reflect the actual molecular

weight of MAPK16 poly-SUMOylation modified forms, so it is inappropriate to over-infer the modification forms based on the band positions.

23. The effect of MPEL1-mediated SUMOylation on MAPK16 stability should be validated using proteasome inhibitors.

Reply23: It should be noted that the experiment in Figure 4b used maize total protein extracts co-incubated with in vitro purified proteins, aiming to simulate the natural intracellular environment of protein degradation and compare the degradation rates of MAPK16 with different modification states. Adding proteasome inhibitors would block the protein degradation pathway, which is contrary to the experimental purpose of this assay. However, if the editorial board and the reviewer consider it necessary, we will supplement the relevant experiment with proteasome inhibitors as a control in the revised manuscript to provide more comprehensive data.

24. It is unclear why the authors tested whether MPEL1 SUMOylates JAZ20 in a MAPK16-dependent manner. If the hypothesis is that MAPK16 enhances MPEL1 activity, this should be tested using dose-dependent MAPK16 assays and by examining SUMOylation of other substrates such as ATG8b (Extended Fig. 6h).

Reply24: We acknowledge that there is a flaw in the narrative logic of this part in the original manuscript. In the initial version of the manuscript, the contents of Figure 2 and Figure 5 were merged, so JAZ20 was introduced relatively early. Based on the evidence of in planta interaction between MPEL1 and JAZ20 from the tobacco BiFC experiment, we subsequently analyzed whether JAZ20 can be recognized and SUMOylated by MPEL1. However, in the current version of the manuscript, JAZ20 has not been introduced at the position of Figure 4, leading to an illogical narrative. We will revise the structure and narrative logic of the manuscript to adjust the presentation position of relevant content and make the research story more coherent and logical.

25. In Extended Data Fig. 7, appropriate controls should demonstrate that MPEL1-Flag accumulates only in the presence of MAPK16-GFP and not with GFP alone. This should also be reflected in the co-localization images.

Reply25: It should be noted that the reviewer may have misunderstood the experimental purpose of Extended Data Figure 7. This experiment aims to verify that

MPEL1-Flag promotes the accumulation of MAPK16-GFP, rather than that MAPK16-GFP induces the accumulation of MPEL1-Flag. The experimental control setting in the original manuscript is consistent with the experimental purpose, and the results are reliable.

26. In Fig. 5C, BiFC suggests that the C-terminus of MPEL1 interacts with the N-terminus of JAZ20; however, no interaction is observed in vitro. This interaction should be validated using in vivo assays in N. benthamiana.

Reply26: We would like to clarify to the reviewer that the labels MPEL1-cYFP and JAZ20-nYFP in the figure do not refer to the C-terminus of MPEL1 and the N-terminus of JAZ20, but indicate that the full-length sequences of MPEL1 and JAZ20 are fused to the cYFP and nYFP vectors, respectively. The experimental result clearly shows that the full-length MPEL1 interacts with the full-length JAZ20 in tobacco in planta. However, the subsequent in vitro Pull-down experiment failed to verify their direct interaction, indicating that the BiFC fluorescent signal observed in tobacco may result from an indirect interaction or a bridging effect mediated by other endogenous proteins in planta.

27. All lanes in immunoblots must be clearly labeled. For example, in Extended Fig. 8a, the identities of lanes 1 and 4 are missing.

Reply27: We acknowledge the insufficient labeling of partial immunoblot lanes in the original manuscript. Although the relevant information is clearly described in the figure legends, we will further improve the direct labeling of all lanes in the immunoblot figures to make the figures more intuitive and easy to read.

28. It is strange why we observe some level of JAZ20 degradation even in a phospho-null JAZ20 mutant and requires clarification.

Reply28: The reviewer is referring to Extended Figure 9b. The phenomenon of low-level degradation still observed in the phospho-null JAZ20 mutant is not an experimental anomaly, but a result determined by the complexity of plant protein degradation regulation. Plant protein degradation involves multiple parallel pathways; even if the phosphorylation-mediated degradation pathway is blocked, other degradation mechanisms (such as ubiquitination-independent degradation pathways or other kinase-mediated phosphorylation degradation pathways) can still function to a

certain extent. Therefore, the observation of low-level degradation of the phospho-null JAZ20 mutant is a completely reasonable normal biological phenomenon.

29. Several panels appear from the same experiment, such as Extended Fig. 8f, which is also shown in Fig. 6a.

Reply29: Extended Figure 8f and Figure 6a are not the same figure. As explained earlier, Figure 6a mainly presents the validation results of the two key phosphorylation sites T12 and S13 in JAZ20, while Extended Figure 8f provides supplementary validation of other candidate phosphorylation sites (S4, T69, S76, S80, etc.) in JAZ20. The two figures show data for different phosphorylation sites (with partial overlap) and jointly support the comprehensive identification results of JAZ20 phosphorylation sites, which is a complementary experimental design rather than a simple repetition.

30. In Fig. 6b and d, there is a discrepancy in JAZ20 degradation. GST-JAZ20 degrades when incubated with WT total protein (panel b), whereas degradation is negligible in panel d. This inconsistency requires clarification.

Reply30: We acknowledge that there are slight differences in the Western Blot bands of GST-JAZ20 degradation between Figure 6b and Figure 6d, but this is not the "negligible degradation" in Figure 6d as described by the reviewer—the degradation of GST-JAZ20 in Figure 6d is clearly observable. As mentioned in the reply to Question 22, Western Blot is a semi-quantitative experimental technique, and a variation of approximately 20% between different experimental replicates is normal due to technical factors such as sample loading, protein extraction efficiency, and exposure time. This is the key reason why we performed multiple independent repeated experiments and conducted statistical analyses of the results. The Western Blot images shown in the manuscript are representative results of a single experiment, and the focus should be on the statistically significant results of multiple repeated experiments rather than the slight differences in individual band intensity.

31. In Fig. 7a, showing JAZ20 stability in the presence of MAPK16 and MPEL1, the time point used should be specified. In vitro assays at 60 minutes show a reduction in JAZ20 in the presence of MAPK16.

Reply31: The experiment in Figure 7a involves the co-expression of MAPK16, MPEL1, and JAZ20 in tobacco leaves, and laser confocal detection was performed after 36 hours of normal expression—this is an in planta experimental system, which is completely different from the in vitro experimental conditions (60 minutes incubation) mentioned by the reviewer. The different experimental systems and time points are determined by the experimental design and research purpose, and we will add a clear description of the experimental time point and system in the figure legend to avoid confusion.

32. In Fig. 7c, inclusion of a control showing JAZ20 stability in the absence of MPEL1 is essential to establish the role of MPEL1 as an E4 ligase.
Reply32: We would like to clarify to the reviewer that our research has never claimed that MPEL1 is an E4 ligase; our conclusion from the beginning to the end is that MPEL1 is a SUMO E3 ligase rather than an E4 ligase. The experimental purpose of Figure 7c is to prove that MPEL1 can promote the phosphorylation of JAZ20 by MAPK16, not to verify the E4 ligase function of MPEL1. The experimental design and control setting of this figure are consistent with its research purpose, and we will add a more detailed explanation of the experimental purpose in the figure legend to avoid misunderstanding of the research content.

33. In Fig. 7a, the cytoplasmic fraction of JAZ20 is poorly represented in the GFP channel and appears predominantly nuclear. Quantification of the nuclear-to-cytoplasmic ratio is recommended.
Reply33: We have attempted to perform the nuclear-to-cytoplasmic ratio quantification analysis as suggested by the reviewer. However, this quantification method cannot well reflect the differences in protein levels between different experimental groups. The current detection method in the manuscript can not only reflect the subcellular distribution (nuclear and cytoplasmic) of JAZ20 protein but also show the differences in the total protein abundance of JAZ20 between different groups, which is more suitable for the research purpose of this experiment (analyzing the regulation of JAZ20 stability and distribution by MAPK16 and MPEL1).

34. The functional relevance of MAPK16-mediated phosphorylation and degradation of ZIM1 is unclear and is not discussed. This should be addressed.

Reply34: We have provided sufficient experimental evidence for the phosphorylation of ZIM1 (JAZ17) by MAPK16 and the subsequent promotion of ZIM1 degradation in the manuscript. We infer that the reviewer hopes us to provide phenotypic data of ZIM1-related transgenic materials. Considering that the functions of JAZ family members have been extensively reported in numerous previous studies and their action mechanisms are highly conserved, we did not generate ZIM1 transgenic materials after thoroughly elucidating the regulatory mechanism of MAPK16 on JAZ20 (the key JAZ family member in this study). We believe that this does not affect the core findings and conclusions of our research, as the conserved regulatory mechanism of MAPK16 on JAZ proteins has been sufficiently verified through the in-depth analysis of JAZ20.

35. Lines 488-491: AtSTUbL lacks ubiquitin ligase activity is an overstatement. As mentioned earlier proper validation needs to be made with all Ub E2 ligases before coming to a conclusion.
Reply35: We acknowledge that the relevant statement in Lines 488-491 is an overstatement and inappropriate. We will revise this statement in the revised manuscript to make it more accurate and rigorous, taking into account the conservation and diversity of E2 enzyme functions and the limitations of our experimental verification.

36. SUMO E2 enzymes can also enhance SUMOylation in a dose-dependent manner even in the absence of E3 ligases.
Reply36: We currently do not fully understand the specific research significance and experimental design of this suggested assay for our study. However, if the editorial board and the reviewer consider this experiment necessary to supplement the research content, we are willing to design and perform the relevant experiment and add the results to the revised manuscript in accordance with the suggestion.

We fully acknowledge that providing phenotypic data for the transgenic lines of the *mapk16* mutant complemented with MAPK16(K526R) would greatly enhance the completeness of this study. In fact, this was the primary reason for our manuscript being rejected by *Nature*. We sincerely regret this limitation, but after nearly a decade of dedicated work on this research, practical constraints have led us to the difficult

decision to submit the current findings for publication first. Generating the MAPK16(K526R)-complemented *mapk16* transgenic lines and characterizing their phenotypes will take an additional 3 to 4 years, and we humbly ask for your understanding of our inability to provide these genetic materials at the present stage.

That said, we believe no research is ever truly perfect, and a single manuscript cannot address all outstanding questions in a given field. Our current findings are sufficiently robust to support the following key conclusions:

First, MPEL1 is a previously uncharacterized SUMO ligase whose function diverges distinctly from the canonical activity of its STUbL homologs.

Second, we uncover a novel functional paradigm for the C-terminal domain (CTD) of plant-specific D-subgroup MAPKs—a long-uncharacterized region. For MAPK16, this domain fulfills two critical roles: it harbors Lys526, the site of MPEL1-mediated SUMOylation that enhances MAPK16 protein stability, and it acts as a docking interface for JAZ family repressors (JAZ20 and JAZ17), thus resolving the long-standing enigma of the biological function of the D-subgroup MAPK CTD.

Third, MAPK16 directly phosphorylates JAZ20 at Thr12 and Ser13, triggering its proteasomal degradation and thereby activating broad-spectrum resistance against *Fusarium verticillioides* and *F. graminearum*.

Fourth, overexpression of MAPK16 or knockout of *JAZ20* enhances maize resistance to Fusarium pathogens without compromising yield, providing valuable molecular targets for crop genetic improvement.

These findings are both significant and novel, and we believe they meet the publication standards of the *EMBO Journal*.

Overall, we feel that Reviewer #1 held a somewhat hostile attitude toward our work, to the point of seemingly distorting certain experimental results, which may have the potential to mislead the Editor’s judgment. We therefore earnestly hope the editorial board will re-evaluate our manuscript and consider granting us the opportunity to revise it, or alternatively, assign an independent reviewer to assess our work. We eagerly await your prompt response, which will allow us to plan our next steps—either revising and supplementing the manuscript as required or submitting it to another journal.

Best Regards,

All Authors

Prof. Yanli Lu
Sichuan Agricultural University
State Key Laboratory of Crop Gene Exploration and Utilization in Southwest China
Chengdu, Sichuan 611130
China

20th Feb 2026

Re: EMBOJ-2026-123473R-Q
A new SUMO ligase synergizes with MAPK16 to regulate resistance against Fusarium pathogens

Dear Dr. Lu,

Thank you for your feedback on our decision on your recent EMBO Journal submission. I have now had a chance to consider your points in detail, and I appreciate that your thoughtful responses may potentially clarify a number of referee 1's major criticisms. In this light, I would be happy to give you an opportunity to revise and resubmit the manuscript, so that we could send it back to the original referees and (if needed) additional expert arbitrators. While I understand that in-depth characterization of in planta phenotypes of non-SUMOylatable MAPK16 is not realistic within the scope of the present study, it would certainly be important to answer all other major concerns raised in the initial round to the referees' satisfaction.

I should add it is our policy to consider only a single round of major revision, making it important to comprehensively respond to all points at the time of resubmission. We would be happy to grant a deadline extended beyond the regular three-months revision period if this should be helpful, and our 'scooping protection' (meaning that competing work appearing elsewhere in the meantime will not affect our considerations of your study) would of course remain valid even throughout such an extension.

Further information on preparing, formatting and uploading a revised manuscript can be found below and in our Guide to Authors. I look forward to receiving your revision.

Yours sincerely,

Hartmut Vodermaier

Hartmut Vodermaier, PhD
Senior Editor, The EMBO Journal
h.vodermaier@embojournal.org

*** PLEASE NOTE: All revised manuscript are subject to initial checks for completeness and adherence to our formatting guidelines. Revisions may be returned to the authors and delayed in their editorial re-evaluation if they fail to comply to the following requirements. As a first step please read our guidelines for revised submissions:
https://link.springer.com/journal/44318/submission-guidelines#cms-Revised-submissions

1) Every manuscript requires a Data Availability section (even if only stating that no deposited datasets are included). Primary datasets or computer code produced in the current study have to be deposited in appropriate public repositories prior to resubmission, and reviewer access details provided in case that public access is not yet allowed.

2) Each figure legend must specify
- size of the scale bars that are mandatory for all micrograph panels
- the statistical test used to generate error bars and P-values
- the type error bars (e.g., S.E.M., S.D.)
- the number (n) and nature (biological or technical replicate) of independent experiments underlying each data point
- Figures may not include error bars for experiments with n<3; scatter plots showing individual data points should be used instead.

3) Revised manuscript text (including main tables, and figure legends for main and EV figures) has to be submitted as editable text file (e.g., .docx format). We encourage highlighting of changes (e.g., via text color) for the referees' reference.

4) Each main and each Expanded View (EV) figure should be uploaded as individual production-quality files (preferably in .eps, .tif, .jpg formats). For suggestions on figure preparation/layout, please refer to our Figure Preparation Guidelines:
https://media.springernature.com/original/springer-cms/rest/v1/content/27825798/data/v1

5) Point-by-point response letters should include the original referee comments in full together with your detailed responses to them (and to specific editor requests if applicable), and also be uploaded as editable (e.g., .docx) text files.

6) Please complete our Author Checklist, and make sure that information entered into the checklist is also reflected in the manuscript; the checklist will be available to readers as part of the Review Process File.

7) All authors listed as (co-)corresponding need to deposit, in their respective author profiles in our submission system, a unique ORCiD identifier linked to their name. Please see our Guide to Authors for detailed instructions.

8) Please note that supplementary information at EMBO Press has been superseded by the 'Expanded View' for inclusion of additional figures, tables, movies or datasets; with up to five EV Figures being typeset and directly accessible in the HTML version of the article.

9) To facilitate reproducibilty and cross-laboratory adoption of methodologies, please structure the Materials & Methods section as outlined in our guide to authors, including a completed Reagents and Tools Table.

10) Digital image enhancement is acceptable practice, as long as it accurately represents the original data and conforms to community standards. If a figure has been subjected to significant electronic manipulation, this must be clearly noted in the figure legend and/or the 'Materials and Methods' section. The editors reserve the right to request original versions of figures and the original images that were used to assemble the figure. Finally, we generally encourage uploading of numerical as well as gel/blot image source data.

At EMBO Press we ask authors to provide source data for the main manuscript figures. You will receive a separate email with instructions for providing source data with your revised manuscript, including how to upload and organize the files.

In the interest of ensuring the conceptual advance provided by the work, we recommend submitting a revision within 3 months (21st May 2026). Please discuss the revision progress ahead of this time with the editor if you require more time to complete the revisions. Use the link below to submit your revision:

Link Not Available

Point-by-point responses to reviewers

Referee #1:

1. The abstract begins with a general statement on eukaryotic growth and defense. It would be more appropriate to focus specifically on plants and to introduce post-translational modifications. In addition, in line 20, SUMO ligase should be introduced earlier.

Reply1: We have revised the abstract as suggested. Line19-21. For your convenience, the important modifications in the manuscript have been highlighted in yellow.

2. The rationale for selecting MAPK16 for this study is unclear. A more detailed explanation should be provided in the introduction.

Reply2: We have added further details regarding the rationale for selecting *MAPK16* for this study at the beginning of the Results section, thereby strengthening the logical coherence and clarity of the research design. Line93-94.

3. Loss of function of mapk16 results in shorter ears, as shown in Fig. 1a and Extended Fig. 1e. Cob size should be quantitatively measured. Moreover, in Fig. 1e-k, the complemented mapk16 plants show cob sizes similar to the mutant, suggesting improper functional complementation. This requires clarification.

Reply3: We have rechecked the manuscript repeatedly and found that the result mentioned by you—"the complemented mapk16 plants show cob sizes similar to the mutant"—does not exist in our manuscript. Figure 1 only includes panels e-h, which do not show cob phenotypes. If you refer to Extended Figure 1, the cob photographs in panels g and k clearly show that the cob size of the mapk16 mutant was restored by OE-MAPK16. In addition, our study only focuses on disease resistance and yield traits, and has not investigated all agronomic traits such as cob length, cob diameter, and flowering time.

4. In Extended Fig. 1q, colonization of germinating spores could be better resolved using cotton blue staining.

Reply4: We appreciate this valuable suggestion. The compromised image quality in the merged PDF may have given the impression that the resolution of our staining method is insufficient. In fact, the staining method we adopted yields results

comparable to cotton blue staining, and the colonization of spores on the plant surface can be clearly observed in the original high-resolution images.

5. As mentioned in lines 113-114, uninfected samples are missing in Extended Fig. 2.
Reply5: In the original version of the results, the 0 h time point in the experiment serves as the uninfected control. The description in Line 114—"while no differences were observed under pathogen-free conditions"—is based on the gene expression of different materials at the 0 h time point. In this revised version, we have adopted your suggestion and set up a sterile water treatment group as a control for each time point. The experimental results are consistent with those of the original version, and the detailed data are presented in **Appendix Fig. S1**.

6. SUMOylation-deficient MAPK16 needs to be verified using in vivo approaches. Demonstrating MAPK16 SUMOylation in a prokaryotic system is inappropriate, as prokaryotes lack SUMO machinery. It is essential to demonstrate MAPK16 SUMOylation in N. benthamiana as well as in maize. It is also important to show the phenotype of MAPK16 SUMOylation deficient allele.
Reply6: We appreciate the valuable suggestion from you and added in vivo assays to detect the deSUMOylation of MAPK16 after mutating the lysine at position 526 to arginine (K526R). Results showed that MAPK16^{K526R} cannot be SUMOylated in vivo, with detailed data presented in **Fig. 4B**. It should be noted that when we performed in vitro SUMOylation analysis using proteins expressed and purified from the prokaryotic expression system, we added relevant components including plant SUMO E1, E2, and SUMO1, which ensured the validity of the in vitro experiment. Regrettably, we are unable to provide the phenotype of transgenic plants expressing the MAPK16^{K526R} mutant at present, as the generation of such site-directed mutant transgenic materials takes approximately one year, and the acquisition of related phenotypic data will require an additional 1-2 years due to the growth cycle of maize and experimental verification. We would be grateful if you could kindly understand this practical experimental limitation.

7. It is unclear how MAPK16 shows *F. verticillioides*-mediated induction, whereas overexpression of MAPK16 alone does not activate the defense gene cascade and requires clarification.

Reply7: Through qRT-PCR and Western Blot analyses, we confirmed that MAPK16 is upregulated at both the transcriptional and protein levels upon induction by *Fusarium verticillioides*. Notably, under uninfected conditions, the basal expression levels of some PR genes in MAPK16-overexpressing materials are slightly higher than those in *mapk16* knockout plants, indicating that the overexpression of MAPK16 exerts a positive regulatory effect on the maintenance of defense genes. However, the full activation of MAPK16 kinase activity may depend on the coordination of upstream and downstream signals triggered by Fusarium infection, and the accumulation of MAPK16 protein alone is insufficient to activate downstream defense genes at a high level. Our proteomic data further corroborate that overexpression of MAPK16 alone is insufficient to activate downstream defense genes (Table EV5). This characteristic precisely explains the core finding of our study that MAPK16-overexpressing lines enhance disease resistance without causing yield loss.

8. Although MAPK16 and MPEL1 interact, they exhibit distinct temporal expression patterns in Fig. 4f,g which is unexpected and requires explanation.

Reply8: We believe the reference is to Extended Fig. 4f and g. It should be pointed out that the transcriptional expression patterns of MPEL1 and MAPK16 in our research results do not show any contradictory or unexplainable trends. Furthermore, it is a common biological phenomenon that two proteins with upstream and downstream regulatory relationships do not necessarily exhibit a perfectly synchronous expression pattern at the transcriptional level, as the expression and regulation of each protein are affected by multiple signaling pathways and regulatory factors in vivo.

9. Lines 156-157: The authors should rationalize why ROS accumulation is induced only in MAPK16 overexpression lines, while mutant plants show levels comparable to WT. It is possible that MAPK16 overexpression induces endogenous MPEL1, thereby promoting ROS accumulation.

Reply9: MAPK16 is not a core basal initiator of ROS production in maize, but a regulatory factor for ROS amplification. Therefore, the overexpression of MAPK16 can promote ROS accumulation to enhance the plant's defense response against pathogens. In contrast, the ROS levels in *mapk16* mutants are comparable to those in

wild-type (WT) plants, which is because there are numerous basal ROS initiation and regulatory pathways in plants. When MAPK16 is deficient, these pathways may compensate for its function to maintain the basal level of ROS in vivo. Another possibility is that the loss of MAPK16 has a weak effect on ROS production, which cannot be detected by our current experimental detection methods due to sensitivity limitations.

10. The co-IP band indicating interaction between MPEL1 and MAPK16 in Fig. 2c appears smeared and should be repeated.

Reply10: The co-IP result in Figure 2c clearly and reliably demonstrates the interaction between MPEL1 and MAPK16, with no ambiguity in the experimental conclusion. Nevertheless, we respect your suggestion, repeated the experiment, and have provided a clearer image in the revised manuscript.

11. Plants possess numerous E2 enzymes; for example, Arabidopsis alone has 32 reported E2s (Brillada et al., 2022). Therefore, the statement in lines 201-203 that AtSTUbL1 lacks ubiquitin ligase activity is inaccurate. A more comprehensive analysis is required before drawing this conclusion.

Reply11: We appreciate your correction on this point. Regarding the selection of ubiquitin E2 enzymes in our experiment, we provide the following explanations: first, the selection is based on the functional characteristics and research background of RNF4 and AtSTUbL, with a clear theoretical basis. Second, we screened E2 enzymes highly homologous to human UBCH5 (the E2 enzyme used in RNF4 research) from both maize and Arabidopsis for the experiment. Given the high conservation of the structure and function of E2 enzymes in eukaryotes, selecting representative core members for experimental verification is scientific and sufficient. We acknowledge that the statement in Line 203—"These results indicate that although AtSTUbL1 can complement the defective phenotype of yeast Rfp1/2 loss-of-function mutants, it does not necessarily exhibit ubiquitin ligase activity"—is inappropriate, and we have removed this statement.

12. The interaction of MPEL1 with maize E2 (SCE1) must be shown to provide evidence of it being an E3 Ligase (PMID: 33006771).

Reply12: We have already verified the interaction between MPEL1 and Arabidopsis SCE1 using two experimental methods (Y2H and BiFC). As your suggestion, we have supplemented the experiment to verify the interaction between MPEL1 and maize SCE1, and added the relevant results to the manuscript to provide more direct evidence for MPEL1 as a SUMO E3 ligase (**Appendix Fig. S5**).

13. In Extended Fig. 6B, a positive control should be included to confirm that the yeast two-hybrid assay is functional.

Reply13: We have added the appropriate positive control in the yeast two-hybrid assay of Extended Figure 6B in the revised manuscript to confirm the functionality of the assay system and ensure the reliability of the experimental results. The new figure name is **Fig. EV2B**.

14. Two replicates of the same experiment are shown in Extended Fig. 6. Furthermore, in Extended Fig. 6d, a gradual reduction in STUbL protein levels is expected, particularly in the presence of ubiquitin as shown in panel c; however, this is not observed and requires clarification.

Reply14: First, Extended Figure 6 does not show two replicates of the same experiment; instead, it presents verification experiments conducted for two different proteins (MPEL1 and AtSTUbL1) respectively. Second, the Pull-down assay in this figure was performed in vitro using proteins expressed and purified from the prokaryotic system. Since the reaction system does not contain the 26S proteasome, there is no ubiquitin-dependent protein degradation pathway in the in vitro system. Therefore, the abundance of STUbL1 protein conjugated with ubiquitin will not decrease, and the signal intensity shown in the figure is a reasonable reflection of the actual experimental results. Indeed, as you noted, the band signals of MBP-STUbL1-Flag and MBP-Flag in panel 6c appear to gradually decrease from left to right, but this is not caused by ubiquitination-mediated degradation, but rather potential systematic errors during Western blot development. This does not affect our interpretation of the results, as the corresponding band signals after IP do not show a similar trend.

15. PR5 is predominantly reported to be salicylic acid-induced. The rationale for measuring its induction in the context of JAZ20 should be explained. Additionally,

expression analysis of jasmonic acid biosynthetic LOX genes would strengthen the study.

Reply15: It should be noted that although PR5 is widely reported as a marker gene of the salicylic acid (SA) signaling pathway, it is not exclusive to the SA pathway. PR5 is located at the crossroads of the SA and jasmonic acid (JA) signaling pathways and can respond to the regulation of multiple immune signaling pathways. Existing studies have confirmed that the activation of the JA signaling pathway can induce PR5 expression directly or indirectly (see the references below). Therefore, the changes in PR5 expression in our study should be regarded as a marker of the activation of plant immune signaling pathways, rather than exclusive evidence for the SA pathway alone. In addition, we supplemented the expression analysis of LOX genes in the revised manuscript as suggested, to provide a more comprehensive analysis of the plant defense signaling pathway regulated by MAPK16 and MPEL1. Briefly, we examined the expression of four LOX genes, and three of these members showed more robust induction by XY-1 in MAPK16-overexpressing lines (**Appendix Fig. S1**).

- Rukmini, Mishra, Satyabrata, et al. Differential expression of defense-related genes in chilli pepper infected with anthracnose pathogen *Colletotrichum truncatum* [J]. Physiological & Molecular Plant Pathology, 2017. DOI:10.1016/j.pmpp.2016.11.001.
- Xu Y, Chang P F L, Liu D, et al. Plant Defense Genes Are Synergistically Induced by Ethylene and Methyl Jasmonate [J]. The Plant Cell, 1994, 6(8):1077-1085. DOI:10.2307/3869886.

16. Lines 201-203: The authors mention that AtSTUbL1 can complement the yeast rfp1/2 phenotype; this statement requires clarification.

Reply16: The full sentence is “These results indicate that although AtSTUbL1 can complement the defective phenotype of yeast Rfp1/2 loss-of-function mutants, it does not necessarily exhibit ubiquitin ligase activity.”. This conclusion is a citation of the research result from the reference ‘*Identification of Arabidopsis SUMO-interacting proteins that regulate chromatin activity and developmental transitions*’. Given that you objected to our description that “…it does not necessarily exhibit ubiquitin ligase activity” in comment 11, we have accordingly removed this sentence.

17. The multiple sequence alignment of AtSTUbL1, MPEL1, Rfp1/2, and Slx8 in Extended Fig. 5a is poor. The phylogenetic analysis should include homologs from additional related plant species. Bootstrap values are missing from the phylogenetic tree.

Reply17: We have supplemented the phylogenetic analysis by including more homologs from related plant species and add bootstrap values to the phylogenetic tree to make the evolutionary analysis more comprehensive and reliable. The new figure name is **Appendix Figure S4.**

18. The observation that MPEL1 binds to the ubiquitin-like protein ATG8 may indicate a role in ubiquitin-related activity.

Reply18: We agree with your speculation. The specific binding of MPEL1 to the ubiquitin-like protein ATG8b indeed implies that MPEL1 may be involved in extended functions related to the ubiquitin system. However, it is important to clarify that this binding activity does not indicate that MPEL1 itself has ubiquitin ligase activity. We propose a more reasonable mechanistic model: MPEL1 may couple the SUMOylation modification with the ATG8-mediated autophagy pathway through a 'SUMOylation-ATG8b' axis, thereby participating in the SUMO-targeted autophagy process and playing a role in the ubiquitin-autophagy coordinated protein degradation network. We have supplemented a discussion of this model in the Discussion section "***MPEL1* is a new SUMO ligase**".

19. In Extended Fig. 6F, the authors did not test whether MPEL1 exhibits ubiquitin ligase activity toward JAZ20.

Reply19: The objective of this experiment was to verify the functionality of the ubiquitination system used in our study. Since the degradation of JAZ proteins is well-documented to be regulated by the ubiquitination system, we used JAZ20 as a positive control to validate the functionality of our ubiquitination system, and did not aim to investigate whether MPEL1 can mediate the ubiquitination of JAZ20. In fact, we have performed the experiment mentioned by the reviewer to detect whether MPEL1 has ubiquitin ligase activity toward JAZ20 in previous, and the result showed that MPEL1 cannot promote the ubiquitination of JAZ20. However, this result was not included in the original manuscript because it is not consistent with the narrative logic of the study, which focuses on the SUMOylation mediated by MPEL1 rather than its

ubiquitination function. To clarify the presentation, we have revised the corresponding sections in the main text.

20. In Extended Fig. 12, certain panels appear to be similar from Extended Fig. 1.
Reply20: We have carefully verified the two figures, and there are no identical panels between Extended Figure 12 and Extended Figure 1. Extended Figure 1 shows the phenotypic results after infection with *Fusarium verticillioides*, while Extended Figure 12 presents the phenotypic results after infection with *Fusarium graminearum*, and the different pathogens are clearly labeled in both figures and figure legend.

21. The phenotype of plants expressing the MAPK16 K526R mutant should be described.
Reply21: This question is the same as Question 6. As explained in the reply to Question 6, we are currently unable to provide the phenotype of transgenic plants expressing the MAPK16 K526R mutant due to the long cycle required for the generation of maize transgenic materials and phenotypic verification. We would be grateful if you could kindly understand this practical experimental constraint.

22. Lines 250-251: The authors claim that SUMOylation of MAPK16 results in significantly slower degradation. However, based on Fig. b-c, the degradation rates appear comparable. Additionally, SUMOylated MAPK16 bands migrate near ~250 kDa, whereas previous blots show multiple SUMOylated species. This discrepancy should be addressed.
Reply22: Regarding the conclusion that SUMOylation of MAPK16 significantly slows its degradation rate, we would like to emphasize to the reviewer that this finding is not based on a subjective visual judgment of band intensity, but on statistical analyses of multiple repeated experiments (Fig.4b,c). We conducted quantitative analyses of the results of multiple independent experiments, and the data confirmed that the effect of SUMOylation on the stability of MAPK16 protein is statistically significant.

As for the slight differences in the SUMOylated bands observed in different experiments, we believe this may be caused by experimental fluctuations from technical operations such as Western Blot exposure time. As is well known to researchers familiar with Western Blot experiments, this technique is a semi-

quantitative method, and a variation of approximately 20% between different replicates is considered normal—this is exactly why we performed multiple repeated experiments and conducted statistical analyses. Additionally, to obtain a higher proportion of SUMOylated MAPK16, the in vitro SUMOylation reaction time for MAPK16 in Fig. 4b was longer than that in other in vitro SUMOylation experiments. It should be specially noted that the SUMOylated bands in the existing data cannot accurately reflect the actual molecular weight of MAPK16 poly-SUMOylation modified forms, so it is inappropriate to over-infer the modification forms based on the band positions.

23. The effect of MPEL1-mediated SUMOylation on MAPK16 stability should be validated using proteasome inhibitors.
Reply23: It should be noted that the experiment in Figure 4b used maize total protein extracts co-incubated with in vitro purified proteins, aiming to simulate the natural intracellular environment of protein degradation and compare the degradation rates of MAPK16 with or without SUMO-modification. Adding proteasome inhibitors would block the protein degradation pathway, which is contrary to the experimental purpose of this assay.

24. It is unclear why the authors tested whether MPEL1 SUMOylates JAZ20 in a MAPK16-dependent manner. If the hypothesis is that MAPK16 enhances MPEL1 activity, this should be tested using dose-dependent MAPK16 assays and by examining SUMOylation of other substrates such as ATG8b (Extended Fig. 6h).
Reply24: We acknowledge that there is a flaw in the narrative logic of this part in the original manuscript. In the initial version (before submitting to EMBO J) of the manuscript, the contents of Figure 2 and Figure 5 were merged, so JAZ20 was introduced relatively early. Then, based on the evidence of in planta interaction between MPEL1 and JAZ20 from the tobacco BiFC experiment, we subsequently analyzed whether JAZ20 can be recognized and SUMOylated by MPEL1. However, in the submitted version of the manuscript, JAZ20 has not been introduced at the position of Figure 4, leading to an illogical narrative. We have revised the structure and narrative logic of the manuscript to adjust the presentation position of relevant content and make the research story more coherent and logical. The corresponding content will be presented separately as **Appendix Fig. 7.**

25. In Extended Data Fig. 7, appropriate controls should demonstrate that MPEL1-Flag accumulates only in the presence of MAPK16-GFP and not with GFP alone. This should also be reflected in the co-localization images.

Reply25: It should be noted that you may have misunderstood the experimental purpose of Extended Data Figure 7. This experiment aims to verify that MPEL1-Flag promotes the accumulation of MAPK16-GFP, rather than that MAPK16-GFP induces the accumulation of MPEL1-Flag. The experimental control setting in the original manuscript is consistent with the experimental purpose, and the results are reliable.

26. In Fig. 5C, BiFC suggests that the C-terminus of MPEL1 interacts with the N-terminus of JAZ20; however, no interaction is observed in vitro. This interaction should be validated using in vivo assays in N. benthamiana.

Reply26: We would like to clarify to the reviewer that the labels MPEL1-cYFP and JAZ20-nYFP in the figure do not refer to the C-terminus of MPEL1 and the N-terminus of JAZ20, but indicate that the full-length sequences of MPEL1 and JAZ20 are fused to the cYFP and nYFP vectors, respectively. The experimental result clearly showed that the full-length MPEL1 interacts with the full-length JAZ20 in *N. benthamiana*. However, the subsequent in vitro Pull-down experiment failed to verify their direct interaction, indicating that the BiFC fluorescent signal observed in tobacco may result from an indirect interaction or a bridging effect mediated by other endogenous proteins in planta.

27. All lanes in immunoblots must be clearly labeled. For example, in Extended Fig. 8a, the identities of lanes 1 and 4 are missing.

Reply27: We acknowledge the insufficient labeling of partial immunoblot lanes in the original manuscript. Although the relevant information is clearly described in the figure legends, we will further improve the direct labeling of all lanes in the immunoblot figures to make the figures more intuitive and easy to interpret.

28. It is strange why we observe some level of JAZ20 degradation even in a phospho-null JAZ20 mutant and requires clarification.

Reply28: We think you are referring to Extended Figure 9b. The phenomenon of low-level degradation still observed in the $JAZ20^{T12AS13A}$ mutant is not an experimental

anomaly, but a result determined by the complexity of plant protein degradation regulation. Plant protein degradation involves multiple parallel pathways; even if the phosphorylation-mediated degradation pathway is blocked, other degradation mechanisms (such as ubiquitination-independent degradation pathways or other kinase-mediated phosphorylation degradation pathways) can still function to a certain extent. Therefore, the observation of low-level degradation of the $JAZ20^{T12AS13A}$ mutant is a completely reasonable normal biological phenomenon.

29. Several panels appear from the same experiment, such as Extended Fig. 8f, which is also shown in Fig. 6a.

Reply29: Extended Figure 8f and Figure 6a are not the same figure. Figure 6a mainly presents the validation results of the two key phosphorylation sites T12 and S13 in JAZ20, while Extended Figure 8f provides supplementary validation of other candidate phosphorylation sites (S4, T69, S76, S80, etc.) in JAZ20. The two figures show data for different phosphorylation sites (with partial overlap) and jointly support the comprehensive identification results of JAZ20 phosphorylation sites, which is a complementary experimental design rather than a simple repetition.

30. In Fig. 6b and d, there is a discrepancy in JAZ20 degradation. GST-JAZ20 degrades when incubated with WT total protein (panel b), whereas degradation is negligible in panel d. This inconsistency requires clarification.

Reply30: We acknowledge that there are slight differences in the Western Blot bands of GST-JAZ20 degradation between Figure 6b and Figure 6d, but this is not the "negligible degradation" in Figure 6d as described by you—the degradation of GST-JAZ20 in Figure 6d is clearly observable. As mentioned in the reply to Question 22, Western Blot is a semi-quantitative experimental technique, and a variation of approximately 20% between different experimental replicates is normal due to technical factors such as sample loading, protein extraction efficiency, and exposure time. This is the key reason why we performed multiple independent repeated experiments and conducted statistical analyses of the results. From the statistical data, we can see that the reduction in protein abundance at 60 min relative to 0 min is comparable in both figures. The Western Blot images shown in the manuscript are representative results of a single experiment, and the focus should be on the

statistically significant results of multiple repeated experiments rather than the slight differences in individual band intensity.

31. In Fig. 7a, showing JAZ20 stability in the presence of MAPK16 and MPEL1, the time point used should be specified. In vitro assays at 60 minutes show a reduction in JAZ20 in the presence of MAPK16.

Reply31: The experiment in Figure 7a involves the co-expression of MAPK16, MPEL1, and JAZ20 in tobacco leaves, and laser confocal detection was performed after 36 hours of normal expression—this is an in planta experimental system, which is completely different from the in vitro experimental conditions (60 minutes incubation). The different experimental systems and time points are determined by the experimental design and research purpose, and we have added a clear description of the experimental time point and system in the figure legend to avoid confusion.

32. In Fig. 7c, inclusion of a control showing JAZ20 stability in the absence of MPEL1 is essential to establish the role of MPEL1 as an E4 ligase.

Reply32: We would like to clarify to the reviewer that our research has never claimed that MPEL1 is an E4 ligase; our conclusion from the beginning to the end is that MPEL1 is a SUMO E3 ligase rather than an E4 ligase. The experimental purpose of Figure 7c is to prove that MPEL1 can promote the phosphorylation of JAZ20 by MAPK16, not to verify the E4 ligase function of MPEL1. In our previous results, we have already demonstrated that MPEL1 can promote the SUMOylation of MAPK16, and that MAPK16 can phosphorylate JAZ20. Here, we only aimed to investigate how SUMOylation of MAPK16 affects its phosphorylation of JAZ20. The experimental design and control setting of this figure are consistent with its research purpose.

33. In Fig. 7a, the cytoplasmic fraction of JAZ20 is poorly represented in the GFP channel and appears predominantly nuclear. Quantification of the nuclear-to-cytoplasmic ratio is recommended.

Reply33: We appreciate this suggestion. Fig. 7b presents the quantitative analysis of proteins from the corresponding experimental groups in Fig. 7a using Western blot. We have attempted to perform the nuclear-to-cytoplasmic ratio quantification analysis as suggested by the reviewer. However, this quantification method cannot well reflect the differences in protein levels between different experimental groups. The current

detection method in the manuscript can not only reflect the subcellular distribution (nuclear and cytoplasmic) of JAZ20 protein but also show the differences in the total protein abundance of JAZ20 between different groups, which is more suitable for the research purpose of this experiment (analyzing the regulation of JAZ20 stability and distribution by MAPK16).

34. The functional relevance of MAPK16-mediated phosphorylation and degradation of ZIM1 is unclear and is not discussed. This should be addressed.
Reply34: We have provided sufficient experimental evidence for the phosphorylation of ZIM1 (JAZ17) by MAPK16 and the subsequent promotion of ZIM1 degradation in the manuscript. We infer that the you hope us to provide phenotypic data of ZIM1-related transgenic materials. Considering that the functions of JAZ family members have been extensively reported in numerous previous studies and their action mechanisms are highly conserved, we did not generate ZIM1 transgenic materials after thoroughly elucidating the regulatory mechanism of MAPK16 on JAZ20 (the key JAZ family member in this study). We believe that this does not affect the core findings and conclusions of our research, as the conserved regulatory mechanism of MAPK16 on JAZ proteins has been sufficiently verified through the in-depth analysis of JAZ20.

35. Lines 488-491: AtSTUbL lacks ubiquitin ligase activity is an overstatement. As mentioned earlier proper validation needs to be made with all Ub E2 ligases before coming to a conclusion.
Reply35: We acknowledge that the relevant statement in Lines 488-491 is an overstatement and inappropriate. We have revised this statement in the resubmitted manuscript as the replies to Q11 and Q16.

36. SUMO E2 enzymes can also enhance SUMOylation in a dose-dependent manner even in the absence of E3 ligases.
Reply36: We fully agree with this view. However, we currently do not fully understand the specific research significance and experimental design of this suggested assay for our study.

Referee #2:

In this manuscript, Feng and colleagues investigate the molecular basis of maize resistance to Fusarium pathogens. Their study begins with the observation that MAPK16 functions as a positive regulator of immunity against F. verticillioides. The authors then show that MPEL1, acting as a SUMO E3 ligase, stabilizes MAPK16 through SUMOylation. This stabilized MAPK16 then phosphorylates JAZ20, leading to its degradation, activation of defense genes, and ultimately enhanced resistance. The study is well-executed and provides strong evidence for these interactions that are initially identified through yeast-two-hybrid assays and subsequently validated by bifluorescence complementation, co-immunoprecipitation, and pull-down experiments. Furthermore, the authors pinpointed specific SUMOylation and phosphorylation sites using mutant analysis and/or proteomics. The localization of both proteins and interactions is determined and functional validation in maize confirms the role of these interactions in pathogen resistance.
This work thus provides important mechanistic insights into plant immunity obtained and confirmed through a variety of techniques. This work notably identifies a post-translational regulatory pathway that enhances defense responses without compromising growth. This is particularly relevant to agricultural applications aimed at improving crop resilience. Beyond plants, this study contribute to a broader understanding of kinase regulation and protein modification mechanisms like SUMOylation and Ubiquitination, thereby appealing to researchers in diverse fields.

Overall, I strongly support the publication of this article in EMBO Journal with the following minor concerns:
1. Given recent literature highlighting the complexity in identifying true SUMO E3 ligases (e.g. TOPORS was initially shown to display SUMO E3 activity but a latter study showed that this activity was modest and independent of the RING domain with the current view being that TOPORS rather acts as a StUbL), it would be valuable to further characterize MPEL1's SUMOylation activity. Specifically, investigating whether the RING domain (residues 176-end) and/or the N-terminal SIM region (residues 1-175) are essential for this activity would provide additional information regarding the molecular determinants of the SUMO E3 activity. This could be performed using in vitro SUMOylation assay as performed in Fig. 3e.
Reply1: We sincerely appreciate your positive comments on our work, which have been a great encouragement to us. Following your suggestion, we separately assayed the SUMO E3 ligase activity of the RING domain (residues 176 to the C-terminus) and the N-terminal SIM region (residues 1–175). The results demonstrated that the SUMO E3 ligase function of MPEL1 is dependent on its full-length protein; neither the isolated RING domain nor the N-terminal SIM region alone can promote the SUMOylation of MAPK16. The relevant results are presented in **Fig. EV2E.** For your convenience, the important modifications in the manuscript have been highlighted in yellow.

2. Quantifying the extent of MAPK16 stabilization by MPEL1 in Extended Data Fig. 7b would enhance the clarity of these results.
Reply2: Thank you for your suggestion. We have supplemented the quantified values

of the band signals in the figure, and these two panels have been incorporated into **Figure 4.**

3. Mention that the SUMO and Ubiquitin constructs used in yeast-two-hybrid experiments can be conjugated (i.e. they both end with GG). These experiments (e.g. Fig. 3b) can thus report covalent and nonconvalent interactions.
Reply3: The SUMO and Ubiquitin constructs used in our yeast two-hybrid (Y2H) and pull-down assays do terminate with a glycine-glycine (GG) motif, and we fully agree with your viewpoint. In the Y2H assays, the binding of MPEL1 and STUbL1 to Ubiquitin may be direct, whereas the interaction between SUMO and MPEL1 is largely dependent on the covalent conjugation of SUMO to the E2 enzyme.

4. The interaction between MPEL1 and MAPK16 in the cytoplasm/membrane is intriguing given that SUMOylation typically occurs in the nucleus. Further discussion of this observation could be insightful.
Reply4: We fully concur with your suggestion. In fact, we had discussed this observation in the previous version of the manuscript before its submission to the EMBO Journal, and we have now supplemented the relevant discussion as you recommended in section "***MPEL1*** **is a new SUMO ligase**".

5. Providing the exact residue boundaries for the N- and C-terminal truncations used in the study and including these numbers in Fig. 2a, along with visual representations of the truncations on protein schemes would improve clarity for readers.
Reply5: Following your suggestion, we have revised Figure 2a accordingly.

6. In Extended Data Fig. 5, the name of the protein (MPEL) appears to have been mistakenly included at the beginning of the sequence. Please remove "MPEL" at the beginning of the ZmMPEL1 sequence.
Reply6: We greatly appreciate your meticulous review and have corrected this error.

7. In Extended data Figure 1b, is the overexpressed MAPK16 tagged? Its migration suggests a higher molecular weight than the native protein (assuming the native protein is the band below 60kDa).
Reply7: The overexpressed MAPK16 was fused with a Flag tag, and an anti-Flag tag antibody was used for the Western blot detection. The band with a molecular weight below 60 kDa present in each lane is a non-specific band.

8. Indicate "Time (min)" in panels C and E of Fig. 6.
Reply8: This has been revised accordingly.

Referee #3:

This is a high-quality and mechanistically sophisticated study that integrates

SUMOylation, MAPK signaling, and jasmonate repression into a coherent plant defense pathway with clear agronomic relevance. The identification of MPEL1 as a non-canonical SUMO E3 ligase, its role in stabilizing MAPK16, and the downstream MAPK16-JAZ20 phosphorylation-degradation module represent a genuinely novel conceptual advance. Overall, the work is strong and timely, and I recommend acceptance pending revision.

Major concerns

1. The authors conclude that MPEL1 functions as a SUMO E3 ligase. While the data supporting substrate SUMOylation are compelling, direct biochemical evidence of intrinsic E3 ligase activity remains limited. Additional clarification or experimental support distinguishing MPEL1 from adaptor-like or co-factor roles in SUMOylation would strengthen this conclusion.

Reply1: We sincerely appreciate your positive comments on our work, which have been a great encouragement to us. Your suggestion is highly constructive, and Referee #2 raised the same point that it would be valuable to further characterize MPEL1's SUMOylation activity. Specifically, investigating whether the RING domain (residues 176-end) and/or the N-terminal SIM region (residues 1-175) are essential for this activity would provide additional information regarding the molecular determinants of the SUMO E3 activity. Following both your suggestions, we separately assayed the SUMO E3 ligase activity of the RING domain (residues 176 to the C-terminus) and the N-terminal SIM region (residues 1–175). The results demonstrated that the SUMO E3 ligase function of MPEL1 is dependent on its full-length protein; neither the isolated RING domain nor the N-terminal SIM region alone can promote the SUMOylation of MAPK16. The relevant results are presented in **Fig. EV2E.** For your convenience, the important modifications in the manuscript have been highlighted in yellow.

2. The manuscript proposes that SUMOylation of MAPK16 at K526 is functionally important for disease resistance in planta. However, no genetic complementation or resistance assays using a MAPK16K526R variant are presented. Such experiments would be important to directly test whether this modification is required for the resistance phenotype.

Reply2: We fully acknowledge that providing phenotypic data for the transgenic lines of the *mapk16* mutant complemented with MAPK16(K526R) would greatly enhance the completeness of this study. Regrettably, we are unable to provide the phenotype of transgenic plants expressing the $MAPK16^{K526R}$ mutant at present, as the generation of such site-directed mutant transgenic materials takes approximately one year, and the acquisition of related phenotypic data will require an additional 1-2 years due to the growth cycle of maize and experimental verification. While, we have added in vivo assays to detect the deSUMOylation of MAPK16 after mutating the lysine at position 526 to arginine (K526R). Results showed that $MAPK16^{K526R}$ cannot be SUMOylated

in vivo, with detailed data presented in **Fig. 4B.** We would be grateful for your understanding of this practical experimental limitation associated with generating and characterizing the MAPK16^{K526R}-complemented *mapk16* mutant lines.

3. The authors show that MAPK16 can also phosphorylate ZIM1/JAZ17, and proteomic analyses indicate that multiple JAZ proteins are affected. It remains unclear why JAZ20 is positioned as the central regulatory node in this pathway rather than one of several functionally redundant or partially overlapping JAZ proteins. A clearer rationale, supported by genetic or functional specificity, would improve the conceptual clarity of this section.

Reply3: Thank you for your suggestion, and we fully agree with your viewpoint. We wish to clarify that we did not characterize JAZ20 as the central regulatory node in this pathway. Instead, the regulatory mechanism of JAZ20 by MAPK16 was investigated in greater detail in our study simply because JAZ20 was the first MAPK16-interacting protein identified via Y2H screening, and JAZ17 was not isolated in this initial screening. Our results do not exclude the possibility that MAPK16 may also regulate maize resistance to Fusarium pathogens through other JAZ family members.

4. The cell-death phenotype observed in tobacco is intriguing but currently underdeveloped. It is not clear whether this phenotype reflects a biologically relevant defense response or an artifact of heterologous overexpression. Additional discussion or supporting evidence would help to contextualize these observations.

Reply4: Following your suggestion, we have supplemented the relevant discussion on this observation. Briefly, we propose that the role of MPEL1 in disease resistance may be complex, as evidenced by the disease-resistant phenotype of its loss-of-function mutant. In addition to MAPK16 and ATG8b, MPEL1 may have a broader range of substrates, some of which may enhance plant disease resistance while others may have the opposite effect. The cell death induced by MPEL1 overexpression may result from its direct interaction with regulatory factors involved in cell apoptosis, or from its modulation of a stronger defense response pathway (e.g., the ETI pathway). We have supplemented the relevant discussion in section "***MPEL1* is a new SUMO ligase**".

Minor concerns

5. Language and clarity require minor editing throughout. Examples include:

◦ "bind to" → "binds to" (multiple instances)

◦ "may not dependent on ROS" → "may not be dependent on ROS"

◦ "pathwasys" → "pathways" (around line 399)

Please also ensure consistent terminology throughout the manuscript, particularly for "SUMO E3 ligase" versus "SUMO ligase," and "STUbL" versus "STUBL."

Reply5: We have carefully checked the entire manuscript and revised all imprecise expressions and inconsistent terminologies.

Prof. Yanli Lu
Sichuan Agricultural University
State Key Laboratory of Crop Gene Exploration and Utilization in Southwest China
Chengdu, Sichuan 611130
China

28th Apr 2026

Re: EMBOJ-2026-123473R1
A new SUMO ligase synergizes with MAPK16 to regulate resistance against Fusarium pathogens

Dear Dr. Lu,

Thank you for submitting your revised manuscript on a new plant SUMO ligase to The EMBO Journal. Two of the original referees have now assessed it once more (see below), and were overall satisfied with the revisions. We shall therefore be happy to accept this work for publication, as soon as the following remaining editorial issues will have been addressed:

- I would suggest making the title somewhat more explicit and clear, including naming of the relevant model, e.g.
MPEL1 is a SUMO ligase that synergizes with MAPK16 to regulate maize resistance against Fusarium pathogens
A new plant SUMO ligase, MPEL1, synergizes with MAPK16 to regulate resistance against Fusarium pathogens

- Similarly, I would suggest to alter the second sentence in the abstract to:
Here, we report MPEL1 as a new SUMO ligase in maize, which paradoxically exhibits SUMO ligase activity despite its sequence homology to SUMO-targeted ubiquitin ligases (STUbL).

- Please rename the Conflict of Interest section into "Disclosure and Competing Interests Statement", in accordance with our updated Guide to Authors (https://www.embopress.org/competing-interests)

- As we are switching from a free-text author contribution statement towards a more formal statement based on Contributor Role Taxonomy (CRediT) terms, please remove the present Author Contribution section and instead specify each author's contribution(s) directly in the Author Information page of our submission system during upload of the final manuscript. See https://casrai.org/credit/ for more information.

- Please carefully go through the reference list and make sure that all references have their complete citation information - including year, journal name & volume, and page/elocator numbers - such information is currently missing for several of them. Also, the journal name for the Christensen et al reference appears to include some unusual characters.

- Please make sure to fully fill in our author checklist, which seems to at least lack the manuscript-ID number.

- Please check that each figure panel is referenced at least once in the text - some callouts for Fig. 8 panels are currently missing. If helpful, you may simply replace the call-out for the complete 'Fig. 8' with a call-out stating 'Fig. 8A-I, before referencing individual panels.

- Please remove the EV table legends/titles from the main manuscript text, they should only be present in the respective XLSX files (ideally, within a separate 'Legends' tab in each workbook). Furthermore, while Tables EV1/2 could remain EV Tables, the others (Tables EV3-6) should be converted/renamed into 'Dataset EV1-4', given their layout and the type of data contained in them.

- Please upload the Appendix file in PDF format, and make sure to remove any text highlights of revisions before conversion and upload.

- Please provide suggestions for a short 'blurb' text prefacing and summing up the study in two sentences (max. 250 characters), followed by 3-5 one-sentence 'bullet points' (maybe using the 'Highlights' currently included in the manuscript text itself); they will form the basis of an editor-written 'Synopsis' accompanying the online version of the article. Please also upload a synopsis image, which can be used as a "visual title" for the synopsis section of your paper (maybe based on Fig 8I?). The image should be in JPG format with the modest dimensions of exactly 550 pixels wide and 300 pixels high.

- Finally, our data editors have raised the following queries regarding figures, data, and legends; I would appreciate if you briefly answered to them in the cover letter of your final submission, and made the requested text modifications with changes/additions highlighted via the "Track changes" option, to facilitate our final checking:
1) In the Data Availability section, please provide a specific URL for the database in which the deposited PRJCA032969 dataset

is accessible.
2) Please state the exact p value in the legends of figure 1H
3) Please define the error bars in the legends of figures 4F, EV4 G

I am therefore returning the manuscript to you for a final round of minor revision, to allow you to make these modifications and upload the revised files. Once we will have received them, we should hopefully be ready to swiftly proceed with formal acceptance and production of the manuscript.

With kind regards,
Hartmut Vodermaier

Hartmut Vodermaier, PhD
Senior Editor, The EMBO Journal
h.vodermaier@embojournal.org

*** PLEASE NOTE: All revised manuscript are subject to initial checks for completeness and adherence to our formatting guidelines. Revisions may be returned to the authors and delayed in their editorial re-evaluation if they fail to comply to the following requirements. As a first step please read our guidelines for revised submissions:
https://link.springer.com/journal/44318/submission-guidelines#cms-Revised-submissions

1) Every manuscript requires a Data Availability section (even if only stating that no deposited datasets are included). Primary datasets or computer code produced in the current study have to be deposited in appropriate public repositories prior to resubmission, and reviewer access details provided in case that public access is not yet allowed.

2) Each figure legend must specify
- size of the scale bars that are mandatory for all micrograph panels
- the statistical test used to generate error bars and P-values
- the type error bars (e.g., S.E.M., S.D.)
- the number (n) and nature (biological or technical replicate) of independent experiments underlying each data point
- Figures may not include error bars for experiments with n<3; scatter plots showing individual data points should be used instead.

3) Revised manuscript text (including main tables, and figure legends for main and EV figures) has to be submitted as editable text file (e.g., .docx format). We encourage highlighting of changes (e.g., via text color) for the referees' reference.

4) Each main and each Expanded View (EV) figure should be uploaded as individual production-quality files (preferably in .eps, .tif, .jpg formats). For suggestions on figure preparation/layout, please refer to our Figure Preparation Guidelines:
https://media.springernature.com/original/springer-cms/rest/v1/content/27825798/data/v1

5) Point-by-point response letters should include the original referee comments in full together with your detailed responses to them (and to specific editor requests if applicable), and also be uploaded as editable (e.g., .docx) text files.

6) Please complete our Author Checklist, and make sure that information entered into the checklist is also reflected in the manuscript; the checklist will be available to readers as part of the Review Process File.

7) All authors listed as (co-)corresponding need to deposit, in their respective author profiles in our submission system, a unique ORCiD identifier linked to their name. Please see our Guide to Authors for detailed instructions.

8) Please note that supplementary information at EMBO Press has been superseded by the 'Expanded View' for inclusion of additional figures, tables, movies or datasets; with up to five EV Figures being typeset and directly accessible in the HTML version of the article.

9) To facilitate reproducibilty and cross-laboratory adoption of methodologies, please structure the Materials & Methods section as outlined in our guide to authors, including a completed Reagents and Tools Table.

10) Digital image enhancement is acceptable practice, as long as it accurately represents the original data and conforms to community standards. If a figure has been subjected to significant electronic manipulation, this must be clearly noted in the figure legend and/or the 'Materials and Methods' section. The editors reserve the right to request original versions of figures and the original images that were used to assemble the figure. Finally, we generally encourage uploading of numerical as well as gel/blot image source data.

At EMBO Press we ask authors to provide source data for the main manuscript figures. You will receive a separate email with instructions for providing source data with your revised manuscript, including how to upload and organize the files.

In the interest of ensuring the conceptual advance provided by the work, we recommend submitting a revision within 3 months (27th Jul 2026). Please discuss the revision progress ahead of this time with the editor if you require more time to complete the revisions. Use the link below to submit your revision:

Link Not Available

Referee #2:

The authors have addressed all of my concerns. They clarified several parts of the manuscript and they performed additional quantifications and experiments, notably performing SUMOylation assays using fragments of MPEL1 containing either the SIM or RING domain. I am fully supportive of publication.

On a very minor note, certain - and + moved in the PDF version of the Appendix (Fig S5 to S7) but their position is correct in the provided DOCX. The sequence alignement of Fig S4 could benefit from the use of a monospaced font.

Referee #3:

This is an exceptional manuscript that reports a major advance in our understanding of plant immunity, SUMOylation, and MAP kinase signaling. The discovery of MPEL1's function is particularly novel and will be of broad interest. The experimental evidence is rigorous and strongly supports the main conclusions. The authors have addressed all of my comments, and I am satisfied with their responses. I have only a few suggestions for the authors' consideration.
1. A brief discussion of whether the N-terminus harbors a novel SUMO-interacting motif or is required structurally would strengthen the mechanistic model.
2. The lack of a significant ear rot phenotype in mpel1 mutants is intriguing. Providing expression data for MPEL1 transcript/protein in ear, stalk, and leaf tissues (both basal and post-infection) would ground the discussion in data.
3. A few minor grammatical corrections are needed (e.g., "Further investigation have" → "has"; "rather than altered" → "rather than alter").

EMBO Press Author Checklist

Corresponding Author Name: Yanli Lu
Journal Submitted to: The EMBO Journal
Manuscript Number: EMBOJ-2026-123473R1

USEFUL LINKS FOR COMPLETING THIS FORM
The EMBO Journal - Author Guidelines
EMBO Reports - Author Guidelines
Molecular Systems Biology - Author Guidelines
EMBO Molecular Medicine - Author Guidelines

Reporting Checklist for Life Science Articles (updated January
This checklist is adapted from Materials Design Analysis Reporting (MDAR) Checklist for Authors. MDAR establishes a minimum set of requirements in transparent reporting in the life sciences (see Statement of Task: 10.31222/osf.io/9sm4x). Please follow the journal's guidelines in preparing your
Please note that a copy of this checklist will be published alongside your article.

Abridged guidelines for figures
1. Data
The data shown in figures should satisfy the following conditions:
- the data were obtained and processed according to the field's best practice and are presented to reflect the results of the experiments in an accurate and unbiased manner.
- ideally, figure panels should include only measurements that are directly comparable to each other and obtained with the same assay.
- plots include clearly labeled error bars for independent experiments and sample sizes. Unless justified, error bars should not be shown for technical
- if n<5, the individual data points from each experiment should be plotted. Any statistical test employed should be justified.
- Source Data should be included to report the data underlying figures according to the guidelines set out in the authorship guidelines on Data

2. Captions
Each figure caption should contain the following information, for each panel where they are relevant:
- a specification of the experimental system investigated (eg cell line, species name).
- the assay(s) and method(s) used to carry out the reported observations and measurements.
- an explicit mention of the biological and chemical entity(ies) that are being measured.
- an explicit mention of the biological and chemical entity(ies) that are altered/varied/perturbed in a controlled manner.
- the exact sample size (n) for each experimental group/condition, given as a number, not a range;
- a description of the sample collection allowing the reader to understand whether the samples represent technical or biological replicates (including how many animals, litters, cultures, etc.).
- a statement of how many times the experiment shown was independently replicated in the laboratory.
- definitions of statistical methods and measures:
  - common tests, such as t-test (please specify whether paired vs. unpaired), simple χ2 tests, Wilcoxon and Mann-Whitney tests, can be unambiguously identified by name only, but more complex techniques should be described in the methods section;
  - are tests one-sided or two-sided?
  - are there adjustments for multiple comparisons?
  - exact statistical test results, e.g., P values = x but not P values < x;
  - definition of 'center values' as median or average;
  - definition of error bars as s.d. or s.e.m.

Please complete ALL of the questions below.
Select "Not Applicable" only when the requested information is not relevant for your study.

Materials

Newly Created Materials	Information included in the manuscript?	In which section is the information available? (Reagents and Tools Table, Materials and Methods, Figures, Data Availability Section)
New materials and reagents need to be available; do any restrictions apply?	Yes	Methods：Plant materials and growth conditions/Figure EV1A-B, Appendix Figure S9A-B

Antibodies	Information included in the manuscript?	In which section is the information available? (Reagents and Tools Table, Materials and Methods, Figures, Data Availability Section)
For **antibodies** provide the following information:<br>- Commercial antibodies: RRID (if possible) or supplier name, catalogue number and or/clone number<br>- Non-commercial: RRID or citation	Yes	Methods, Reagents and tools table

DNA and RNA sequences	Information included in the manuscript?	In which section is the information available? (Reagents and Tools Table, Materials and Methods, Figures, Data Availability Section)
**Short novel DNA or RNA including primers, probes**: provide the sequences.	Yes	Dataset EV4

Cell materials	Information included in the manuscript?	In which section is the information available? (Reagents and Tools Table, Materials and Methods, Figures, Data Availability Section)
**Cell lines:** Provide species information, strain. Provide accession number in repository **OR** supplier name, catalog number, clone number, and/**OR** RRID.	Not Applicable	N/A
**Primary cultures:** Provide species, strain, sex of origin, genetic modification status.	Not Applicable	N/A
Report if the cell lines were recently **authenticated** (e.g., by STR profiling) and tested for mycoplasma contamination.	Not Applicable	N/A

Experimental animals	Information included in the manuscript?	In which section is the information available? (Reagents and Tools Table, Materials and Methods, Figures, Data Availability Section)
**Laboratory animals or Model organisms:** Provide species, strain, sex, age, genetic modification status. Provide accession number in repository **OR** supplier name, catalog number, clone number, **OR** RRID.	Not Applicable	N/A
**Animal observed in or captured from the field:** Provide species, sex, and age where possible.	Not Applicable	N/A
Please detail **housing and husbandry conditions**.	Not Applicable	N/A

Plants and microbes	Information included in the manuscript?	In which section is the information available? (Reagents and Tools Table, Materials and Methods, Figures, Data Availability Section)
**Plants:** provide species and strain, ecotype and cultivar where relevant, unique accession number if available, and source (including location for collected wild specimens).	Yes	Methods：Plant materials and growth conditions
**Microbes:** provide species and strain, unique accession number if available, and source.	Yes	Methods：Microbial strains and culture conditions

Human research participants	Information included in the manuscript?	In which section is the information available? (Reagents and Tools Table, Materials and Methods, Figures, Data Availability Section)
If collected and within the bounds of privacy constraints report on age, sex and gender or ethnicity for all study participants.	Not Applicable	N/A

Core facilities	Information included in the manuscript?	In which section is the information available? (Reagents and Tools Table, Materials and Methods, Figures, Data Availability Section)
If your work benefited from core facilities, was their service mentioned in the acknowledgments section?	Yes	ACKNOWLEDGMENTS

Design

Study protocol	Information included in the manuscript?	In which section is the information available? (Reagents and Tools Table, Materials and Methods, Figures, Data Availability Section)
If study protocol has been **pre-registered, provide DOI in the manuscript**. For clinical trials, provide the trial registration number **OR** cite DOI.	Not Applicable	N/A
Report the **clinical trial registration number** (at ClinicalTrials.gov or equivalent), where applicable.	Not Applicable	N/A

Laboratory protocol	Information included in the manuscript?	In which section is the information available? (Reagents and Tools Table, Materials and Methods, Figures, Data Availability Section)
Provide DOI OR other citation details if **external detailed step-by-step protocols** are available.	Yes	Methods

Experimental study design and statistics	Information included in the manuscript?	In which section is the information available? (Reagents and Tools Table, Materials and Methods, Figures, Data Availability Section)
Include a statement about **sample size** estimate even if no statistical methods were used.	Yes	Methods, Figures
Were any steps taken to minimize the effects of subjective bias when allocating animals/samples to treatment (e.g. **randomization procedure**)? If yes, have they been described?	Yes	Methods：Plant materials and growth conditions
Include a statement about **blinding** even if no blinding was done.	Not Applicable	N/A
Describe **inclusion/exclusion criteria** if samples or animals were excluded from the analysis. Were the criteria pre-established?<br><br>If sample or data points were omitted from analysis, report if this was due to attrition or intentional exclusion and provide justification.	Not Applicable	N/A
For every figure, are **statistical tests** justified as appropriate? Do the data meet the assumptions of the tests (e.g., normal distribution)? Describe any methods used to assess it. Is there an estimate of variation within each group of data? Is the variance similar between the groups that are being statistically compared?	Yes	Methods, Figures

Sample definition and in-laboratory replication	Information included in the manuscript?	In which section is the information available? (Reagents and Tools Table, Materials and Methods, Figures, Data Availability Section)
In the figure legends: state number of times the experiment was **replicated** in laboratory.	Yes	Methods, Figures
In the figure legends: define whether data describe **technical or biological replicates**.	Yes	Methods, Figures

Ethics

Ethics	Information included in the manuscript?	In which section is the information available? (Reagents and Tools Table, Materials and Methods, Figures, Data Availability Section)
Studies involving **human participants**: State details of **authority granting ethics approval** (IRB or equivalent committee(s), provide reference number for approval.	Not Applicable	N/A
Studies involving **human participants**: Include a statement confirming that **informed consent** was obtained from all subjects and that the experiments conformed to the principles set out in the WMA Declaration of Helsinki and the Department of Health and Human Services Belmont Report.	Not Applicable	N/A
Studies involving **human participants:** For publication of **patient photos**, include a statement confirming that consent to publish was obtained.	Not Applicable	N/A
Studies involving experimental **animals**: State details of **authority granting ethics approval** (IRB or equivalent committee(s), provide reference number for approval. Include a statement of compliance with ethical regulations.	Not Applicable	N/A
Studies involving **specimen and field samples:** State if relevant **permits** obtained, provide details of authority approving study; if none were required, explain why.	Not Applicable	N/A

Dual Use Research of Concern (DURC)	Information included in the manuscript?	In which section is the information available? (Reagents and Tools Table, Materials and Methods, Figures, Data Availability Section)
Could your study fall under dual use research restrictions? Please check biosecurity documents and list of **select agents and toxins** (CDC): https://www.selectagents.gov/sat/list.htm	Not Applicable	N/A
If you used a select agent, is the security level of the lab appropriate and reported in the manuscript?	Not Applicable	N/A
If a study is subject to dual use research of concern regulations, is the name of the **authority granting approval and reference number** for the regulatory approval provided in the manuscript?	Not Applicable	N/A

Reporting

The MDAR framework recommends adoption of discipline-specific guidelines, established and endorsed through community initiatives. Journals have their own policy about requiring specific guidelines and recommendations to complement MDAR.

Adherence to community standards	Information included in the manuscript?	In which section is the information available? (Reagents and Tools Table, Materials and Methods, Figures, Data Availability Section)
State if relevant guidelines or checklists (e.g., **ICMJE, MIBBI, ARRIVE, PRISMA**) have been followed or provided.	Not Applicable	N/A
For **tumor marker prognostic studies,** we recommend that you follow the **REMARK** reporting guidelines (see link list at top right). See author guidelines, under 'Reporting Guidelines'. Please confirm you have followed these guidelines.	Not Applicable	N/A
For **phase II and III randomized controlled trials**, please refer to the **CONSORT** flow diagram (see link list at top right) and submit the CONSORT checklist (see link list at top right) with your submission. See author guidelines, under 'Reporting Guidelines'. Please confirm you have submitted this list.	Not Applicable	N/A

Data Availability

Data availability	Information included in the manuscript?	In which section is the information available? (Reagents and Tools Table, Materials and Methods, Figures, Data Availability Section)
Have **primary datasets** been deposited according to the journal's guidelines (see 'Data Deposition' section) and the respective accession numbers provided in the Data Availability Section?	Yes	Data Availability Section
Were **human clinical and genomic datasets** deposited in a public access-controlled repository in accordance to ethical obligations to the patients and to the applicable consent agreement?	Not Applicable	N/A
Are **computational models** that are central and integral to a study available without restrictions in a machine-readable form? Were the relevant accession numbers or links provided?	Not Applicable	N/A
If publicly available data were reused, provide the respective **data citations in the reference list**.	Not Applicable	N/A
